# Dynamics and Control of Dual Active Sites in Co-Substituted Ni Coordination Polymers for Enhanced Oxygen Evolution Catalysis

**DOI:** 10.1021/jacs.5c12205

**Published:** 2026-01-20

**Authors:** Yonggui Zhao, Nanchen Dongfang, Rolf Erni, Marcella Iannuzzi, Greta R. Patzke

**Affiliations:** 1 Department of Chemistry, 27217 University of Zurich, Winterthurerstrasse 190, CH-8057 Zurich, Switzerland; 2 Electron Microscopy Center, Empa, Swiss Federal Laboratories for Materials Science and Technology, Überlandstrasse 129, CH-8600 Dübendorf, Switzerland

## Abstract

Facilitating the kinetically demanding oxygen evolution reaction (OER) is essential for the sustainable conversion of renewable energy into chemical fuels. However, precisely unraveling the dynamics of active species and sites during the OER remains a significant challenge. Herein, we constructed a series of Co-substituted Ni coordination polymers (Ni-CPs) for the OER. Complementary surface-/bulk-sensitive *operando* time-resolved spectroscopic monitoring enables detailed mechanistic insight into the critical role of partial Co incorporation in modulating the local coordination geometry of Ni centers and thereby promoting the intrinsic OER kinetics. Our results reveal that controlled Co substitution in Ni-CPs facilitates the generation of a substantial fraction of (Ni, Co)­(IV) species, which activate O–O bond formation atop the catalytically active Ni^IV^-O-Co^IV^ moieties. These key findings are further supported by kinetic isotopic effect studies and density functional theory calculations, in which the OER in Ni_3_Co_1_-CPs proceeds via an oxo-radical coupling mechanism, with deprotonation preferentially occurring at the Ni sites. Consequently, the engineered Ni_3_Co_1_-CPs exhibit enhanced OER activity compared to their oxide counterparts, along with durable electrochemical stability for over 4000 h. This study not only offers detailed mechanistic insights into the dynamics of active species and sites but also highlights their critical role in optimizing the OER kinetics.

## Graphical abstract

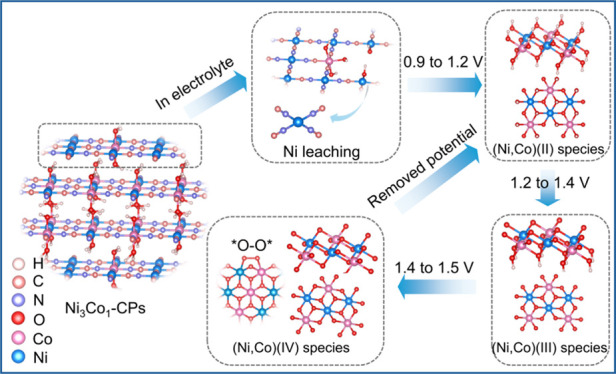

## Introduction

The oxygen evolution reaction (OER) serves as a central process in various electrochemical conversion systems.^1–3^ Development of efficient OER catalysts relies on a comprehensive mechanistic understanding of the underlying reaction kinetics. To date, three primary OER mechanism pathways in alkaline media have been explored, namely the adsorbate evolution mechanism (AEM), lattice oxygen mechanism (LOM), and oxo-radical coupling or oxide-path mechanism (OPM). For the conventional AEM pathway, the OER proceeds via the formation of a key *OOH intermediate that features a highly correlated adsorption strength with other OER intermediates, resulting in a limited theoretical OER overpotential of ca. 0.37 V.^4–6^ In contrast, the LOM pathway involves direct participation of lattice oxygen, enabling the circumvention of the intrinsic scaling relations and offering a lower energy barrier for the release of O_2_. However, the activation of lattice oxygen requires highly oxidized metal centers and considerable structural flexibility, often leading to compromised structural stability during long-term OER operation.^4,7–9^ Compared to the AEM and LOM, the generation of O_2_ in the OPM arises from the direct coupling of oxo-radicals. This bypasses the formation of *OOH intermediate with a breaking of the scaling relation, while simultaneously preserving structural integrity during the OER cycling process.^9–13^ Notably, the O–O bond formation via an OPM pathway typically involves the assembly of high-valent M-O-M moieties (M: metal centers).^9,10,13,14^ Therefore, the rational design of catalysts that maximizes the formation of high-valent M-O-M moieties is crucial for activating the OPM pathway, thereby enabling both enhanced OER activity and long-term durability.

Earth-abundant 3d transition metal-based catalysts have recently emerged as promising alternatives for the OER under alkaline conditions.^15–19^ Advanced *operando* spectroscopic monitoring has revealed that these catalysts commonly undergo electrochemically driven bulk/surface restructuring, giving rise to the formation of high-valent M-O-M motifs that facilitate O_2_ release.^2,20–22^ Optimization of such an electrochemically driven restructuring process is strongly influenced by the initial compound compositions and the local coordination geometry of active metal centers.^22–27^ For example, a recent study on monolayer Ni­(OH)_2_ demonstrated that *in situ*-generated Ni­(IV) species contribute significantly to the observed OER activity.^24^ Meanwhile, partial Co incorporation enables flexible valence state changes of Ni and Co centers during the OER process, thereby facilitating the construction of highly active Ni^IV^-O-Co^IV^ moieties with the enhanced OER activity.

Although significant progress has been achieved with oxide/hydroxide-based catalysts for the OER, their catalytic performance is often limited by the sluggish formation and poor stabilization of high-valent active species, which arises from their inherently heterogeneous coordination environments.^4,17,28^ In sharp contrast, transition metal-based coordination polymers (MCPs) offer a structurally distinct platform for the OER, in which metal centers are linked by inorganic or organic ligands and exhibit a well-defined coordination environment.^2,26,29^ Such molecularly ordered coordination environments not only provide abundant accessible active sites but also enable tailored OER kinetics through the preferred coordination environments of different metal centers. In the case of M-CPs (M = Ni, Co), Ni­(II) centers can adopt a square-planar coordination geometry, while Co­(II) centers are typically stabilized in an octahedral coordination environment. When incorporating Co into Ni-CPs, their complementary geometric preference not only induces greater local lattice distortion but also promotes a synergistic interaction between Ni and Co centers. This structural modulation facilitates the formation of catalytically active high-valent M-O-M motifs, which are generally difficult to realize in conventional oxide/hydroxide-based catalysts. Consistent with this view, a recent study on MC_4_/N_4_-type catalysts has demonstrated that the dynamic evolution of well-defined local coordination geometry of metal centers into the M-O-M configurations emerges as a decisive factor for the intrinsic OER activity.^26^ Our group recently employed *operando* time-resolved spectroscopic techniques to explore the OER kinetics of a series of secondary transition metal-incorporated cobalt hydroxide-based catalysts for the OER, featuring abundant structural disorder.^27^ Our results unravelled that the generation of the OER active Co^IV^-O-Co^III/IV^ configurations derived from disordered Co-based catalysts was found to be more facile than from their crystalline counterparts. Moreover, the engineering of secondary metal (M) substitution facilitates the formation of more catalytically active di-μ-oxo bridged Co-O-M motifs, thereby offering improved OER activity. On this basis, it is expected that optimization of the local coordination geometry of active metal centers (i.e., Ni) within CPs, assisted with heteroatom (i.e., Co) substitution, exhibits great promise for generating a substantial fraction of highly active M-O-M moieties, and thereafter, activating the OPM pathway with an enhanced OER performance and durability.

Herein, a series of cobalt-substituted nickel coordination polymers (referring to Ni_1–*x*_Co_*x*_(H_2_O)_2*x*_Ni­(CN)_4_) with square-planar NiC_4_/N_4_ configurations were prepared and employed as model materials to facilitate access to the catalytically active high-valent M-O-M configurations during the OER [Note: Ni_1–*x*_Co_*x*_(H_2_O)_2*x*_Ni­(CN)_4_ is a typical cyanide-bridged coordination polymer with extended crystalline NC-Ni-CN-Ni/Co­(OH_2_)-NC motifs, distinct from conventional organic polymers]. Through combining complementary *operando* time-resolved characterization techniques, including quick-X-ray absorption spectroscopy (XAS), Raman spectroscopy, and electrochemical impedance spectroscopy (EIS), we precisely elucidated the dynamic evolution of the local coordination geometries of Ni and Co centers into catalytically active Ni^IV^-O-Co^IV^ configurations. Our *operando* results revealed that the characteristic structural features of Ni_3_Co_1_-CPs lower the energy barrier for accessing the high-valent Ni^IV^-O-Co^IV^ moieties during the OER compared to the cobalt-substituted nickel oxide (NiCo-oxide) reference with a spinel-type crystal phase. This allows the OER in Ni_3_Co_1_-CPs to proceed via an OPM pathway, with the formation of O–O bond atop the Ni^IV^-O-Co^IV^ moieties. These key findings are firmly supported by pH-dependent OER monitoring and density functional calculations (DFT). We further employed pulse chronoamperometry to quantitatively elucidate the crucial role of high-valent species accumulation in the intrinsic OER activity. Benefiting from the facilitated OER kinetics, Ni_3_Co_1_-CPs exhibit an enhanced OER activity under alkaline conditions compared to NiCo-oxide, along with robust electrochemical stability exceeding 4000 h without a significant decline.

## Results and Discussion

### Materials Synthesis and Characterization

Cobalt-incorporated nickel coordination polymers (Ni-CPs), including Ni_4_Co_1_-CPs, Ni_3_Co_1_-CPs, Ni_2_Co_1_-CPs, and NiCo-CPs, were prepared via a conventional room-temperature coprecipitation approach, followed by calcination treatment under an Ar atmosphere (see the Experimental Details and Methods section in the Supporting Information) [Note: The atomic ratio of Ni/Co was determined by energy dispersive X-ray (EDX) and inductively coupled plasma mass spectrometry (ICP-MS), as summarized in Table S1]. Powder X-ray diffraction (PXRD) analysis (Figure S1) shows that the as-prepared Ni-CPs exhibit a diffraction pattern consistent with that simulated for Ni­(CN)_2_ (Springer Materials: sd_1922727, S.G. *P*4_2_/*mmc*),^30^ exhibiting a well-defined square-planar {Ni­(CN/NC)_4_} coordination geometry. In contrast, all Co-substituted Ni-CPs display broadened diffraction features indicative of low crystallinity, with the additional peaks observed at ca. 25° assigned to the Co­(H_2_O)_2_Ni­(CN)_4_ phase (ICSD_136975, S.G. *P*12/*m*1). These observations highlight the preference of Co centers for an octahedral {Co­(NC)_4_(OH_2_)_2_} coordination geometry, indicating that partial Co incorporation induces structural distortion and phase change in Ni-CPs (detailed discussion in Figure S2). Figure 1a and Figures S3 and S4 represent the transmission electron microscopy (TEM) and field-emission scanning electron (FESEM) images of the as-prepared coordination polymer products. From the results, characteristic 2D nanoplate morphologies were observed in all synthesized materials. Compared with pristine Ni-CP, high-resolution TEM (HR-TEM) analysis shows a mixture of ordered and disordered domains in Ni_3_Co_1_-CP, corresponding to Ni_0.5_Co_0.5_(H_2_O)­Ni­(CN)_4_, further validating its low-crystalline nature (Figure 1b and Figures S5 and S6). Additionally, the observed interplanar spacings of 4.48 and 3.44 Å are assigned to the main exposed crystal planes of (100) and (110) of Ni­(CN)_2_, respectively [Note: The random bright dots observed in the HR-TEM images are attributed to the appearance of small nanoclusters (<1 nm) of Ni-CPs and Ni_3_Co_1_-CPs.]. This agrees with the observations from the corresponding selected area electron diffraction (SAED) patterns, as shown in Figures S7 and S8. Furthermore, the results of energy dispersive X-ray spectroscopy (EDX) elemental maps and line scan profiles (Figure 1c and Figures S9–S13) demonstrate a homogeneous elemental distribution of C, N, O, Ni, and Co signals across the selected areas, suggesting the successful preparation of homogeneously Co-substituted Ni-CPs.

**Figure 1:**
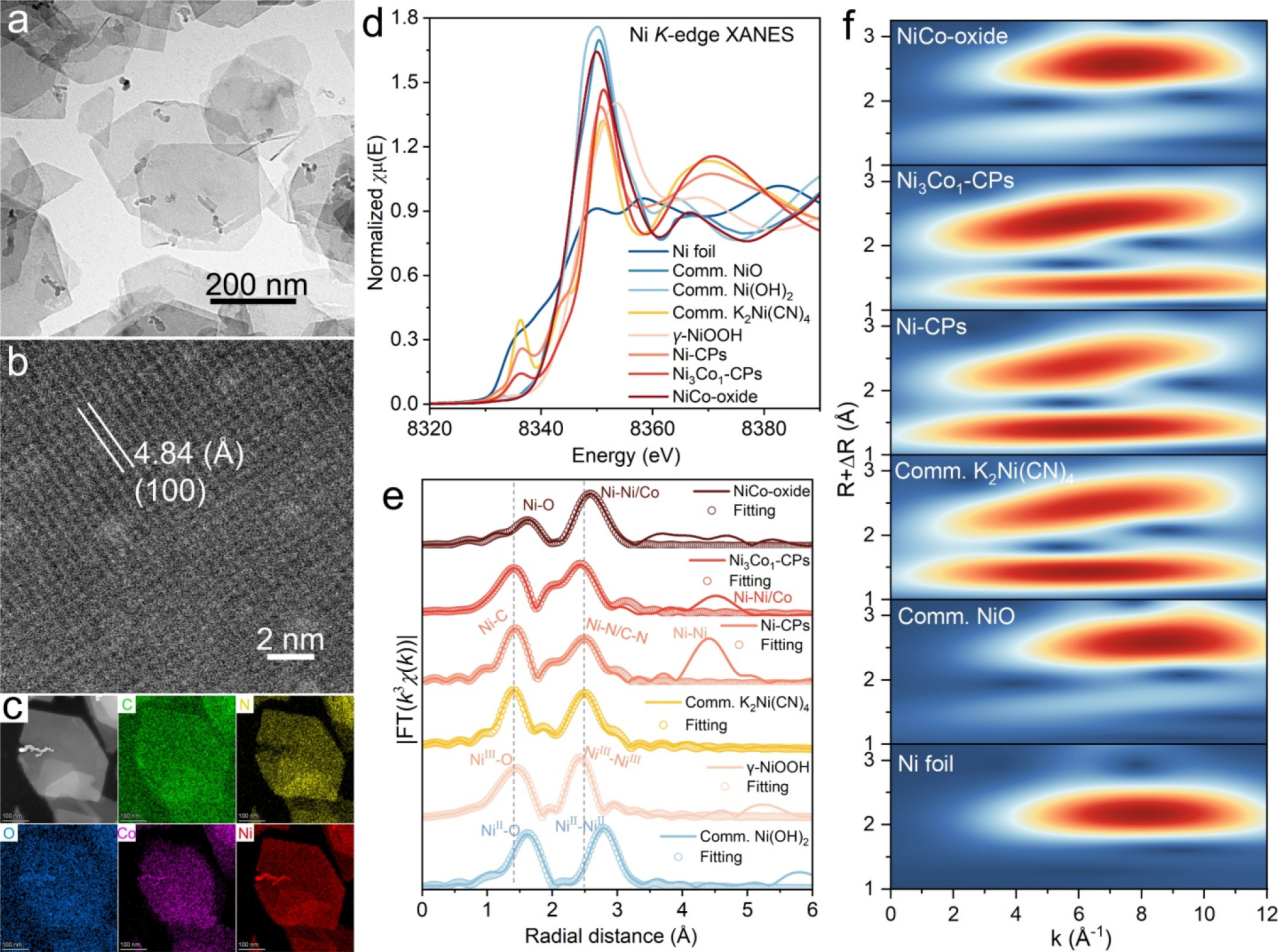
Morphological and structural characterizations of the synthesized catalysts. (a, b) Representative TEM and HR-TEM images of Ni_3_Co_1_-CPs. (c) High-angle annular dark field-scanning transmission electron microscopy (HAADF-STEM) image and STEM-EDX elemental maps of Ni_3_Co_1_-CPs. (d, e) Ni *K*-edge XANES spectra and fitting of FT-EXAFS spectra of as-prepared materials vs references. (f) WT contour profiles of Ni *K*-edge EXAFS spectra of as-prepared materials vs references (*R* = radial distance).

As a reference for the performance metrics of engineered coordination polymer catalysts for the OER, Ni_3_Co_1_-/NiCo-oxides were prepared by calcination of the as-prepared Ni_3_Co_1_-/NiCo-CPs in air (see the Experimental Details and Methods section in the Supporting Information). Structural and morphological analyses (Figures S14–S16) indicate that the resulting oxide references adopt rock salt-type (NiO, PDF no. 47-1049) and spinel-type phases (Co_3_O_4_, PDF no. 42–1467), respectively, and preserve the original nanoplate morphology. Additionally, Ni_3_Fe_1_-LDH was prepared via a urea-assisted approach and investigated as an OER performance benchmark comparison, exhibiting a typical nanosheet morphology (Figures S17 and S18).

### Analysis of the Electronic Structure and Coordination Environments

The influence of cobalt incorporation on the local electronic structure and coordination environment of Ni-CPs was investigated using X-ray absorption near-edge structure (XANES) and extended X-ray absorption fine structure (EXAFS) spectra, alongside comparative analysis of the NiCo-oxide reference (Figure 1d–f and Figures S19 and S20). From the Ni *K*-edge XANES spectra (Figure 1d), the spectral profile of Ni-CPs closely resembles that of the K_2_Ni­(CN)_4_ reference, featuring a pronounced pre-edge peak at ca. 8336.5 eV (assigned to the Ni 1s to 3d transitions) and a weaker shoulder peak at ca. 8344.5 eV (attributed to the Ni 1s to Rydberg transitions).^29,31–33^ This indicates that the Ni centers in Ni-CPs adopt a square-planar Ni­(II)­X_4_ (X = C, N) geometry, analogous to that in reference K_2_Ni­(CN)_4_. For the Co-incorporated Ni-CPs, comparable square-planar Ni­(II)­X_4_ features are identified from their Ni *K*-edge XANES spectra (Figure S19a). However, a close inspection notably unravels a spectral profile change of the Ni­(II)­X_4_ features, implying that partial Co substitution induces local structural distortion around the Ni centers. Analysis of the Co *K*-edge XANES spectra (Figures S19d and S20a) demonstrates that the average Co valence state is predominantly +2 in all Co-substituted Ni-CPs. Note that no distinct spectral signatures associated with a square-planar Co­(II)­X_4_ geometry are observed. This is mainly attributed to the coordination preference of Co­(II) centers for both [Ni­(CN)_4_]^2–^ complex anions and crystalline water molecules, leading to the formation of an octahedral {Co­(NC)_4_(OH_2_)_2_} coordination geometry, consistent with our above PXRD analysis (detailed discussion in Figure S2). Consequently, only quadrupole-allowed Co 1s to 3d transitions occur, contributing to the diminished spectral signal intensity of the square-planar features.^31,34^

The local coordination environments of Ni and Co centers in the investigated catalysts were further explored through Fourier transform (FT) EXAFS spectra. From the Ni *K*-edge FT-EXAFS spectra (Figure 1e and Figure S19c and Table S2), all Co-substituted Ni-CP samples exhibit two prominent backscattering peaks within the *R* + Δ*R*= 1 to 3 Å compared to those of Ni-CPs and K_2_Ni­(CN)_4_ reference. These features are assigned to backscattering contributions from the Ni–C and NiN/–C–N paths. Fitting of the Ni *K*-edge EXAFS spectra (Table S2) indicates coordination numbers of 4.00 for both the Ni–C and Ni–N paths, and 8.00 for the Ni–C–N path in all CP samples, thereby validating the preservation of a square-planar Ni­(II)­C_4_/N_4_ geometry. Analogous phenomena are also evident from wavelet transform (WT) analysis of the Ni *K*-edge EXAFS spectra. As depicted in Figure 1f, two intensity maxima within the *k* range of 6 to 7 Å^–1^, attributed to the Ni–C and Ni–N/–C–N scattering contributions, are consistently observed in Ni-CPs, Ni_3_Co_1_-CPs, and the K_2_Ni­(CN)_4_ reference. Notably, Ni-CPs feature a backscattering signal at *R* + Δ*R* of ca. 4.41 Å, which can be assigned to the backscattering of Ni–Ni pairs within Ni–CN–Ni moieties. In contrast, this signal shifts to a longer distance in Ni_3_Co_1_-CPs (ca. 4.52 Å), reflecting the presence of Ni–Co backscattering within Ni-CN-Co­(OH_2_) moieties. Investigations of the Co *K*-edge FT-EXAFS spectra provide additional insights into the local coordination environment of the Co centers. From the results (Figures S19f and S20 and Table S3), a 6-fold coordination environment is evident for the first Co coordination shells in all CP samples. Specifically, the Co­(II) centers are coordinated by 4 N atoms from [Ni­(CN)_4_]^2–^ complex anions and 2 O atoms from crystalline water molecules, forming a distorted octahedral geometry. This aligns well with the above investigations on the Co *K*-edge XANES spectra, where characteristic Co­(II)­X_4_ features are barely observed. It should also be mentioned that Co­(H_2_O)_2_Ni­(CN)_4_ (referred to as NiCo-CPs) only involves the characteristic Ni-CN-Co­(OH_2_) moieties, as confirmed by an identical long-range backscattering signal at *R* + Δ*R* of ca. 4.54 Å from both Ni and Co *K*-edge FT-EXAFS spectra (Figure S19c,f). For comparison, the local electronic structure and coordination environments of metal centers in the NiCo-oxide reference were also analyzed by XAS characterizations, and the results are consistent with a spinel-type crystal structure, as shown in Figure 1d–f and Figure S20 **.**

### Electrochemical OER Characterization

We assessed the electrocatalytic OER performance of the as-prepared CP catalysts under alkaline conditions, along with the oxide reference and the state-of-the-art Ni_3_Fe_1_-LDH (Figure 2 and Figures S21–S28). Figure 2a presents the recorded CV curves of the catalysts toward the OER. Note that the currents were normalized by electrochemically active surface area (ECSA) to assess the intrinsic catalytic activities.^2,35–37^ From the results (Figure 2a and Figure S22), to achieve a current density of 1 mA/cm_ECSA_^2^, a minimum overpotential of 317 mV is required for the as-prepared Ni-CPs. Upon Co substitution, the overpotentials are substantially reduced to 285 and 242 mV for Ni_4_Co_1_-CPs and Ni_3_Co_1_-CPs, respectively. However, higher Co/Ni ratios compared with Ni_3_Co_1_-CPs result in an increase of the overpotentials to 306 and 349 mV for Ni_2_Co_1_-CPs and NiCo-CPs, respectively. Figure 2b summarizes the overpotentials at 10 mA/cm_geo._^2^ and the mass activities at 1.53 V vs RHE for all investigated catalysts. As seen, Ni_3_Co_1_-CPs display the lowest overpotential of 239 mV and the highest mass activity of 559.2 A/g, comparable to both the other CP catalysts and the NiCo-oxide reference, as well as the state-of-the-art OER catalysts of Ni_3_Fe_1_-LDH (Figures S22 and S23). Tafel plots and EIS measurements (Figure 2c and Figure S22d) were further conducted to gain in-depth insights into the intrinsic OER kinetics. Among the investigated catalysts, Ni_3_Co_1_-CPs feature the most favorable OER kinetics, with a lower Tafel slope value of 32.82 mV/dec and a smaller transfer resistance of 7.1 Ω. This highlights the beneficial impact of partial Co incorporation on facilitating the OER process in Ni-CPs. Furthermore, rotating-ring disk electrode (RRDE) experiments (Figure 2d) unveil nearly 100% Faradaic efficiency for Ni_3_Co_1_-CPs, verifying that the observed electrocatalytic activity is exclusively derived from oxygen evolution.

**Figure 2:**
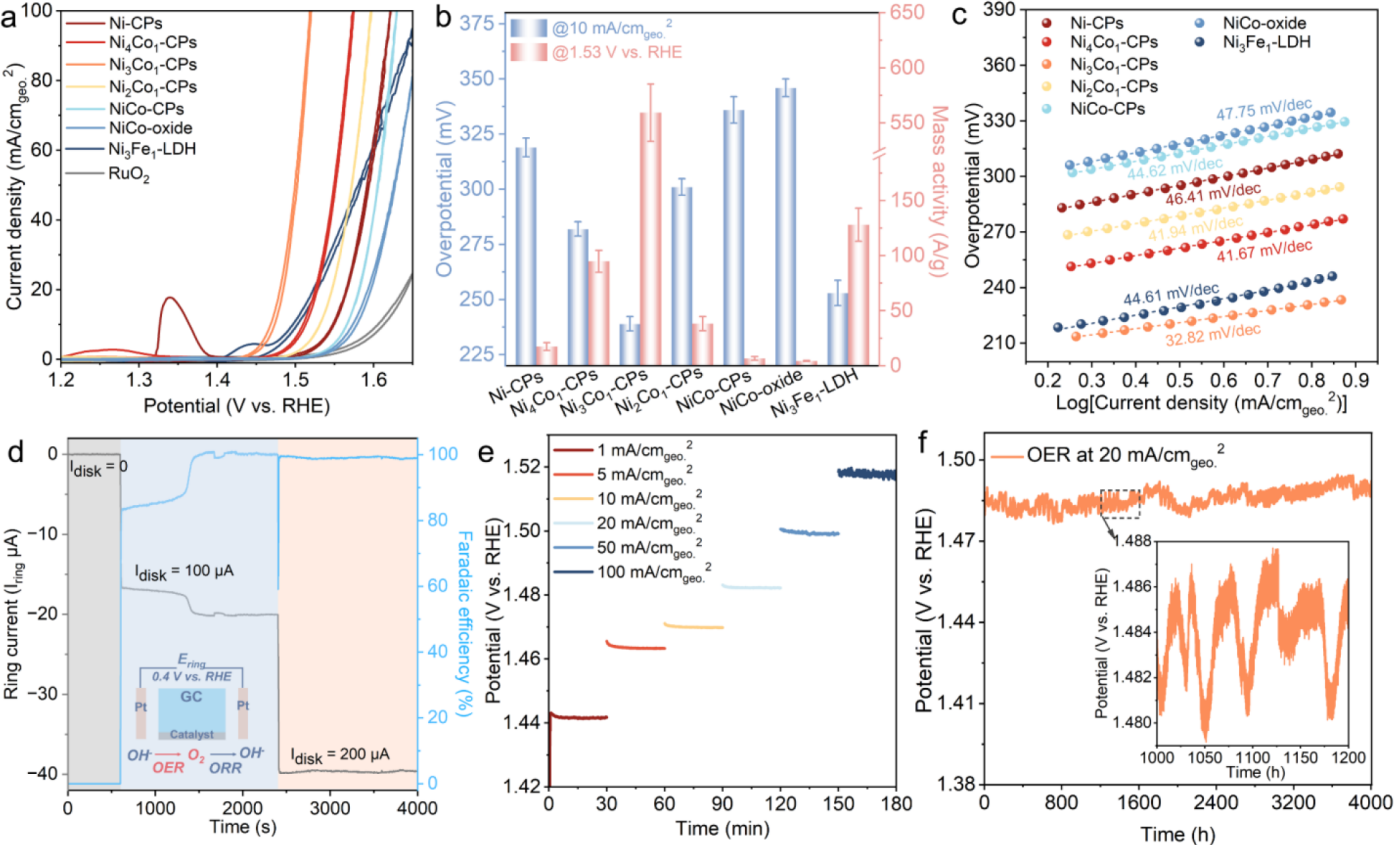
Electrocatalytic OER characterizations of the investigated catalysts in 1 M KOH (pH ∼13.7). (a) Normalized cyclic voltammetry (CV) curves with a 90% *iR* drop correction (currents were normalized by ECSA). (b) Comparison of overpotentials at 10 mA/cm_geo._^2^ and mass activities at 1.53 V vs RHE. (c) Tafel plots. (d) Faradaic efficiency of Ni_3_Co_1_-CPs tested in Ar-saturated 1 M KOH based on RRDE techniques. (e) Chronopotentiometry measurements of Ni_3_Co_1_-CPs at different current densities. (f) Long-time stability measurements of Ni_3_Co_1_-CPs loaded on carbon paper.

The electrochemical stability of Ni_3_Co_1_-CPs catalysts was first examined by a multistep chronopotentiometry test at varying current densities (Figure 2e). Subsequently, the investigated catalysts were loaded on carbon paper for long-term measurements (Figure 2f and Figures S26 and S27). Our results demonstrate a robust OER performance at 20 mA/cm_geo._^2^ for over 4000 h and 200 mA/cm_geo_.^2^ for over 400 h, respectively, with a negligible change in the most active Ni_3_Co_1_-CPs. Such a durable OER activity obtained in Co-substituted Ni-CPs also renders them competitive with recently reported high-performance OER electrocatalysts (Table S4). In addition, the synthesized Ni_3_Co_1_-CPs were further evaluated for the electrocatalytic oxidation of small organic molecules, including ethylene glycol (EG), 5-hydroxymethylfurfural (HMF), and urea. Our results (Figure S28) also reveal that Ni_3_Co_1_-CPs emerge as an alternative candidate to facilitate the electrochemical conversion process of these small molecules.

### Post-catalytic OER Characterizations

To shed more light on the origin of the robust OER durability of the as-prepared Co-incorporated Ni-CPs, post-catalytic characterizations, such as PXRD, FESEM, ICP-MS, X-ray photoelectron spectroscopy (XPS), and XAS, were carried out (Figure 3 and Figures S29–S38 and Table S5) [Note: In the following discussion, “post-catalytic” refers to the catalysts after the OER stability measurements shown in Figure 2f and Figure S26]. PXRD analysis of the investigated CP catalysts (Figure S29a–c) reveals that all characteristic diffraction features associated with the pristine materials completely disappeared after the OER. Newly appearing diffraction peaks at ca. 20°, 39°, and 65° are ascribed to the presence of NiOOH/CoOOH species. In comparison, the NiCo-oxide reference retained its structural integrity well before and after the OER (Figure S29d). Results from the post-catalytic FESEM-EDX characterizations of Ni_3_Co_1_-CPs, corresponding to Ni_0.5_Co_0.5_(H_2_O)­Ni­(CN)_4_, show a decrease in the atomic Ni/Co ratio from 3.05:1 (pristine) to 1:1 (after the OER) (Figures S30 and S31 and Table S1). Notably, for the post-catalytic EDX spectrum of NiCo-CPs, corresponding to Co­(H_2_O)_2_Ni­(CN)_4_, no discernible Ni signals were detected. This implies that the OER in the investigated Co-incorporated Ni-CPs induced distinct structural rearrangement, accompanied by [Ni­(CN)_4_]^2–^ leaching (Table S5). Examination of the N 1s, Co 2p, and Ni 2p XP spectra further clarify the following surface transformations after the OER (Figure S32): (*i*) All CP catalysts show a complete loss of N 1s signals after the OER; (*ii*) The Co 2p XP spectra of Ni_0.5_Co_0.5_(H_2_O)­Ni­(CN)_4_ (Ni_3_Co_1_-CPs) and Co­(H_2_O)_2_Ni­(CN)_4_ (NiCo-CPs) exhibit a negative shift toward lower binding energy and the appearance of satellite features at higher binding energy, indicating that high-valent Co oxide/hydroxide species appear on the surface after the OER;^27,38,39^ (*iii*) In the Ni 2p region, a positive binding energy shift along with newly appearing satellite peaks is evident from Ni­(CN)_2_ (Ni-CPs) and Ni_0.5_Co_0.5_(H_2_O)­Ni­(CN)_4_ (Ni_3_Co_1_-CPs) after the OER, reflecting that oxidized Ni species (e.g., oxide/hydroxide) dominate the surface of the investigated CP catalysts.^38,40^ Importantly, no significant Ni-related XPS features are present on the surface of the post-catalytic NiCo-CPs, in agreement with the above PXRD and EDX analysis, further verifying that restructuring of CP catalysts proceeds with an underlying leaching of [Ni­(CN)_4_]^2–^ complex anions into the electrolyte (Table S5).

**Figure 3:**
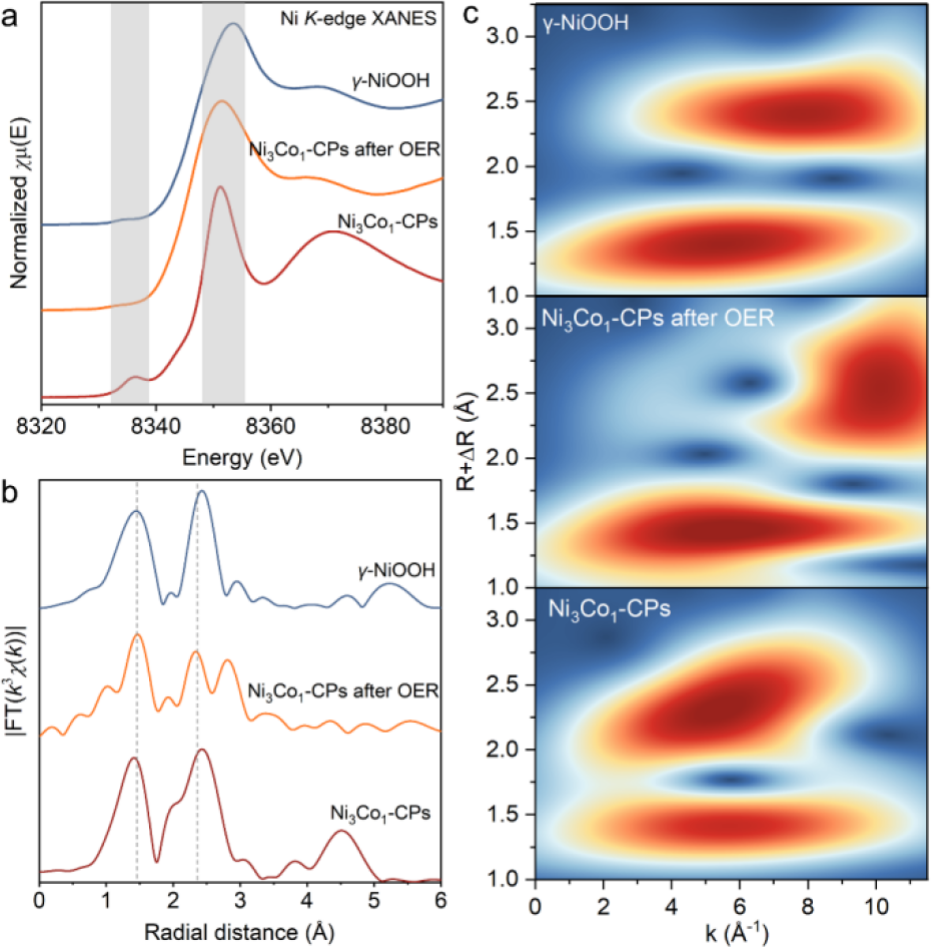
XAS characterizations of Ni_3_Co_1_-CPs before and after long-time OER stability measurements. (a, b) Ni *K*-edge XANES and FT-EXAFS spectra. (c) WT contour profiles of Ni *K*-edge EXAFS spectra.

Post-catalytic Ni and Co *K*-edge XAS characterizations were carried out to gain an in-depth insight into the changes in the local electronic structure and coordination environments of the catalysts after the OER. From the results (Figure 3 and Figures S33–S38), analogous evidence for the appearance of significant structural transformations in CP catalysts after the OER was established. Taking Ni_3_Co_1_-CPs as an example, the post-catalytic Ni and Co *K*-edge XANES spectra (Figure 3a and Figure S35a) exhibit spectral profiles closely resembling those of γ-NiOOH and CoOOH references, respectively. Interpretations of the Ni and Co *K*-edge EXAFS spectra manifest that Ni_3_Co_1_-CPs underwent restructuring into NiCo oxyhydroxide-like species during the OER (Figure 3b,c and Figure S35b,c). Similar restructurings are also observed in Ni-CPs and NiCo-CPs, as supported by their post-catalytic XAS characterizations (Figures S33, S35, and S37). In sharp contrast, analysis of the Ni and Co *K*-edge XAENS spectra of the NiCo-oxide reference only features slight positive energy shifts in the Ni and Co *K*-edge positions (*F*/*I*_0_ = 0.5), reflecting the generation of a quite low fraction of high-valent species (Figure S38). However, the results of Ni and Co *K*-edge EXAFS and FT-EXAFS spectra of NiCo-oxide clearly evidenced well-preserved structural integrity before and after the OER, pointing out that their restructuring into high-valent species derived from oxide-type materials is a kinetically demanding process compared to the CPs. To further elucidate the underlying OER kinetics in CP- and oxide-based catalysts and to unravel the crucial role of Co incorporation in their structural dynamics, *operando* monitoring of the catalysts during the reaction process was performed (cf. following section).

### Dynamics of Active Species and Sites

The dynamic evolution of the oxidation state and local coordination environments of Ni and Co centers in the investigated catalysts during the OER was explored using *operando* time-resolved quick-XAS. We first performed *operando* Ni *K*-edge XAS characterizations for Ni-CPs to monitor the dynamics of active species and sites under the OER conditions (Figure S39 and Table S6). Our results (detailed discussion in Figures S39 and S40) demonstrate that Ni-CPs undergo a multistep restructuring into high-valent Ni­(IV) species that serve as the true OER active species. The role of Co incorporation in the structural evolution and the OER kinetics was further examined through *operando* time-resolved quick-XAS characterizations in Ni_3_Co_1_-CPs. Figure 4a,e depicts the *operando* time-resolved Ni and Co *K*-edge XANES monitoring results for Ni_3_Co_1_-CPs. With the anodic polarization, the white line signatures at ca. 8351.5 (for Ni *K*-edge) and 7725.5 eV (for Co *K*-edge), corresponding to the dipolar transitions from Ni and Co 1s to 4p hybridized orbitals,^33,35^ respectively, feature remarkable spectral profile changes. In detail (Figure 4b,f), after immersing into the electrolyte, the signal intensity of the Ni *K*-edge white line signature increases slightly, while its peak position remains almost identical. Conversely, the Co *K*-edge white line profile behaves oppositely, namely a decrease in the peak intensity accomplished by a positive energy shift from 7725.5 to 7727.3 eV. Such distinct changes that occurred on the Ni and Co *K*-edge white line profiles are ascribed to an anion exchange reaction between [Ni­(CN)_4_]^2–^ and OH^–^, as corroborated by our *operando* XAS analysis for Ni-CPs (Figures S39 and S40). Importantly, this anion exchange reaction preferentially occurs at the Co center, as evidenced by the pronounced spectral profile changes observed from the *operando* Co *K*-edge XANES compared to that of Ni centers (Figure 4b,f). With the applied potential increased from 0.9 to 1.4 V vs RHE, negligible profile changes can be detected on the Ni *K*-edge white line features; however, significant spectral profile changes toward lower peak intensities and higher energy positions are found from 1.4 to 1.5 V vs RHE. Analysis of the Co *K*-edge white line signatures exhibits a spectral profile change starting from a lower potential of 1.35 V vs RHE compared to that of the Ni *K*-edge (1.4 V vs RHE), implying that during the OER the *OH intermediate adsorption is thermodynamically more favorable at the Co sites than the Ni sites. Removing the applied potentials results in an enhanced peak intensity along with a negative energy shift in peak position for both the Ni and Co *K*-edge white line signatures. This manifests that Ni_3_Co_1_-CPs undergo local coordination geometry evolution at both the Ni and Co centers, leading to the formation of more OER active moieties for the O_2_ release. Notably, these *in situ*-generated active moieties can reversibly convert into the OER resting species after the reaction. Investigations of the Ni and Co *K*-edge positions can deliver in-depth insights into the valence state dynamics of metal centers during the OER process.^2,41^ The results (Figure 4c) show that the Ni *K*-edge position of Ni_3_Co_1_-CPs shifts toward higher energy as a function of the applied potential and reaches its maximum value of ca. 8345.6 eV at 1.5 V vs RHE, which is obviously higher than those of γ-NiOOH (8344.9 eV) and Ni-CPs (8343.9 eV at 1.52 V vs RHE, Figure S39b). This key finding indicates that partial Co incorporation facilitates the generation of high-valent Ni­(IV) species that account for the OER in Ni_3_Co_1_-CPs. Note that analysis of the Co *K*-edge position of Ni_3_Co_1_-CPs unravels a maximum value of ca. 7722.5 eV at 1.475 V vs RHE, higher than that of reference CoOOH (7721.3 eV), but thereafter, it remains almost identical upon increasing the applied potential to 1.5 V vs RHE (Figure 4g). This is a piece of evidence to corroborate that during the OER the Ni sites in Ni_3_Co_1_-CPs exhibit a lower energy barrier for the deprotonation of Ni­(III)-OH into Ni­(IV)-O intermediates when compared to the Co sites.

**Figure 4:**
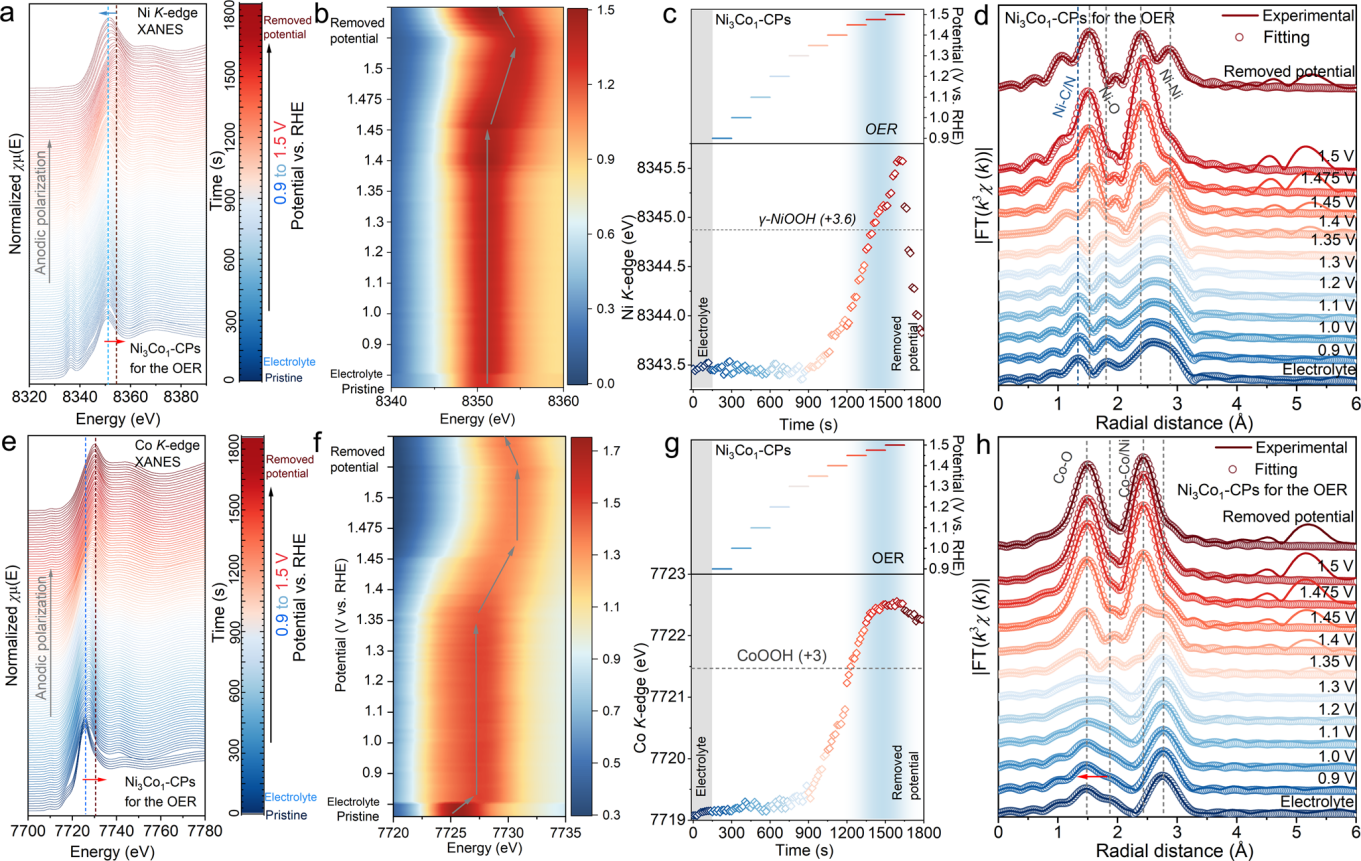
*Operando* time-resolved quick-XAS characterizations of Ni_3_Co_1_-CPs for the OER under alkaline conditions. (a, e) Ni and Co *K*-edge XANES spectra. (b, f) 2D contour plots of Ni and Co *K*-edge XANES spectra. (c, g) Ni and Co *K*-edge positions (*F*/*I*_0_ = 0.5) against the OER reaction time and applied potentials. (d, h) Fitting of *operando* Ni and Co *K*-edge FT-EXAFS spectra. (Note: “Removed potential” means stopping applying any potentials, but not exposure to air.)

To unravel the dynamic influence of Co substitution on the local coordination environments of Ni centers in Ni_3_Co_1_-CPs, *operando* time-resolved Ni and Co *K*-edge EXAFS and FT-EXAFS spectra were further recorded (Figure 4d,h and Figure S41). Consistent with our *operando* investigations on Ni-CPs (detailed discussion in Figures S39 and S40), Ni_3_Co_1_-CPs also show time-dependent changes in the local coordination environments of both Ni and Co centers during the reaction process, with the generation of high-valent (Ni, Co)­(IV) active species for triggering the OER (detailed discussion in Figures S41–S44). Specifically, for catalysts immersed into the electrolyte, the Ni and Co *K*-edge FT-EXAFS spectra of Ni_3_Co_1_-CPs display characteristic backscattering signals corresponding to Ni–O and Co–O paths within the interval *R* + Δ*R* = 1.5 to 2 Å (Figure 4d,h and Tables S7 and S8). Besides, the newly observed second shell scattering peaks in the region of 2.5 to 3 Å are attributed to the appearance of Ni­(Co)-O-Ni/Co configurations. Notably, our results (Tables S7 and S8) indicate that CN_Co–O_ is approximately twice CN_Ni–O_, and a similar trend is also observed in the comparison between CN_Ni–Ni/Co_ and CN_Co–Co/Ni_. These disparities point out that during the pre-activation process, the underlying local coordination geometry optimization in Ni_0.5_Co_0.5_(H_2_O)­Ni­(CN)_4_ (Ni_3_Co_1_-CPs), driven by the OH^–^ interactions, proceeds more easily at the Co centers than the Ni centers. This is also in line with our above *operando* XANES spectra studies (Figure 4b,f), where the Co *K*-edge white line signatures undergo more distinct profile changes during the preactivation process relative to the Ni *K*-edge. Upon anodic polarization from 1.4 (1.35) V to 1.52 V vs RHE, the newly formed Ni­(Co)-O and Ni­(Co)-Ni/Co scattering paths shift toward shorter interatomic distances, inferring the generation of catalytically active Ni­(Co)^IV^-O-Ni^III/IV^/Co^III/IV^ moieties. This observation about the formation of high-valent OER active moieties is aligned with our previous discussions on the Ni and Co *K*-edge positions (Figure 4c,g), which demonstrate the appearance of a considerable fraction of Ni^IV^ and Co^IV^ species in the OER region.

To further elucidate the pivotal role of synergistic interactions between Ni and Co centers in facilitating the formation of high-valent OER active species, *operando* XAS characterizations of Co­(H_2_O)_2_Ni­(CN)_4_ (NiCo-CPs) and reference NiCo-oxide were carried out (Figures S45–S47). The o*perando* Ni *K*-edge XAS investigations of NiCo-CPs (Figure S45) demonstrate a distinct signal intensity drop in the absorption edge step for catalysts immersed into the electrolyte. Additionally, no discernible Ni *K*-edge XAS signals are observed in NiCo-CPs after the OER. This again reinforces the occurrence of an underlying exchange reaction between [Ni­(CN)_4_]^2–^ and OH^–^, which results in Ni leaching into the electrolyte (see the Post-Catalytic Characterizations section and Table S5 in the Supporting Information). In agreement with our *operando* XAS monitoring for Ni_3_Co_1_-CPs, analogous evolution in the local coordination geometry and oxidation state of Co centers is also found in NiCo-CPs during the OER (Figure S46). In detail, with the anodic polarizations, the Co *K*-edge position features a positive energy shift as the applied potentials increase, and reaches its maximum value of 7721.6 eV at 1.55 V vs RHE (CoOOH: 7721.3 eV). Correspondingly, two dominant scattering features newly appear at *R* + Δ*R* = 1.5 and 2.4 Å in the *operando* Co *K*-edge FT-EXAFS spectra, associated with the backscattering contributions from the Co^III/IV^-O and Co^III/IV^-O-Co^III/IV^ paths, respectively. As we repeatedly discussed above, the Co *K*-edge positions in Ni_3_Co_1_-CPs feature a maximum value of 7722.5 eV at 1.5 V vs RHE, which is distinctly higher than that of NiCo-CPs (7721.6 eV at 1.55 V vs RHE). These results corroborate that the generation of high-valent OER active species in NiCo-CPs is a kinetically demanding process in the absence of synergistic interactions between Ni and Co centers. Concerning the OER in NiCo-oxide, *operando* XAS analyses (detailed discussion in Figure S47) demonstrate that the construction of catalytically active Ni^IV^-O-Co^III/IV^ configurations is found to be much more difficult compared to Ni_3_Co_1_-CPs, highlighting the advantages of engineered coordination polymer catalysts for promoting the OER performance.

### Role of High-Valent Species in the OER

To obtain detailed insight into the crucial role of *in situ*-generated high-valent species in the OER of the investigated catalysts, *operando* Raman characterizations were further conducted (Figure 5a and Figures S48 and S49). As shown in Figure 5a, Ni_3_Co_1_-CPs initially feature two Raman fingerprints at 455 and 535 cm^–1^ at 0.9 V vs RHE, corresponding to the Ni^II^/Co^II^–O vibrations;^12,42–44^ upon anodic polarizations higher than 0.9 V vs RHE, these *in situ*-generated Raman signatures change their profile toward higher signal intensities and larger wavenumbers, appearing at 478 and 558 cm^–1^ in the OER region (Figure 5a and Figure S49), associated with the (Ni, Co)^III/IV^-O vibrations;^11,12,44–46^ when the applied potential is reversed to 1.0 V RHE, these high-valent species vibrations revert toward smaller wavenumbers, reflecting the dynamic and reversible evolution of the *in situ*-generated high-valent catalytically active species into the OER resting species after the reaction.

**Figure 5:**
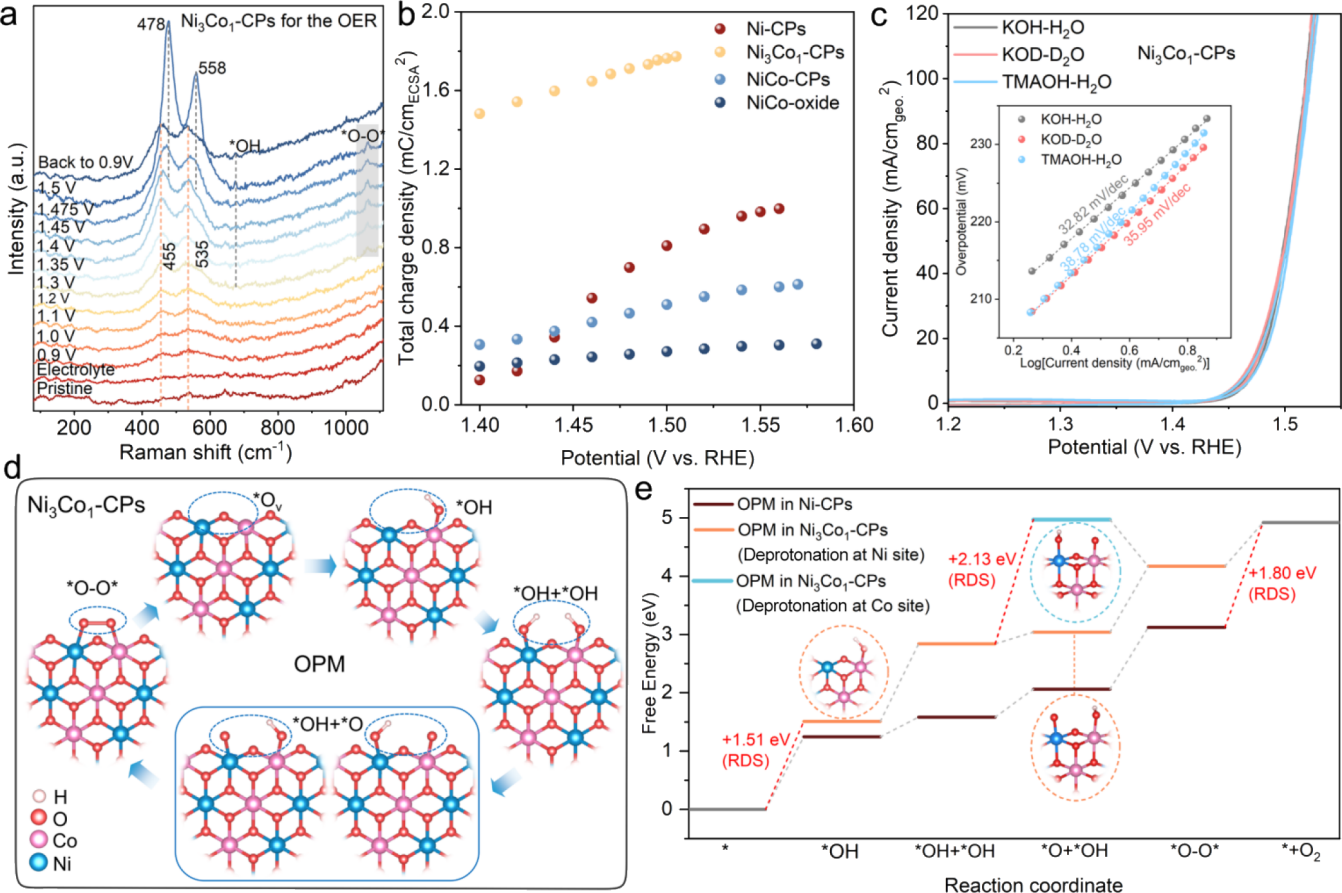
OER kinetics investigations and DFT calculations. (a) *Operando* Raman spectra of Ni_3_Co_1_-CPs for the OER. (b) Calculated surface accumulated charge density of the investigated four catalysts. (c) CV curves of Ni_3_Co_1_-CPs for the OER in 1 M KOH in H_2_O, 1 M KOD in D_2_O, and 1 M TMAOH in H_2_O. (d) Schematic illustration of the OER cycling process via an OPM pathway in Ni_3_Co_1_-CPs. (e) Calculated free energy diagrams of OER intermediates adsorbed on Ni-/Ni_3_Co_1_-CPs via an OPM pathway.

*Operando* Raman analyses of Ni-CPs, NiCo-CPs, and NiCo-oxide further corroborate the emergence of dynamic surface evolution during the OER. For instance, Ni-CPs exhibit two newly emergent Raman features at ca. 481 and 562 cm^–1^ under the OER-relevant potentials (Figure S48a), which originate from the Ni^III/IV^-O vibrations from γ-NiOOH.^44,46–48^ Similarly, two prominent Raman fingerprints at ca. 463 and 566 cm^–1^, assigned to the Co^III/IV^-O vibrations, are observed in NiCo-CPs during the OER (Figure S48b). Note that these newly appearing Raman signatures in Ni-CPs and NiCo-CPs during the OER feature distinct wavenumber shifts compared to those of Ni_3_Co_1_-CPs. Moreover, such vibrational differences can not be explained by Ni- or Co-only contributions but rather from strong synergistic interactions between Ni and Co centers. This suggests that partial Co substitution in Ni-CPs promotes the formation of high-valent Ni^IV^-O-Co^IV^ moieties that act as the true catalytically active species for the OER. Concerning the OER in NiCo-oxide, our results reveal comparable surface kinetics as Ni_3_Co_1_-CPs, which exhibit two expected Raman signatures at 479 and 558 cm^–1^ at 1.55 V vs RHE (Figure S48c), corresponding to the (Ni, Co)^III/IV^-O vibrations. It is also noteworthy that the persistent Raman signals at 675 cm^–1^ in NiCo-oxide, attributed to the Co^II^–O vibrations within octahedral geometry, remain largely unchanged throughout the entire OER monitoring. This suggests that oxide-type catalysts only undergo partial surface restructuring into high-valent active species during the OER process, in agreement with our previous XAS discussions.

Such a key observation about the generation of high-valent Ni^IV^/Co^IV^ species during the OER is also supported by our *operando* EIS measurements. From the results (Figure S50), the Bode plots of all CP catalysts exhibit two distinct electrochemical processes, namely, a low-frequency region (<10 Hz) associated with the OER process and a high-frequency region for the redox reaction process (10^2^–10^3^ Hz).^49,50^ Within the high-frequency region, two-step oxidation processes are involved: the initial oxidation of (Ni, Co)­(II) to (Ni, Co)­(III) species below 1.2 V vs RHE, followed by the generation of (Ni, Co)­(IV) species at the OER-relevant potentials. Compared to other CP catalysts, the high-frequency region in Ni_3_Co_1_-CPs exhibits considerable redox oxidation features at the OER-relevant potentials, highlighting the beneficial role of partial Co incorporation in promoting the OER kinetics. In contrast, no discernible redox features are observed in the Bode plots of NiCo-oxide (Figure S50h), further emphasizing that the oxide-type catalysts generally experience a higher energy barrier for the generation of high-valent active species during the OER.

As we emphasized in the above section, the formation of high-valent active species is a fundamental prerequisite for initiating the OER of the investigated catalysts. On this basis, we further conducted pulse chronoamperometry measurements (Figures S51 and S52) to quantitatively identify the intrinsic correlation between the accumulated high-valent active species and the observed OER activities.^1,27,51,52^ As illustrated in Figure 5b, the accumulated total charge densities of four catalysts display an increasing tendency during anodic polarizations, with the values in the OER region (>1.5 V vs RHE) following the order: Ni_3_Co_1_-CPs > Ni-CPs > NiCo-CPs > NiCo-oxide. These key findings are largely consistent with the above electrocatalytic characterizations of the investigated catalysts for the OER, pointing out the critical role of the accumulation of high-valent active species in promoting the OER activity.

### Investigation of the Intrinsic OER Mechanism

DFT calculations were further employed to elucidate the underlying OER mechanism of the as-prepared Co-substituted Ni-CPs, with particular emphasis on the impact of the synergistic interactions between Ni and Co centers on the intrinsic catalytic activity. Figure S53 provides pH-dependent OER studies on the investigated catalysts, and the results clearly suggest enhanced OER activity from pH 12.6 to 13.8. Moreover, the proton reaction order was determined as 1.57 for Ni-CPs, 1.85 for Ni_3_Co_1_-CPs, and 1.57 for NiCo-oxide, respectively, indicating that the OER of the catalysts involves a non-concerted proton–electron transfer (PT/ET) mechanism. When the OER was evaluated in 1 M tetramethylammonium hydroxide (TMAOH) (Figure 5c and Figure S54), all three catalysts exhibited OER activities comparable to those in 1 M KOH. This excludes the involvement of lattice oxygen activation during the OER. To further identify the reaction pathway, the investigated catalysts were characterized for the OER in 1 M KOD dissolved in D_2_O. The results (Figure 5c and Figure S54) indicate a negligible kinetic isotopic effect (KIE), concluding that the rate-determining step (RDS) does not involve the cleavage of the O–H bond. *Operando* Raman studies (Figure 5a and Figure S48) demonstrate a newly appearing vibration at ca. 1065 cm^–1^, which corresponds to the *O–O* intermediate vibrations.^12,13,50,53–55^ Taking into account all information from our electrochemical and spectroscopic investigations, we propose that the OER in Ni_3_Co_1_-CPs proceeds via an OPM pathway, in which the O–O bond forms atop the *in situ* generated catalytically active Ni^IV^-O-Co^IV^ moieties.

Figure 5d and Figure S55 illustrate the OER cycling process with the proposed OPM in Ni-CPs and Ni_3_Co_1_-CPs. For the Ni_3_Co_1_-CPs, the following pathways are plausible prior to O_2_ generation: (1) the formation of dual *OH intermediates involves competitive adsorption at the Ni and Co sites; (2) the deprotonation process can proceed either at the Ni sites to generate Ni­(IV)-O + Co­(III)-OH intermediates, or at Co sites for the formation of Ni­(III)-OH + Co­(IV)-O intermediates. Gibbs free energy calculations (Figure 5e and Figure S56) suggest that the Co sites preferentially adsorb the *OH intermediates, while the Ni sites exhibit a lower energy barrier for the deprotonation of Ni­(III)-OH into Ni­(IV)-O intermediates. These insights align well with our above *operando* XAS monitoring (Figure 4). Furthermore, the RDS energy barrier for the OPM pathway in Ni_3_Co_1_-CPs is calculated as 1.51 eV, significantly lower than that of Ni-CPs (1.80 eV), demonstrating the benefits of engineered partial Co substitution in Ni-CPs to facilitate the OER kinetics. For a better comparison, we also calculated the Gibbs free energies for the adsorption of OER intermediates in the AEM pathway. Our results (Figures S57 and S58) show that the RDS energy barriers for the AEM pathway in Ni-CPs and Ni_3_Co_1_-CPs are 2.00 and 1.77 eV, respectively, both higher than those calculated for the OPM pathway. These results substantiate that the OER in Ni_3_Co_1_-CPs is thermodynamically more favorable via an OPM pathway, which is also consistent with projected density of states (PDOS) analysis (detailed discussion in Figures S59 and S60).

## Conclusions

In summary, we introduce a facile cobalt substitution strategy to synthesize a series of mixed nickel-based coordination polymers (referring to Ni_1–*x*_Co_*x*_(H_2_O)_2*x*_Ni­(CN)_4_) as electrocatalysts for the OER. The dynamic interactions between Ni and Co centers during the reaction process render the as-prepared Ni_3_Co_1_-CP with optimized Ni/Co ratio an active and robust electrocatalyst toward the OER, maintaining current densities of 20 mA/cm_geo._^2^ over 4000 h without a significant decline.

By combining complementary surface/bulk sensitive *operando* time-resolved spectroscopic techniques, we reveal that Ni_3_Co_1_-CPs undergo an electrochemically driven restructuring into the catalytically active high-valent Ni^IV^-O-Co^IV^ motifs during the OER. Notably, removal of the applied potential induces a reversible transformation of these active moieties back to the OER resting state that is enriched in Ni^II/III^-O-Co^II/III^ configurations. In contrast, analogous *operando* spectroscopic investigations on the spinel-type mixed (Ni, Co)-oxide reference demonstrated that the formation of key Ni^IV^-O-Co^IV^ moieties was hindered, highlighting the beneficial influence of engineered CP catalysts on promoting the OER kinetics.

To unveil the origin of the observed OER activity and to elucidate the crucial role of Co incorporation in the structural dynamics, pulse chronoamperometry characterizations were further conducted to quantitatively identify the accumulated high-valent (Ni, Co)­(IV) species during the OER. The results indicate that partial Co substitution in Ni-CPs enables a high fraction of high-valent active species during the anodic polarizations, thereby facilitating the OER kinetics of Ni_3_Co_1_-CPs. Furthermore, combination of *operando* Raman, pH-dependent, and KIE studies corroborates that the generation of *O–O* intermediates atop the Ni^IV^-O-Co^IV^ moieties in Ni_3_Co_1_-CPs is the key to triggering the OER. This also manifests that the OER in the synthesized Ni_3_Co_1_-CPs proceeds via an oxo-radical coupling pathway (OPM), which involves the adsorption/desorption of OER intermediates at both Ni and Co sites. This mechanistic model is further validated by our DFT calculations. As expected, the investigated Ni_3_Co_1_-CPs features a lower energy barrier for the O_2_ release with the OPM pathway than the conventional adsorbate evolution mechanism (AEM).

Our study provides comprehensive mechanistic insights into the dynamic evolution of catalytically active species and sites during the OER, and highlights the potential of engineering CPs, whose well-defined coordination environments enable the accumulation of a high fraction of high-valent active species that are generally difficult to achieve in conventional oxide-related materials. These findings provide guidance for the rational design of future OER electrocatalysts by tailoring local coordination geometries to direct the formation of catalytically active sites.

## Supplementary Material



## Notes

Details on materials synthesis and characterizations, including PXRD, SEM, TEM, and XPS; additional electrochemical performance characterizations; *operando* XAS and Raman characterizations of Ni-CPs, NiCo-CPs, and NiCo-oxide; pH-dependent electrochemical analysis and DFT calculations (PDF)
