# Supplementary material for: Dynamics and Control of Dual Active Sites in Co-Substituted Ni Coordination Polymers for Enhanced Oxygen Evolution Catalysis
Source: J Am Chem Soc. 2026 Jan 20;148(4):3976–86. doi: 10.1021/jacs.5c12205 (PMC12879727; doi:10.1021/jacs.5c12205)
Supplement: Supplementary file 1 [file ja5c12205_si_001.pdf]

Supplementary Information

Dynamics and control of dual active sites in Co-substituted Ni coordination polymers for enhanced oxygen evolution catalysis

Yonggui Zhao,* Nanchen Dongfang, Rolf Erni, Marcella Iannuzzi, and Greta R. Patzke*

Dr. Y. Zhao, N. Dongfang, Prof. Dr. M. Iannuzzi, and Prof. Dr. G. R. Patzke
Department of Chemistry, University of Zurich, Winterthurerstrasse 190, CH-8057 Zurich, Switzerland

Prof. Dr. R. Erni
Electron Microscopy Center, Empa-Swiss Federal Laboratories for Materials Science and Technology, Überlandstrasse 129, CH-8600 Dübendorf, Switzerland
*Corresponding author
E-mail: yonggui.zhao@chem.uzh.ch/greta.patzke@chem.uzh.ch

Table of Contents

1. Experimental details and methods S3
2. Structural and morphological characterizations of the as-prepared catalysts S8
2. Electrocatalytic performance characterizations S18
3. Post-catalytic characterizations S23
3.1. Morphological characterizations of the as-prepared catalysts after OER stability measurements S23
3.2. XPS characterization of the as-investigated catalysts before and after OER stability measurements S24
3.3. XAS characterization of Ni-CPs after OER stability measurements S25
3.4. XAS characterization of Ni_3Co_1-CPs after OER stability measurements S26
3.3. XAS characterization of NiCo-CPs after OER stability measurements S27
3.3. XAS characterization of NiCo-oxide after OER stability measurements S28
4. *Operando* XAS characterizations of the as-investigated catalysts for the OER S29
4.1. *Operando* XAS characterizations of Ni-CPs for the OER S29
4.2. *Operando* quick-XAS characterizations of Ni_3Co_1-CPs for the OER S30
4.3. *Operando* XAS characterizations of NiCo-CPs for the OER S35
4.4. *Operando* XAS characterizations of NiCo-oxide for the OER S36
5. *Operando* Raman characterizations of the as-investigated catalysts for the OER S37
6. *Operando* EIS characterizations of the as-investigated catalysts for the OER S39
7. Pulse chronoamperometry measurements of the as-investigated catalysts S38
8. pH-dependent OER characterizations S41
9. Schematic illustration of the OER reaction pathway and free energy calculations S42
Table S1. At. % ratios of Ni/Co in the as-prepared catalysts based on ICP-MS and EDX analyses. S45
Table S2. Fitting parameters of the Ni *K*-edge EXAFS spectra for the as-prepared catalysts and references S45

Table S3. Fitting parameters of the Co *K*-edge EXAFS spectra for the as-prepared catalysts and references S46
Table S4. Comparison of OER performance of the as-prepared catalysts with recent representative studies S48
Table S5. ICP-MS results of freshly prepared electrolytes before and after the OER measurements...... S48
Table S6. Fitting parameters of *operando* Ni *K*-edge EXAFS spectra of Ni-CPs for the OER S49
Table S7. Fitting parameters of *operando* Ni *K*-edge EXAFS spectra of Ni_3Co_1-CPs for the OER S50
Table S8. Fitting parameters of *operando* Co *K*-edge EXAFS spectra of Ni_3Co_1-CPs for the OER S51
Table S9. Fitting parameters of *operando* Co *K*-edge EXAFS spectra of NiCo-CPs for the OER............. S39
Supplementary references S40

1. Experimental details and methods

Chemicals. Cobalt(II) chloride (≥98%), deuterium oxide (≥99.9%), ethylene glycol (EG) (≥99.8%), iron(III) nitrate nonahydrate (≥98%), nickel(II) chloride (≥98%), nickel(II) nitrate hexahydrate (≥98.5%), potassium tetracyanonickelate(II) hydrate (≥99%), potassium hydroxide (≥99.99%), potassium deuteroxide solution (40 wt.% in D_2O, 98 at.% D), polyvinylpyrrolidone (PVP) (M_w~10,000), tetramethylammonium hydroxide solution (TMAOH) (25 wt.% in H_2O), RuO_2 (≥99.9%), sodium citrate tribasic dihydrate (≥99%), urea (≥99.5%), and 5-(hydroxymethyl)furfural (HMF) (≥99%) were purchased from Sigma-Aldrich. All chemicals were used as received without any further purification.

Synthesis of nickel cyanide coordination polymers (Ni-CPs). To prepare Ni-CPs, a total of 259 mg of nickel(II) chloride, 0.8 g of PVP, and 441 mg of sodium citrate tribasic dihydrate were dissolved into 50 mL of deionized (DI) water to form solution A. At the same time, solution B was prepared by dissolution of 578 mg of potassium tetracyanonickelate(II) hydrate into 50 mL of DI water. Solution B was rapidly poured into solution A under magnetic stirring for 3 min. The mixture was then kept at room temperature for 24 h. The precipitate was collected by centrifugation, washed with ethanol/DI water at least three times, and dried at 70 ℃ overnight. Finally, the dried precipitate was annealed at 325 °C for 2 h with a heating rate of 2 °C/min under Ar atmosphere to obtain Ni-CPs. The preparation of Co-incorporated Ni-CPs was similar to the synthetic strategy for Ni-CPs except for partially replacing $NiCl_2$ with $CoCl_2$. The products obtained with different amounts of Co substitution were denoted as Ni_4Co_1-CPs, Ni_3Co_1-CPs, Ni_2Co_1-CPs, and NiCo-CPs. To explore the influence of PVP on the crystal structure and morphology of the as-prepared products, Ni_3Co_1-CPs were synthesized following the same protocol but without any PVP additive, referred to as Ni_3Co_1-CPs (No PVP). The corresponding sample obtained without subsequent annealing was denoted as Ni_3Co_1-CPs-RT (No PVP).

Synthesis of oxide references. Rock salt-type Ni_3Co_1-oxide and spinel-type NiCo-oxide were prepared by annealing the as-obtained Ni_3Co_1-CPs and NiCo-CPs in a muffle furnace at 350 °C in air for 2 h with a heating rate of 2 °C/min.

Synthesis of Ni_3Fe_1-layered double hydroxide (Ni_3Fe_1-LDH). In a typical synthetic route, a total of 0.3 mmol of nickel(II) nitrate hexahydrate, 0.1 mmol of iron(III) nitrate nonahydrate, and 0.2 mmol of urea were dissolved into 14 mL of DI water to obtain a homogeneous solution. The solution was then transferred into an 18 mL Teflon-lined stainless steel autoclave and kept

at 120 °C for 24 h. The precipitate was collected by centrifugation, washed with ethanol/DI water at least three times, and dried at 70 °C overnight.

Materials characterization. Powder X-ray diffraction (PXRD) patterns were recorded on a STOE STADI P diffractometer (transmission mode, Ge monochromator) with Cu K_α (λ = 1.54056 Å) radiation. Attenuated total reflectance Fourier-transform infrared (ATR-FTIR) spectra were recorded using a Bruker Vertex 70 spectrometer equipped with a Platinum ATR accessory containing a diamond crystal. Field-emission scanning electron microscopy (FESEM-Zeiss Supra 50 VP) equipped with energy-dispersive X-ray spectroscopy (EDX) and transmission electron microscopy (TEM-FEI Tecnai G2 Spirit) were used for morphology and elemental composition analyses. High-resolution TEM (HR-TEM), high angle annular dark field-scanning transmission electron microscopy (HAADF-STEM) and scanning transmission electron microscopy-energy dispersive X-ray spectroscopy (STEM-EDX) elemental mapping were performed on a FEI Titan Themis equipped with a hexapole-type aberration corrector for scanning transmission electron microscopy (CEOS DCOR) and a Super EDX system. X-ray photoelectron spectroscopy (XPS) studies were performed on a PerkinElmer PHI 1600 ESCA system with Mg K_α radiation (1253.6 eV). Inductively coupled plasma mass spectrometry (ICP-MS) was carried out on an Agilent 8800 Triple Quadrupole to determine the concentration of etched elements in the electrolyte, as well as the atomic ratios of Ni/Co in the as-prepared products.

Electrocatalytic oxygen evolution reaction (OER) characterization. Electrochemical OER measurements were performed at room temperature in 1 M Fe-free KOH with a standard three-electrode system (Metrohm Autolab PGSTAT302N potentiostat) using a reference Hg/HgO electrode (1 M KOH), a graphite rod as the counter electrode, and GC-RDE as the working electrode, respectively (**Note:** Fe-free KOH electrolyte was prepared through a high-purity nickel hydroxide adsorption strategy, as reported in our previous study [1]).

The working electrode was prepared through a standard drop-coating protocol.[2] Typically, a total of 3.0 mg of catalysts, 1 mg of carbon black, and 30 μL of 5 wt% Nafion solution were homogeneously dispersed in 600 μL of ethanol to obtain the catalyst inks by an ultrasonication-assisted approach. Then, 10 μL of inks were loaded on a glassy carbon rotating disk electrode (GC-RDE, diameter of 5 mm) to reach a loading mass of ca. 0.25 $mg/cm_{geo.}^2$ [**Note:** For the *operando* XAS and Raman studies, the catalyst inks were prepared without any carbon black additive.].

Prior to the electrochemical OER measurements, the working electrode was polarized with 50 scans of cyclic voltammetry (CV) at 50 mV/s to reach a steady state. Then, a low scan rate of 5 mV/s with a rotation of 1600 rpm was applied to record the OER CV curve. All potentials were converted to the reversible hydrogen electrode (RHE) scale using the equation E(RHE)=E+E(ref.) + 0.059 × pH. A 90 % iR-compensation was then applied to the recorded OER CV curves, in which the uncompensated ohmic contact resistance (R) was identified by the electrochemical impedance spectroscopy (EIS) tests at open-circuit potential (10 mHz to 100 kHz and 5 mV amplitude). Electrochemically active surface area (ECSA) was identified based on a CV method with different scan rates within a specific potential region. Then, the ECSA was calculated using the equation: ECSA=geometric surface area × C_{dl}/C_0 (C_0 = 0.04 mF/cm^2). The OER Faradaic efficiency was quantified based on the rotating ring-disk electrode (RRDE) technique as follows: Faradaic efficiency = $i_{ring}/(i_{disk} \times N)$, where i_{ring} and i_{disk} represent the ring and disk currents, respectively, and N is a constant value of 0.2. Long-term durability tests were performed with carbon paper as the working electrode at a constant current density of 20 mA/cm^2.

***Operando* Raman characterizations**. Raman spectra were recorded on a Renishaw Raman scope or InVia Qontor (Ar+ laser, 532). An in-house designed electrochemical cell equipped with a screen-printed electrode (SPCE, Dropsens, DRP-110) was employed for the *operando* Raman investigations. To perform *operando* electrochemical experiments, the working area of the SPEC was covered by a thin layer of the electrolyte. Then, chronoamperometry method with a stepwise increase of the applied potential was implemented to record the operando Raman signals.

***Ex situ* and *operando* X-ray absorption spectroscopy (XAS) characterizations.** *Ex situ* and *operando* XAS experiments were performed at the SuperXAS-X10DA beamline at the Swiss Light Source (PSI, Switzerland) (*operando* experiments of Co_3Ni_1-CPs) and at the KMC-2 beamline at the Helmholtz Zentrum Berlin (HZB) BESSY II (Berlin, Germany) (*operando* experiments of Ni-CPs, NiCo-CPs, and NiCo-oxide). All the XAS data were recorded via fluorescence modes with a Si(111) double crystal monochromator (DCM) cooled with liquid nitrogen, respectively. The experiments at the SuperXAS-X10DA beamline were conducted in the quick-scanning extended X-ray absorption fine-structure (QEXAFS) mode with the monochromator oscillation frequency of 60 Hz (120 spectra per minute). Subsequently, several QEXAFS spectra collected under the same conditions were averaged together to achieve a reasonable signal-to-noise ratio. The experiments at the KMC-2 beamline were conducted in a conventional stepwise scan mode (ca. 15 min per spectrum).

For the *ex situ* characterizations, solid powder samples were diluted in cellulose and pressed into a pellet to achieve an absorption step of ca. 1. Co (7709 eV) and Ni foils (8333 eV) were used as standard reference samples for Co and Ni *K*-edge energy calibration, respectively. An in-house developed electrochemical cell equipped with a standard three-electrode system was used to perform the *operando* monitoring experiments.[3] During the experiments, a chronoamperometry method with a stepwise increase of the applied potentials was implemented to collect the *operando* XAS spectra. Note that the collected raw XAS data from the SuperXAS-X10DA beamline were averaged and normalized using the ProQEXAFS software package, and the XAS data collected from the KMC-2 beamline were directly analyzed through the ATHENA software package. The k^3-weighted EXAFS data were analyzed in the k-range from 0 to 12 $Å^{-1}$. The Fourier transform (FT) spectra were analyzed within the R-range from 0 to 6 Å. Fitting of EXAFS spectra was conducted within the R-range from 1 to 3 Å. Note that phase corrections were not considered in the EXAFS spectra.

Density function theory (DFT) calculations.

DFT calculations were performed using the CP2K package.[4] Interactions within the atomic cores were described using Goedecker–Teter–Hutter (GTH) pseudopotentials, and Gaussian-type orbitals were used to expand the molecular orbitals of the valence.[5] The plane-wave cut-off energy was set to 400 Ry, and DZVP-MOLOPT-GTH basis sets were applied to all elements. The exchange-correlation functional was Perdew-Burke-Ernzerhof (PBE) generalized gradient approximation (GGA), and D3 correction was used to account for long-range dispersion interactions.[6,7] The NiO_2 (012)-terminated surface, obtained by removal of all H atoms from γ-NiOOH, was chosen as a computational model. Co atoms were introduced by replacing partial Ni atoms. A 3×2×1 supercell structure containing a total of 252 atoms was used for all simulations. Broyden-Fletcher-Goldfarb-Shanno (BFGS) scheme with a force threshold of 1×10^{-3} Hartree/Bohr was applied to optimize all proposed models.[8] A 20 Å thickness of vacuum layer was added to the built supercell (both above and below) to eliminate surface interaction effects.

For the OER under alkaline conditions, the following equations (eqs. S1-S4) are employed for describing the adsorbate evolution mechanism (AEM):

$$*+OH^- \rightarrow *OH+e^- \quad \text{(eq. S1)}$$

$$*OH+OH^- \rightarrow *O+H_2O+e^- \quad \text{(eq. S2)}$$

$$*O+OH^- \rightarrow *OOH+e^- \quad \text{(eq. S3)}$$

$$*OOH+OH^- \rightarrow O_2+H_2O+e^- \quad \text{(eq. S4)}$$

Where * indicates a catalytically active adsorption site, and *OH, *O, and *OOH represent the adsorption of key OER intermediates on the active site.

Gibbs free energy changes (ΔG) of the individual elementary steps in the AEM are calculated as follows:

$$\Delta G_1=E_{*OH}-E_{*}-E_{H2O}+1/2E_{H2}+(\Delta ZPE-T\Delta S)_1-eU \quad \text{(eq. S5)}$$

$$\Delta G_2= E_{*O}-E_{*OH} +1/2E_{H2}+(\Delta ZPE-T\Delta S)_2-eU \quad \text{(eq. S6)}$$

$$\Delta G_3= E_{OOH*}-E_{O*}-E_{H2O}+1/2E_{H2}+(\Delta ZPE-T\Delta S)_3-eU \quad \text{(eq. S7)}$$

$$\Delta G_4=4.92\ eV-E_{*}+E_{OOH*}-E_{O2}-1/2E_{H2}-(\Delta ZPE-T\Delta S)_4-eU \quad \text{(eq. S8)}$$

The theoretical overpotential η_{theory} is calculated based on the equation:

$$\eta_{theory}=\max\{\Delta G_1, \Delta G_2, \Delta G_3, \Delta G_4\}/e-1.23V \quad \text{(eq. S9)}$$

In eqs.S5-S8, E_{M*} represents the binding energy associated with the OER intermediate adsorption. ΔZPE and ΔS are the changes in zero-point energy and entropy corrections, respectively. An additional bias (U) is included to calculate the reaction-free energy for each elementary step. All the reactions in this study occur at room temperature; hence, temperature-dependent effects were not taken into account.

In comparison, the OPM can be described by the following equations:

$$*+*+OH^- \rightarrow *+*OH+e^- \quad \text{(eq. S10)}$$

$$*+*OH+OH^- \rightarrow *OH+*OH+e^- \quad \text{(eq. S11)}$$

$$*OH+*OH+OH^- \rightarrow *OH+*O+H_2O+e^- \quad \text{(eq. S12)}$$

$$*OH+*O+OH^- \rightarrow *O\text{-}O*+H_2O+e^- \quad \text{(eq. S13)}$$

$$*O\text{-}O* \rightarrow *+*+O_2 \quad \text{(eq. S14)}$$

Gibbs free energy changes (ΔG) of the individual elementary steps in the OPM are calculated as follows:

$$\Delta G_1=E_{*+*OH}-E_{*+*}-E_{H2O}+1/2E_{H2}+(\Delta ZPE-T\Delta S)_1-eU \quad \text{(eq. S15)}$$

$$\Delta G_2= E_{*OH+*OH} -E_{*+*OH}-E_{H2O}+1/2E_{H2}+(\Delta ZPE-T\Delta S)_2-eU \quad \text{(eq. S16)}$$

$$\Delta G_3= E_{*OH+*O}-E_{*OH+*OH}+1/2E_{H2}+(\Delta ZPE-T\Delta S)_3-eU \quad \text{(eq. S17)}$$

$$\Delta G_4= E_{*O\text{-}O*}-E_{*OH+*O} +1/2E_{H2}+(\Delta ZPE-T\Delta S)_4-eU \quad \text{(eq. S18)}$$

$$\Delta G_5= E_{*+*}-E_{*O\text{-}O*} +E_{O2}+(\Delta ZPE-T\Delta S)_5-eU \quad \text{(eq. S19)}$$

2. Structural and morphological characterizations of the as-prepared catalysts

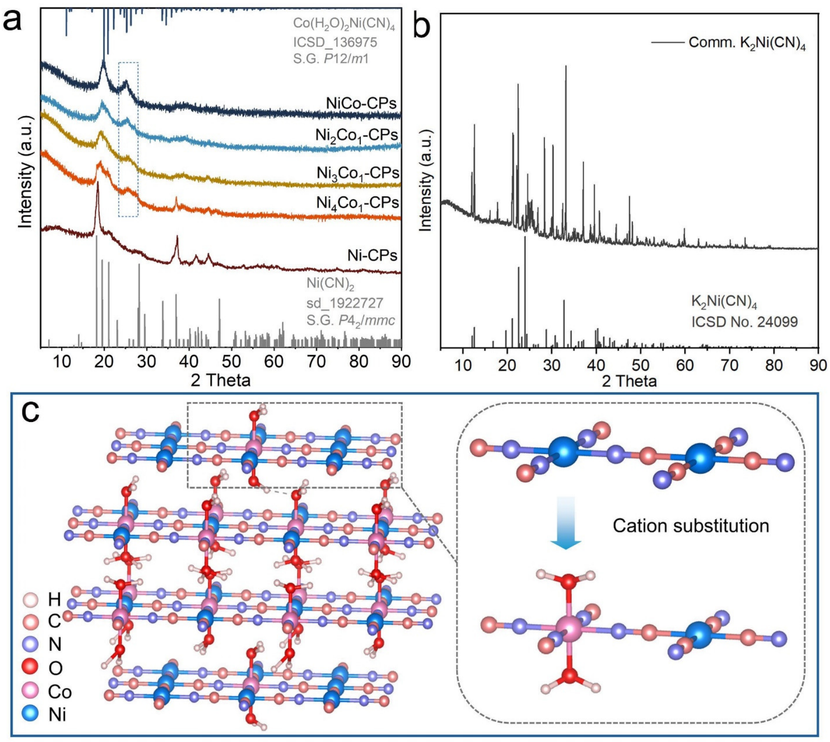

Figure S1. (a) PXRD patterns of as-prepared Ni-CPs and Co-substituted products. (b) PXRD pattern of commercial $K_2Ni(CN)_4$. (c) Structural model of Co-substituted Ni-CPs.

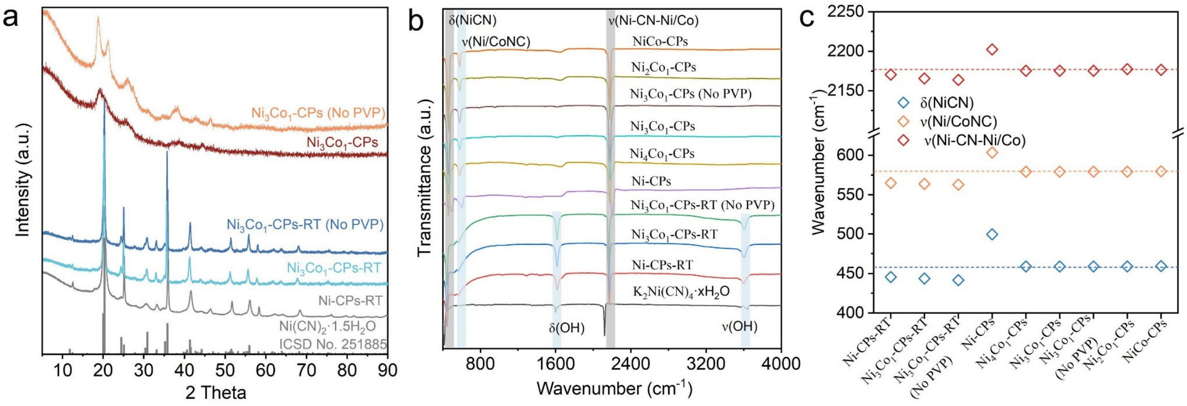

Figure S2. PXRD patterns (a) and FTIR spectra (b) of as-prepared Ni-CPs and Co-substituted products. (c) Comparison of the wavenumbers of three vibration modes.

As described in the "Experimental details and methods" section, Ni-CPs were prepared via a room-temperature coprecipitation approach, followed by annealing under Ar atmosphere. The PXRD pattern (**Figure S1a**) of the as-prepared Ni-CPs matches well with the simulated pattern of the cyanide-bridged coordination polymer of $Ni(CN)_2$ (Springer Materials: sd_1922727, S.G. *P*4₂/*mmc*), exhibiting a highly disordered crystal structure.[9] In the lattice of Ni-CPs, Ni(II) centers are coordinated by four cyanide groups to form extended NC-Ni-CN-Ni-NC motifs (**Figure S1c**). To obtain detailed insights into the structural evolution, we also examined the PXRD pattern of the precursor prior to annealing, referred to as Ni-CPs-RT. Our results (**Figure S2a**) show that Ni-CPs-RT exhibit a diffraction pattern consistent with that simulated for $Ni(CN)_2\cdot1.5H_2O$ (ICSD: No_251885). Unlike Ni-CPs, which contain only square-planar $\{Ni(CN/NC)_4\}$ coordination geometries, two different Ni sites are present in Ni-CPs-RT: an

octahedral site coordinated by four N and two O atoms in a $\{Ni(NC)_4(OH_2)_2\}$ coordination geometry, and a square-planar site with $\{Ni(CN)_4\}$ coordination geometry. Notably, the Ni-N bond length (ca. 2.05 Å) in Ni-CPs-RT is significantly longer than that in Ni-CPs (ca. 1.87 Å), which alters the intrinsic vibrational characteristics of the NC-Ni-CN-Ni-NC motifs, as revealed by FTIR spectroscopy. As shown in **Figure S2b,c**, Ni-CPs-RT exhibit three characteristic FTIR bands at ca. 446 (sharp), 565 (weak), and 2171 (sharp) cm^{-1}, associated with δ(NiCN), and ν(NiNC), and ν(Ni-CN-Ni) vibrations, respectively. After dehydration of Ni-CPs-RT into Ni-CPs, these bands shift towards higher wavenumber, appearing at ca. 500 (sharp), 604 (sharp), 2202 (sharp) cm^{-1}. This blueshift indicates a decrease in the Ni-N bond length within the Ni-CN-Ni moiety of Ni-CPs compared to that of Ni-CPs-RT, consistent with our above PXRD analysis.

The PXRD patterns of Co-substituted Ni-CPs display broader diffraction features compared to pristine Ni-CPs (**Figure S1**), and the observed additional peaks at ca. 25° can be assigned to $Co(H_2O)_2Ni(CN)_4$ (ICSD_136975, $Fe(H_2O)_2Ni(CN)_4(C_4H_8O_2)_2$, S.G. *P*12/*m*1). Note that the simulated PXRD pattern of $Co(H_2O)_2Ni(CN)_4$ was obtained from the standard $Fe(H_2O)_2Ni(CN)_4(C_4H_8O_2)_2$ phase by replacing the Fe with Co and removing the coordinated $C_4H_8O_2$ molecules via the Materials Studio software package. This difference can be attributed to the preference of Co(II) centers for an octahedral coordination environment, adopting a $\{Co(NC)_4(OH_2)_2\}$ motif in $Ni_{2-x}Co_x$-CPs ($0 < x \leq 1$), which can induce substantial structural disorder. To shed light on the role of Co substitution in the structural evolution of Ni-CPs-RT after dehydration, Ni_3Co_1-CPs-RT was newly prepared and selected as a representative material. As shown in **Figure S2a**, its PXRD pattern closely resembles that of Ni-CPs-RT, suggesting the successful substitution of Co into the Ni-CPs-RT lattice. Compared to Ni-CPs-RT, the FT-IR spectrum of Ni_3Co_1-CPs-RT (**Figure S2b**) shows slight redshifts of three characteristic vibrations, of which appear at 444 (sharp), 564 (weak), and 2166 (sharp) cm^{-1}. Dehydration of Ni_3Co_1-CPs-RT into Ni_3Co_1-CPs results in band shifts towards higher wavenumbers (sharp-459, sharp-580, and sharp-2175 cm^{-1}), yet remain significantly lower wavenumbers than those of Ni-CPs. Similar vibrational signatures were also observed for other Co-substituted Ni-CPs, indicating that dehydration predominantly occurs at Ni sites. This leads to a heterogeneous framework comprising $\{Co(NC)_4(OH_2)_2\}$ and $\{Ni(CN/NC)_4\}$ motifs with abundant structural disorder in the Co-substituted Ni-CPs. On this basis, Co-substituted Ni-CPs can be determined as $Ni_{1-x}Co_x(H_2O)_{2x}Ni(CN)_4$. To precisely identify the local coordination environments of Ni and Co centers of the prepared CPs, advanced spectroscopic characterizations such as XAS are essential (cf. detailed discussion in the following sections).

To further investigate the role of PVP as a synthesis additive in regulating the crystal structure and morphology of CP materials, Ni_3Co_1-CPs-RT (No PVP) and Ni_3Co_1-CPs (No PVP) were prepared and characterized through PXRD and FTIR. The obtained results (**Figure S2**) demonstrate that the absence of PVP does not affect the crystal structure or vibrational signatures of Ni_3Co_1-CPs. In contrast, microscopic investigations (**Figures S4, S10, and S11**) reveal that these two newly prepared samples exhibit random nanosheet morphologies, with no evidence for nanocluster formation. From the FESEM-EDX results (**Figures S12 and S13**), the atomic ratios of Ni/Co in Ni_3Co_1-CPs-RT (No PVP) and Ni_3Co_1-CPs (No PVP) were determined as 2.50:1 and 2.48:1, respectively, albeit not identical with the target composition of Ni_3Co_1-CPs (3.05 from FESEM-EDX and 3.07 from ICP-MS, **Table S1**). These discrepancies mainly result from the fact that Co^{2+} more readily coordinates with $[Ni(CN)_4]^{2-}$ due to its higher Lewis acidity and stronger affinity for the nitrogen site compared to Ni^{2+}. In

the absence of PVP, a fraction of the Ni ions was not involved in the nucleation process but remained in the solution, resulting in a slightly lower Ni/Co atomic ratio. The influence of PVP additive on the OER kinetics of Ni_3Co_1-CPs was further explored through electrochemical OER measurements and pulse chronoamperometry characterizations, as detailed in **Figures S25 and S52**.

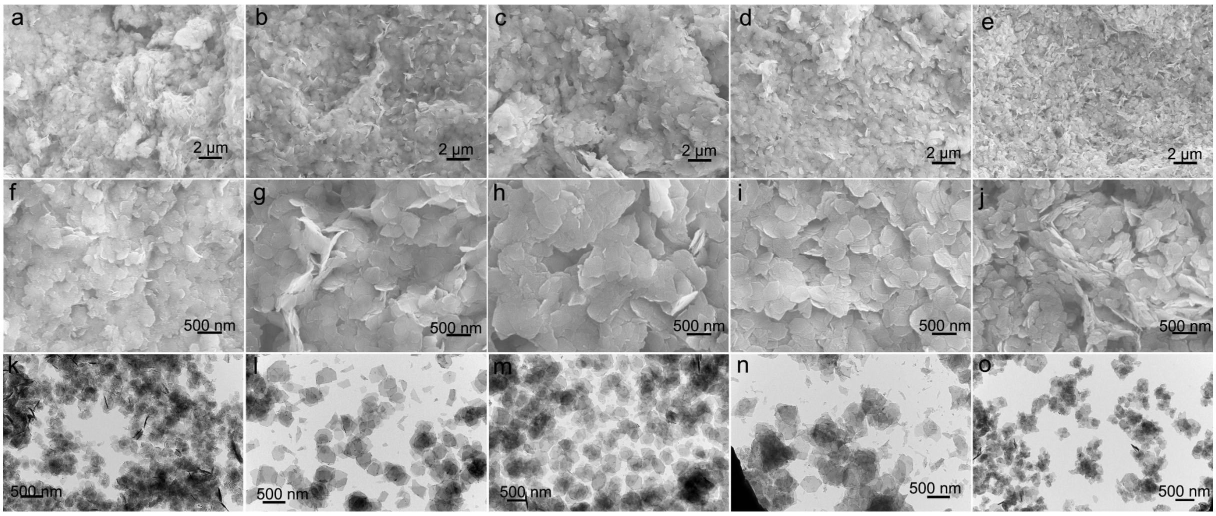

Figure S3. FESEM (a-j) and TEM (k-o) images of as-prepared Ni-CPs and Co-substituted products: (a, f, k) Ni-CPs; (b, g, l) Ni_4Co_1-CPs; (c, h, m) Ni_3Co_1-CPs; (d, i, n) Ni_2Co_1-CPs; (e, j, o) NiCo-CPs.

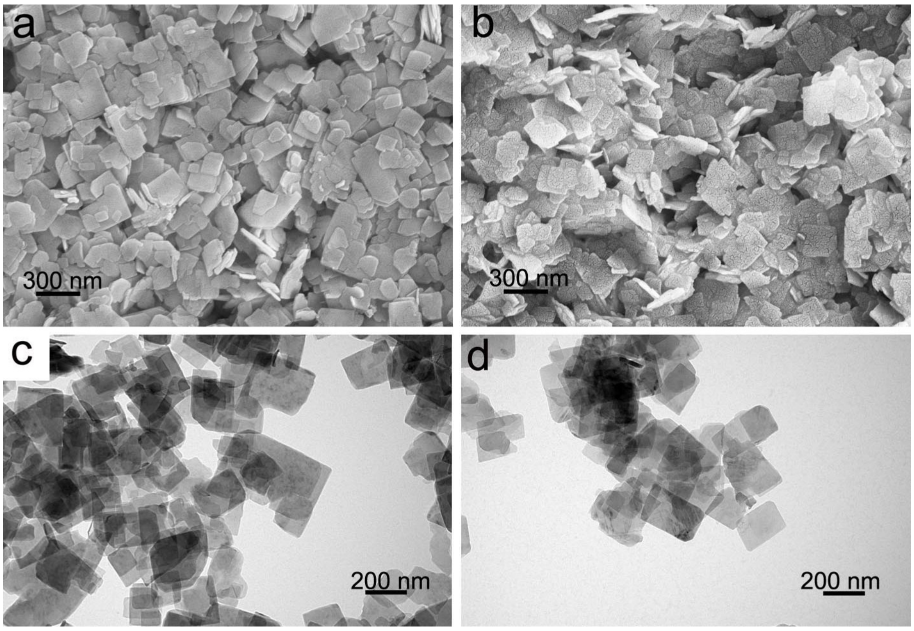

Figure S4. (a, c) FESEM and TEM images of as-prepared Ni_3Co_1-CPs-RT (No PVP). (b, d) FESEM and TEM images of as-prepared Ni_3Co_1-CPs (No PVP).

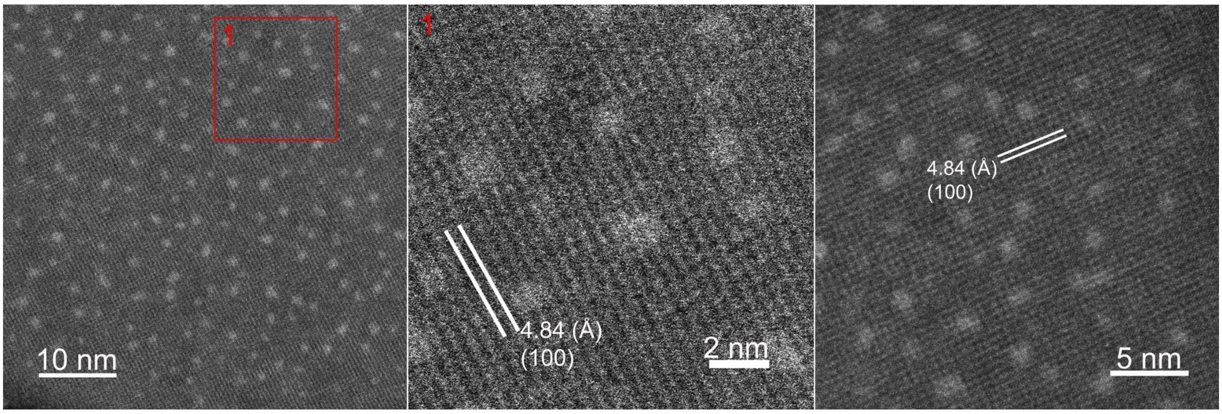

Figure S5. HR-TEM images of as-prepared Ni-CPs.

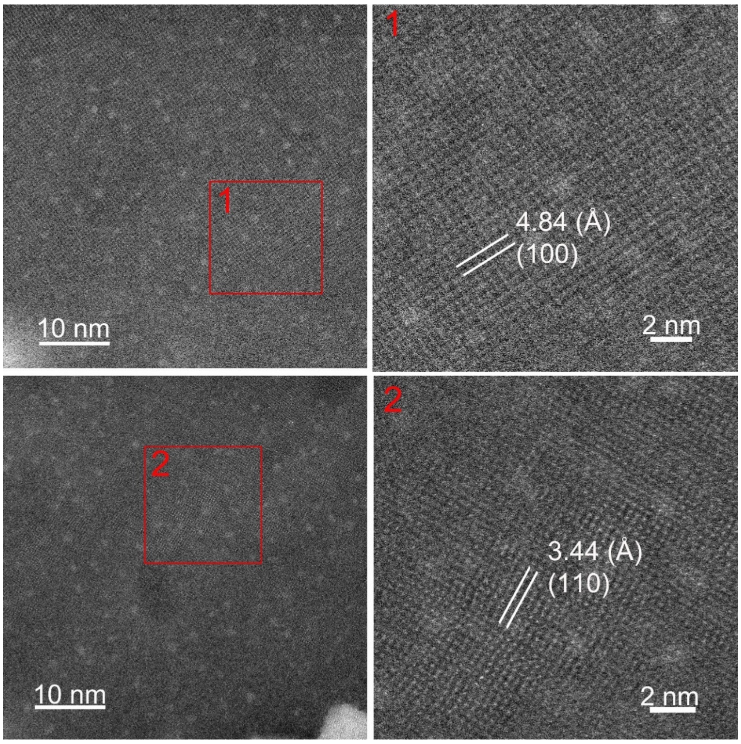

Figure S6. HR-TEM images of as-prepared Ni_3Co_1-CPs.

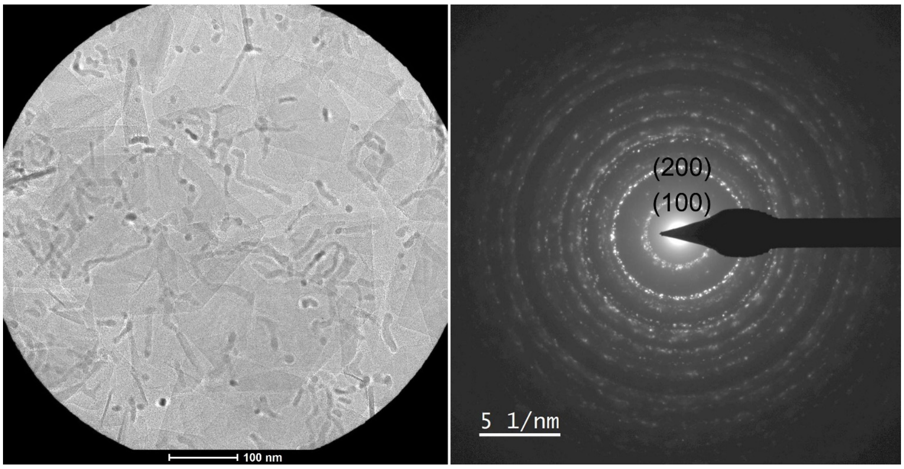

Figure S7. TEM image and the corresponding SAED pattern of as-prepared Ni-CPs.

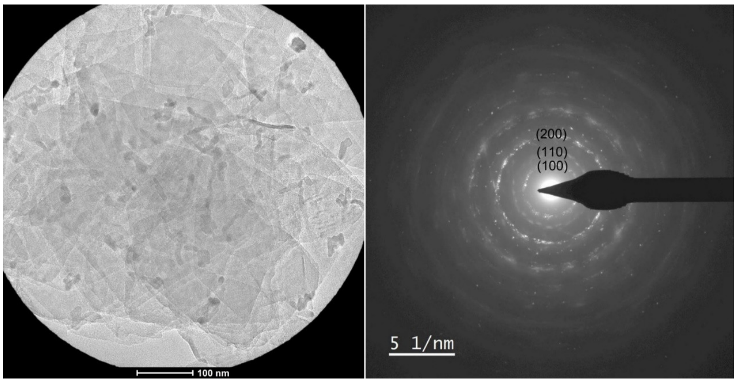

Figure S8. TEM image and the corresponding SAED pattern of as-prepared Ni_3Co_1-CPs.

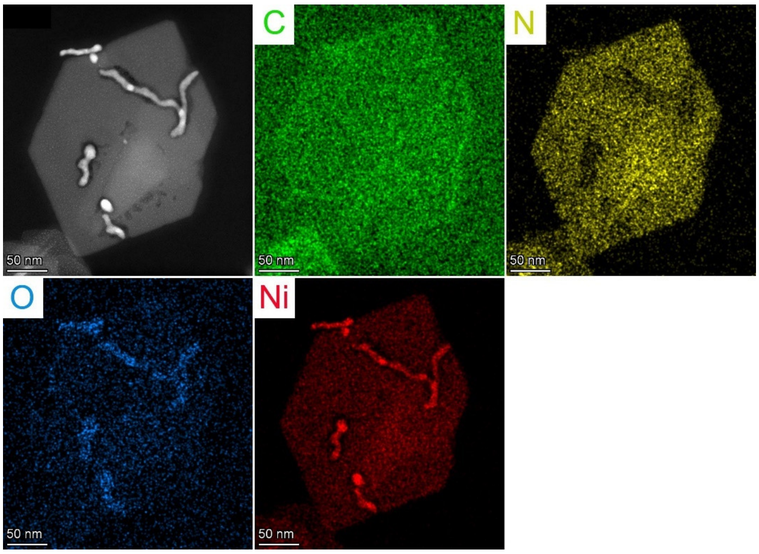

Figure S9. STEM-EDX element mappings of as-prepared Ni-CPs.

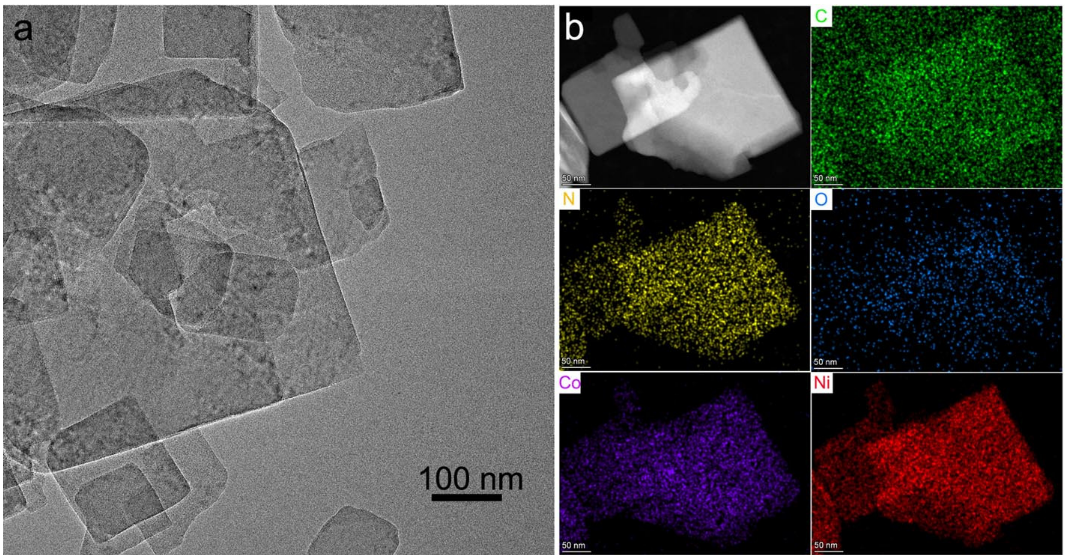

Figure S10. TEM image (a) and STEM-EDX element mappings (b) of as-prepared Ni_3Co_1-CPs-RT (No PVP).

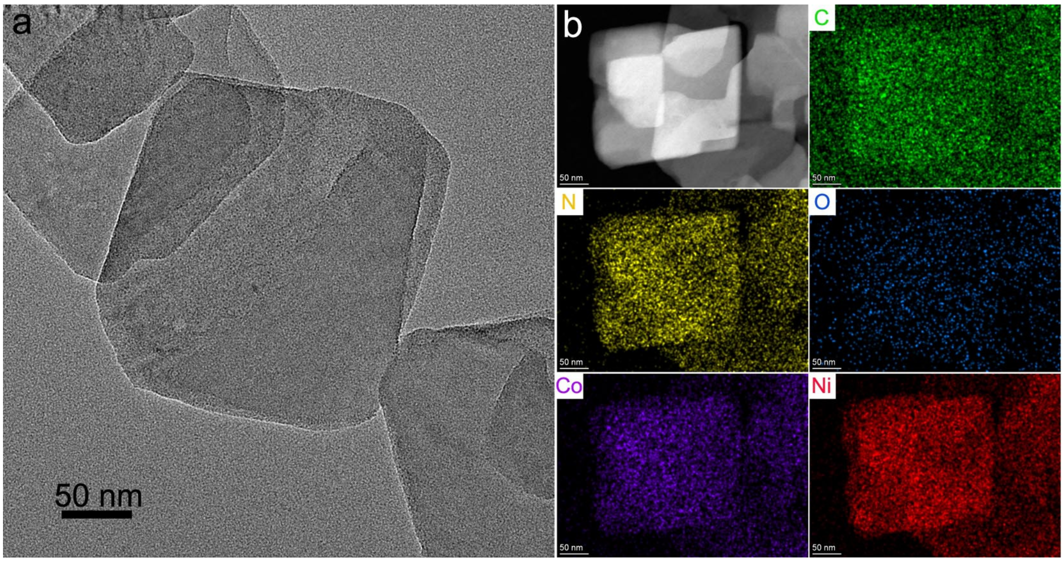

Figure S11. TEM image (a) and STEM-EDX element mappings (b) of as-prepared Ni_3Co_1-CPs (No PVP).

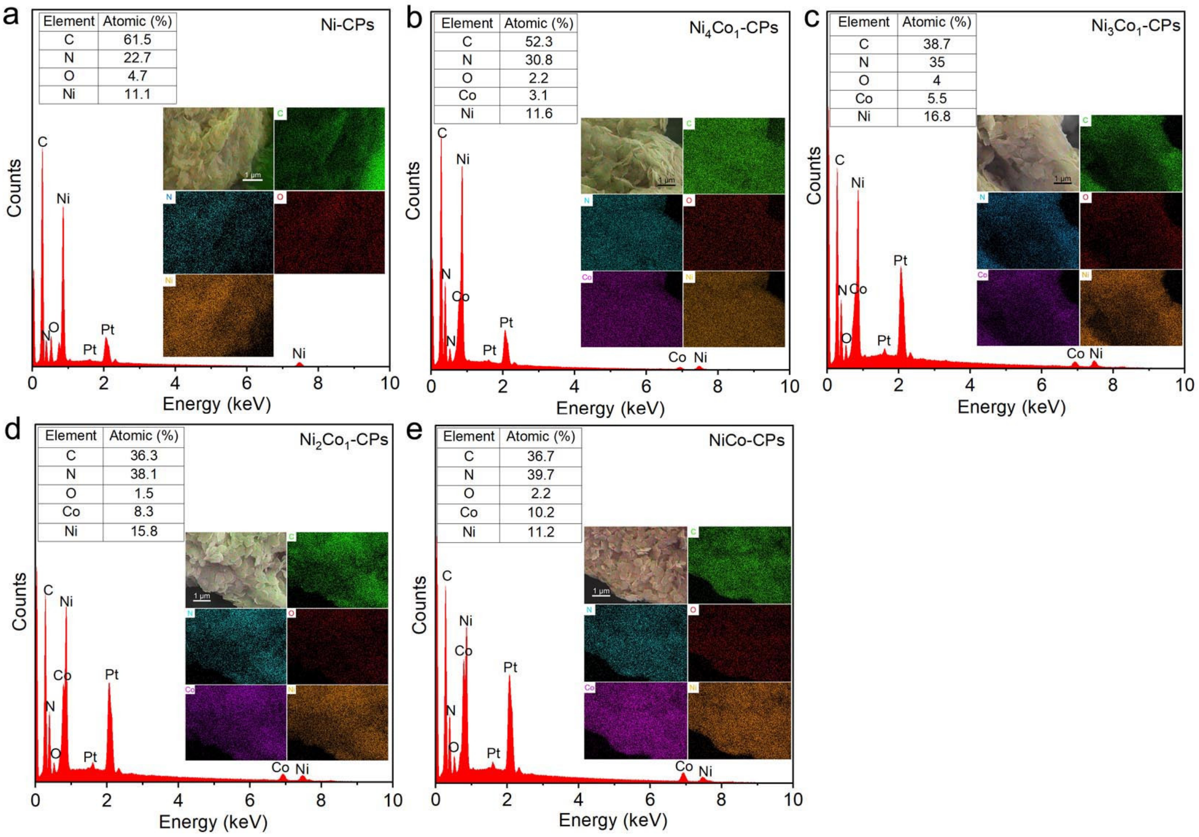

Figure S12. FESEM-EDX spectra and element mapping images of as-prepared Ni-CPs and Co-substituted products: (a) Ni-CPs; (b) Ni_4Co_1-CPs; (c) Ni_3Co_1-CPs; (d) Ni_2Co_1-CPs; (e) NiCo-CPs.

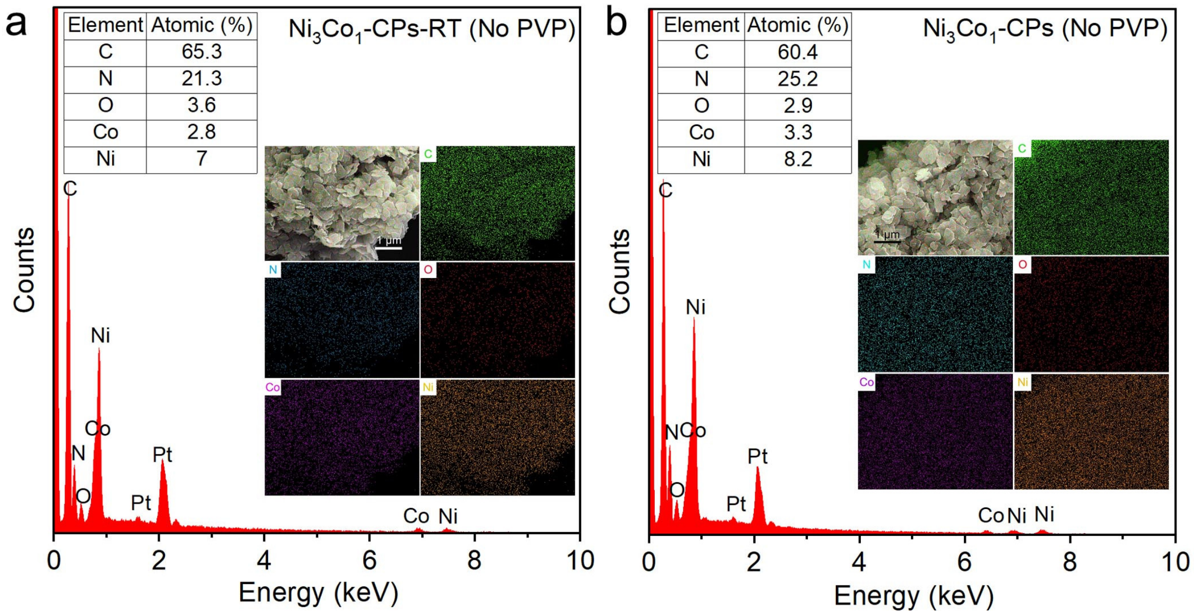

Figure S13. (a, b) FESEM-EDX spectra and element mapping images of as-prepared Ni_3Co_1-CPs-RT (No PVP) and Ni_3Co_1-CPs (No PVP).

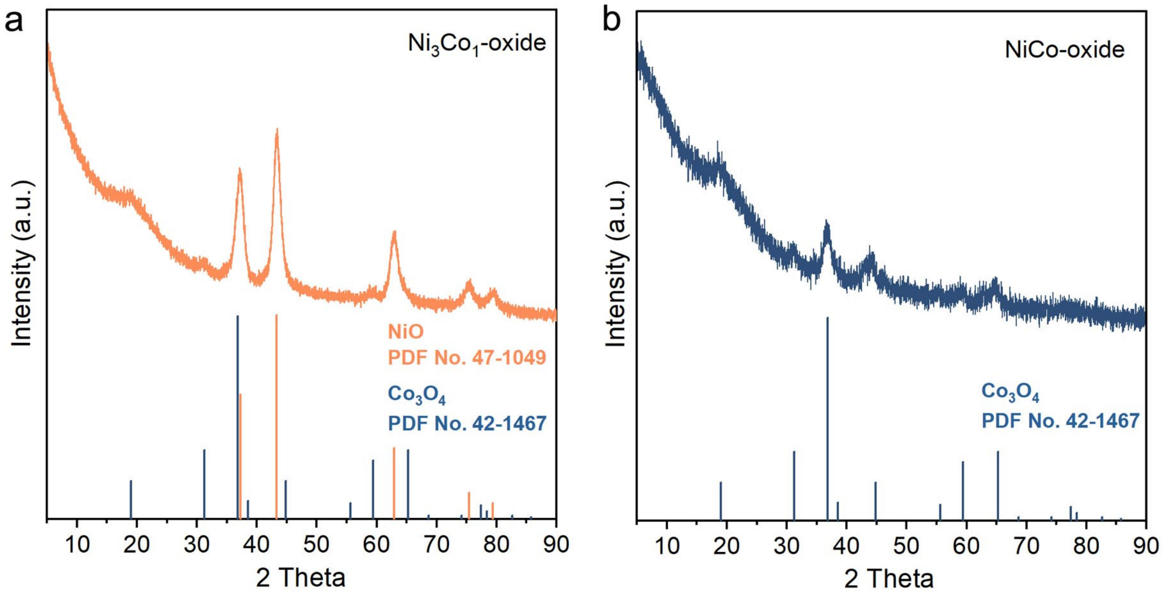

Figure S14. PXRD patterns: (a) rock salt-type Ni_3Co_1-oxide; (b) spinel-type NiCo-oxide.

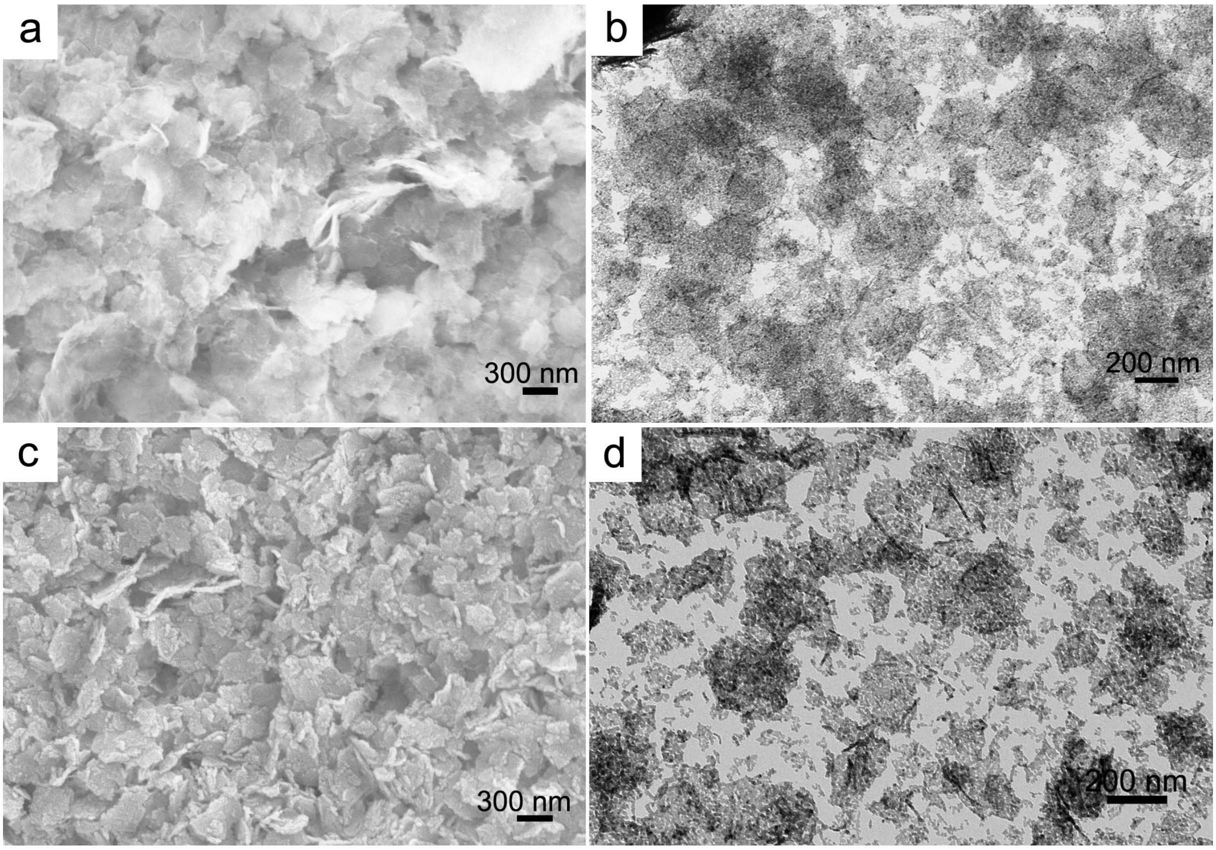

Figure S15. FESEM and TEM images: (a, b) rock salt-type Ni_3Co_1-oxide; (c, d) spinel-type NiCo-oxide.

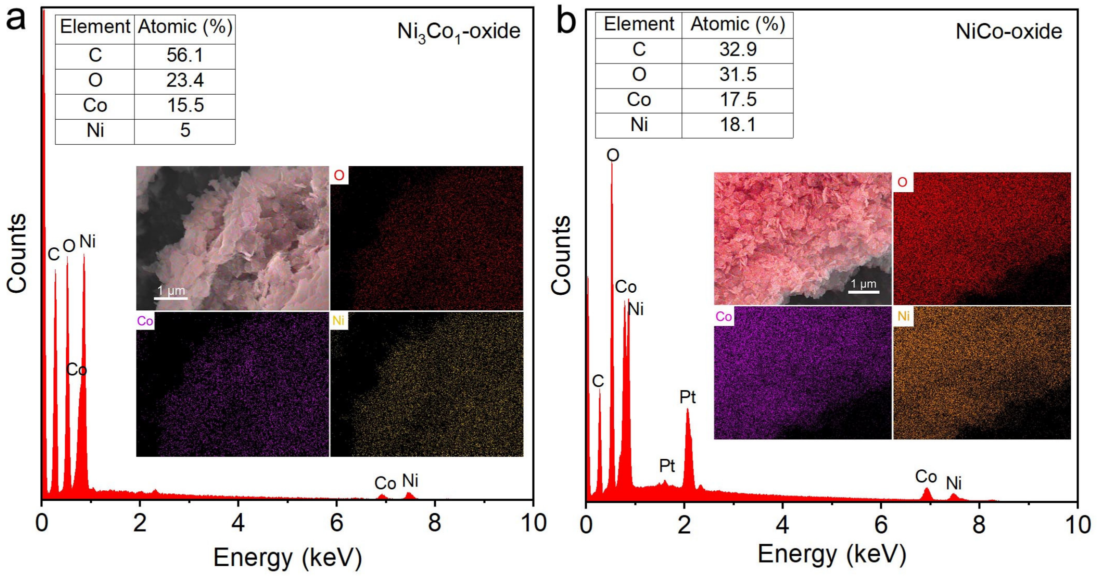

Figure S16. FESEM-EDX spectrum and element mappings: (a) rock salt-type Ni_3Co_1-oxide; (b) spinel-type NiCo-oxide.

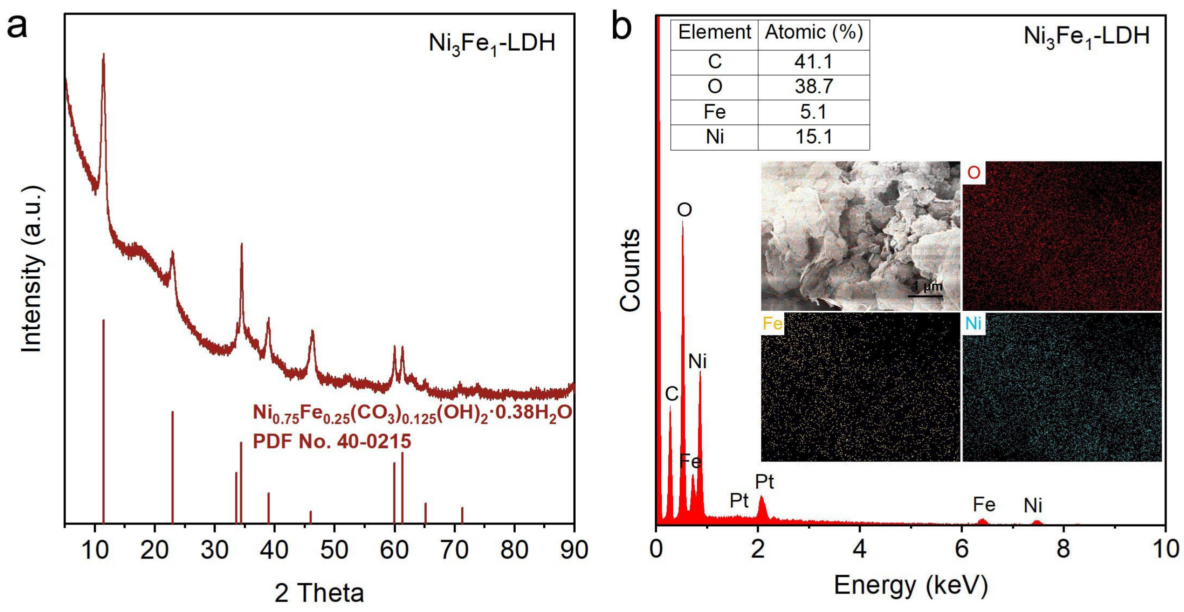

Figure S17. (a) PXRD pattern of Ni_3Fe_1-LDH. (b) FESEM-EDX spectrum and element mappings of Ni_3Fe_1-LDH.

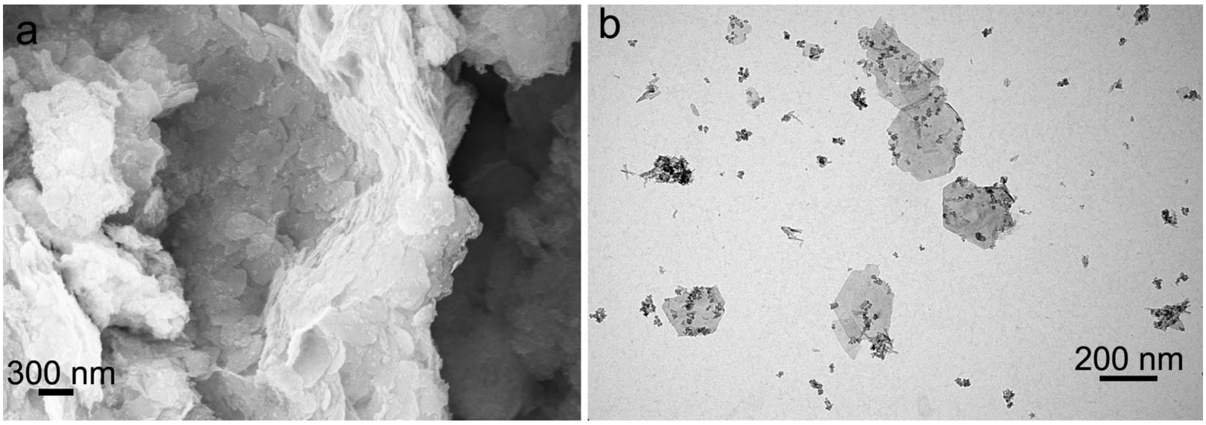

Figure S18. (a, b) FESEM and TEM images of Ni_3Fe_1-LDH.

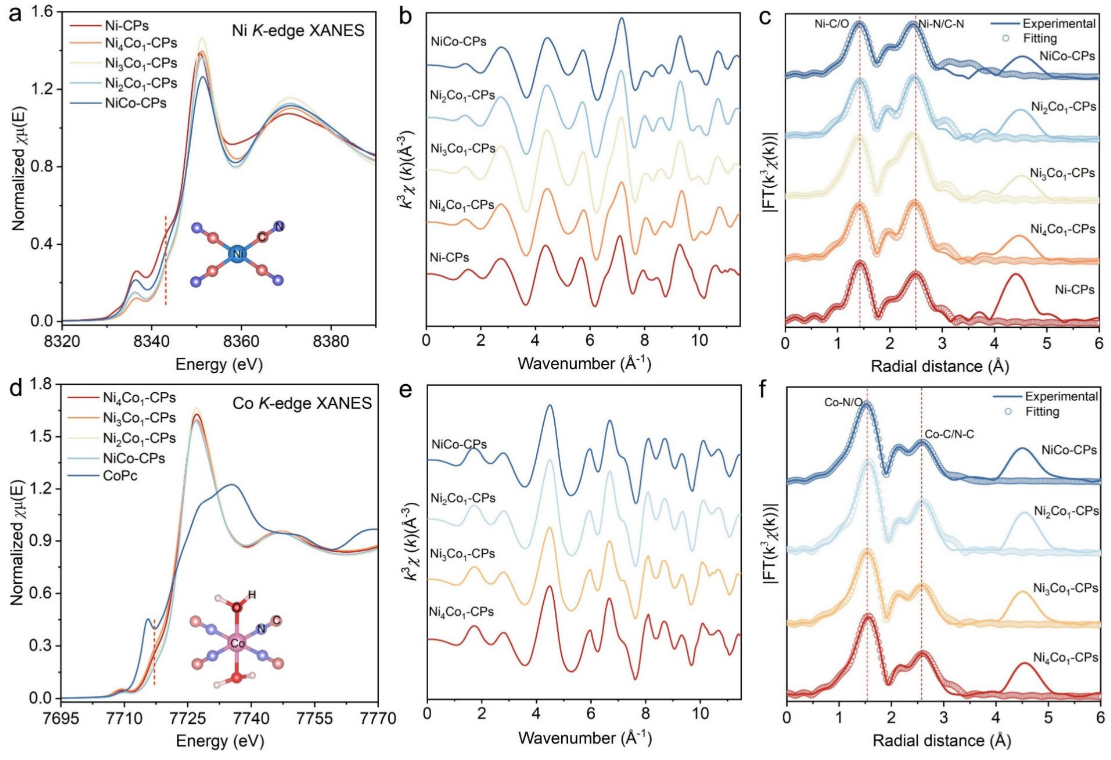

Figure S19. Ni (a-c) and Co (d-f) *K*-edge XAS characterizations of as-prepared Co-substituted Ni-CPs samples: (a, d) XANES spectra; (b, e) EXAFS spectra; (c, f) Fitting of FT-EXAFS spectra.

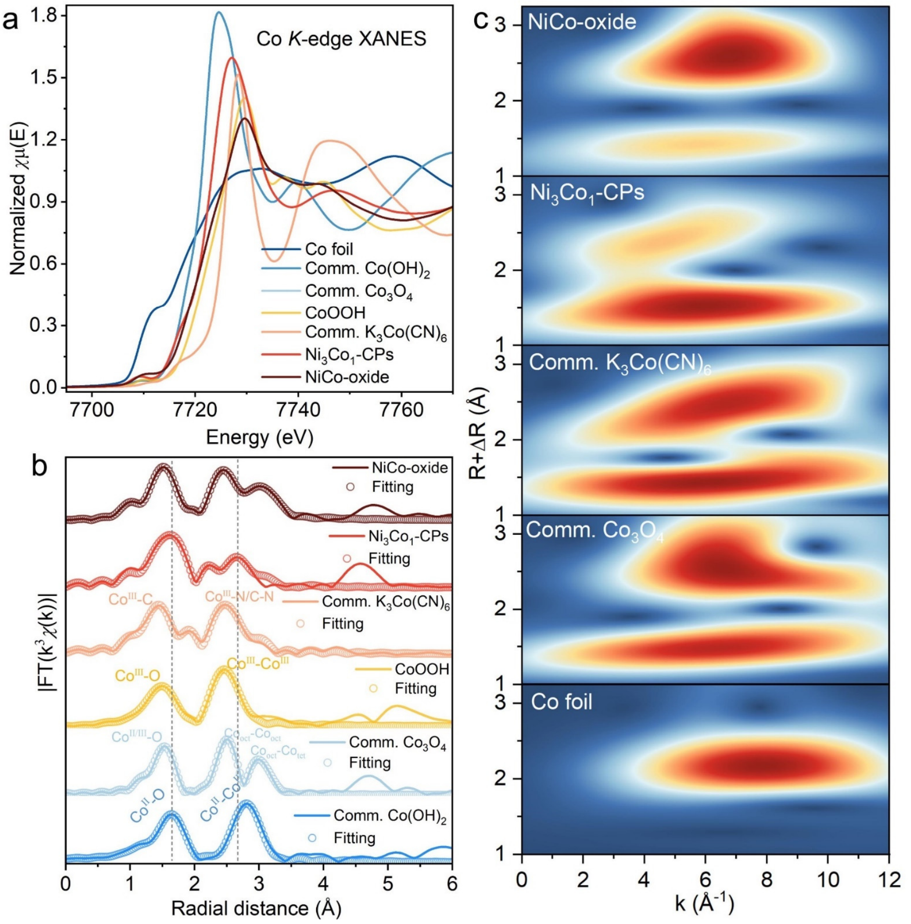

Figure S20. Co *K*-edge XAS characterizations of as-investigated samples and references: (a) XANES spectra; (b) Fitting of FT-EXAFS spectra; (c) WT contour profiles.

2. Electrocatalytic performance characterizations

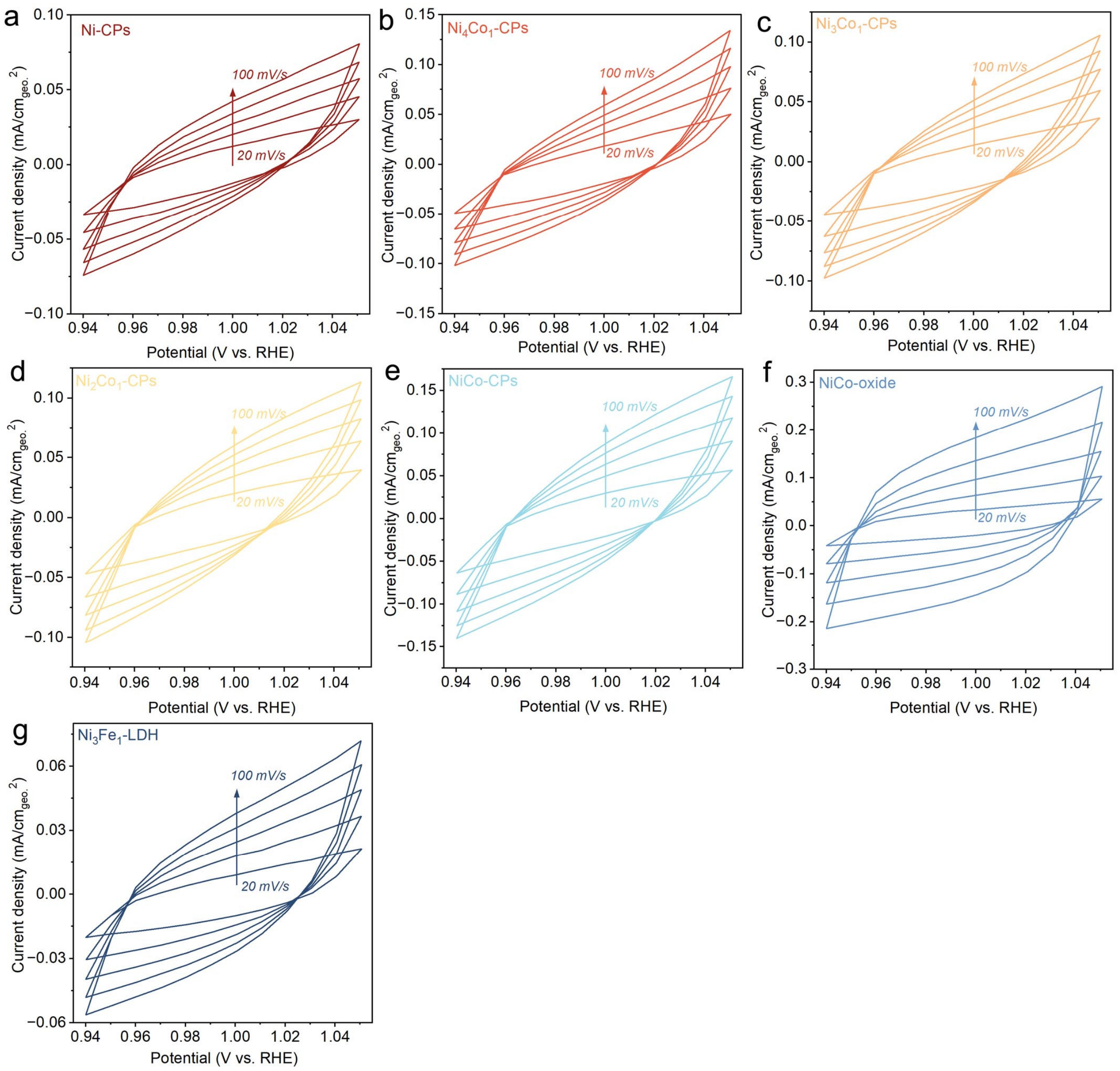

Figure S21. CV curves for as-investigated catalysts: (a) Ni-CPs; (b) Ni_4Co_1-CPs; (c) Ni_3Co_1-CPs; (d) Ni_2Co_1-CPs; (e) NiCo-CPs; (f) NiCo-oxide; (g) Ni_3Fe_1-LDH.

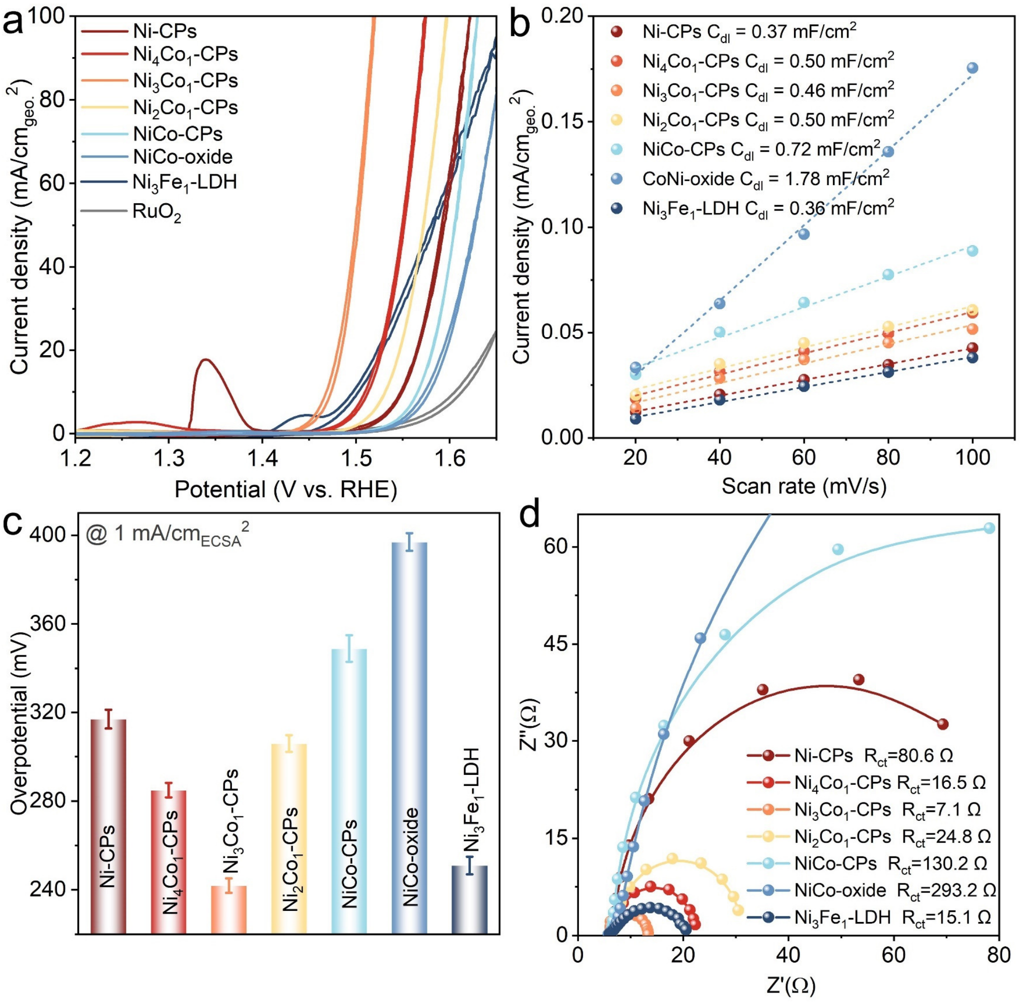

Figure S22. Electrocatalytic OER performance of Co-substituted Ni-CPs in 1 M KOH (pH ~ 13.7, scan rate = 5 mV/s, mass loading = 0.25 mg/cm^2, and rotation speed = 1600 rpm): (a) 90% iR-corrected CV curves; (b) Calculated C_{dl} values; (c) Comparison of overpotentials at 1 mA/cm$_{ECSA}^2$; (d) Nyquist plots (at 1.5 V vs. RHE).

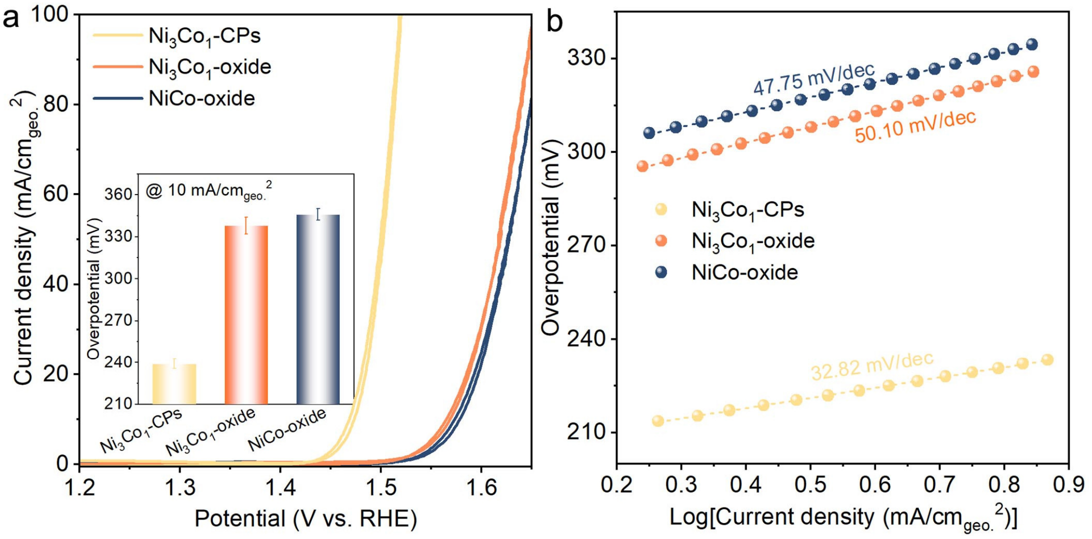

Figure S23. (a) CV curves of Ni_3Co_1-CPs and oxide reference. (b) Tafel plots. (**Note:** post-catalytic analysis in **Table S1** indicates that the best OER catalysts of Ni_3Co_1-CPs exhibit a ca. 1:1 Ni/Co atomic ratio after the OER; therefore, the following mechanistic discussion will be only focused on the spinel-type NiCo-oxide reference.)

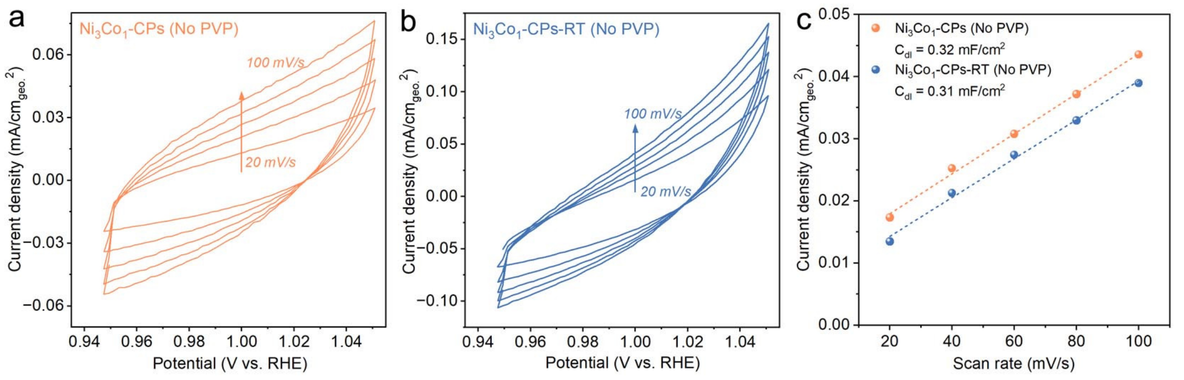

Figure S24. (a, b) CV curves for Ni_3Co_1-CPs (No PVP) and Ni_3Co_1-CPs-RT (No PVP). (c) Calculated C_{dl} values of two investigated catalysts.

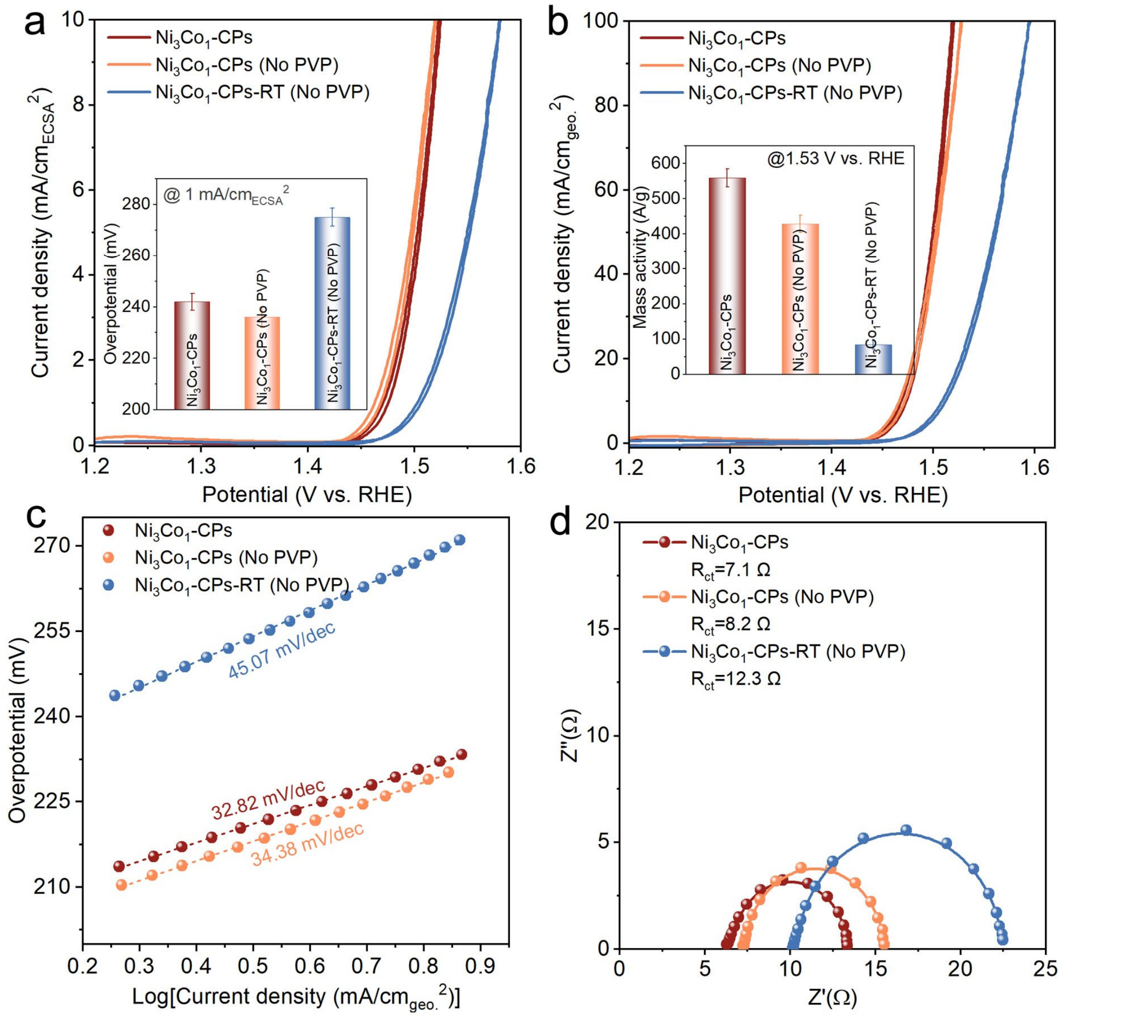

Figure S25. Electrocatalytic OER performance of Ni_3Co_1-CPs with different synthetic parameters in 1 M KOH (pH ~ 13.7, scan rate = 5 mV/s, mass loading = 0.25 mg/cm^2, and rotation speed = 1600 rpm): (a) 90% iR-corrected normalized CV curves; (b) 90% iR-corrected CV curves; (c) Tafel plots; (d) Nyquist plots (at 1.5 V vs. RHE).

Electrochemical characterizations of Ni_3Co_1-CPs (No PVP) and Ni_3Co_1-CPs-RT (No PVP) were performed to explore the influence of morphology and crystal structure effects on the OER performance. Our results (**Figure S25a**) show that Ni_3Co_1-CPs (No PVP) exhibit a lower overpotential at 1 mA/cm$_{ECSA}^2$ than Ni_3Co_1-CPs. However, mass activity analysis demonstrates that excluding PVP as a synthesis additive results in a decreased activity (**Figure S25b**). It should also be noted that Ni_3Co_1-CPs (No PVP) possesses a slightly lower Ni/Co atomic ratio

(2.48:1) relative to Ni_3Co_1-CPs (3.07:1) (**Table S1**). Thus, the minor differences in the OER performance between Ni_3Co_1-CPs (No PVP) and Ni_3Co_1-CPs are mainly attributed to variations in Ni/Co stoichiometry, rather than morphological effects. It is also noteworthy that Ni_3Co_1-CPs-RT (No PVP) displays poor OER performance compared to both counterparts, highlighting the crucial role of employing low-crystalline materials in promoting the O_2 generation.

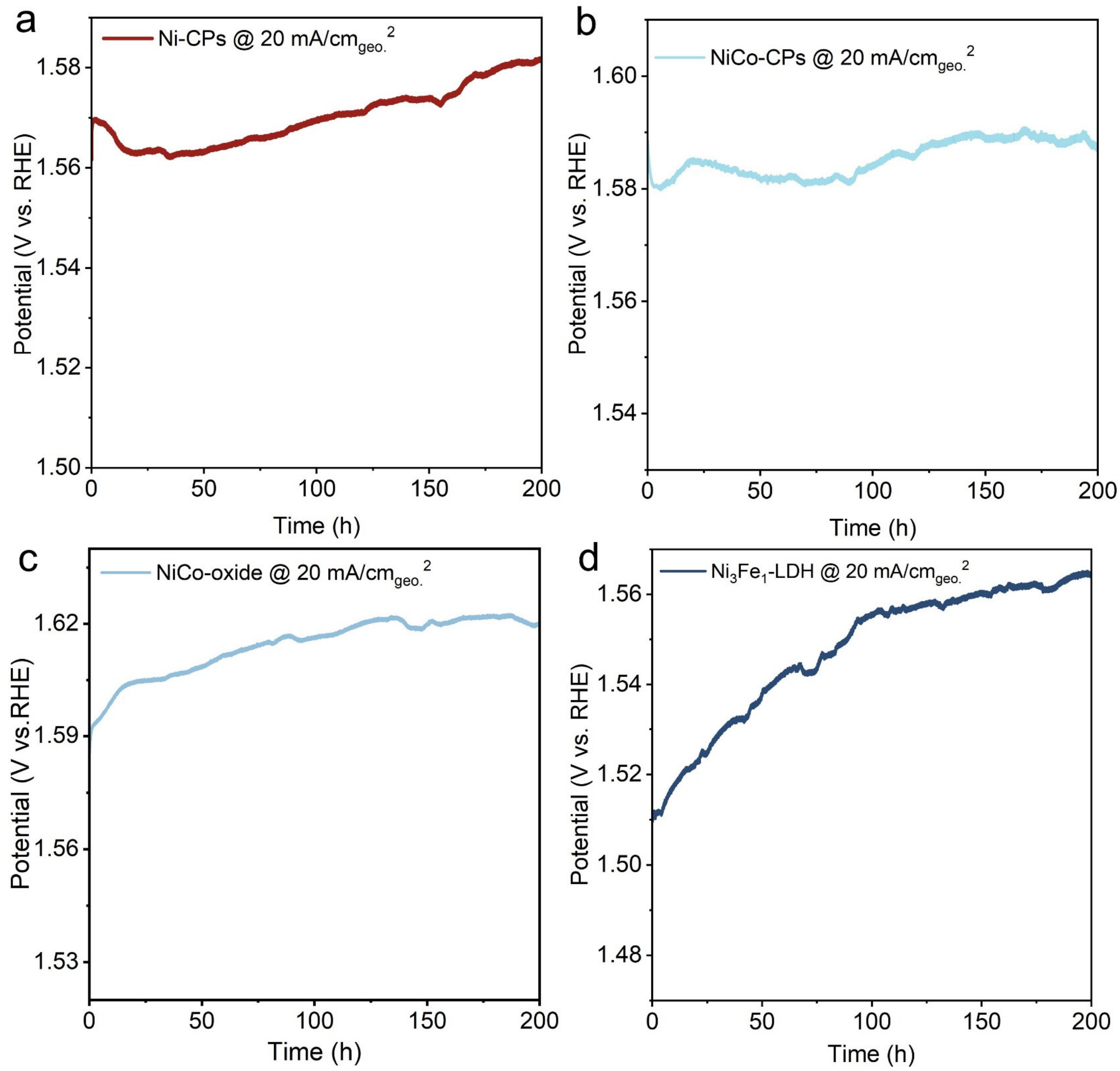

Figure S26. Chronopotentiometry tests of as-investigated catalysts for the OER: (a) Ni-CPs; (b) NiCo-CPs; (c) NiCo-oxide; (d) Ni_3Fe_1-LDH. (**Note**: catalysts were loaded on carbon paper. Recent studies have reported that atomistic crystal phase or metal center segregation emerges as a considerable factor in influencing both the catalytic activity and stability of the Fe-containing OER catalysts.[10-12] Accordingly, the observed instability behaviour of Ni_3Fe_1-LDH originates from electrochemically induced restructuring into Fe-containing phases with comparatively lower intrinsic OER activity.)

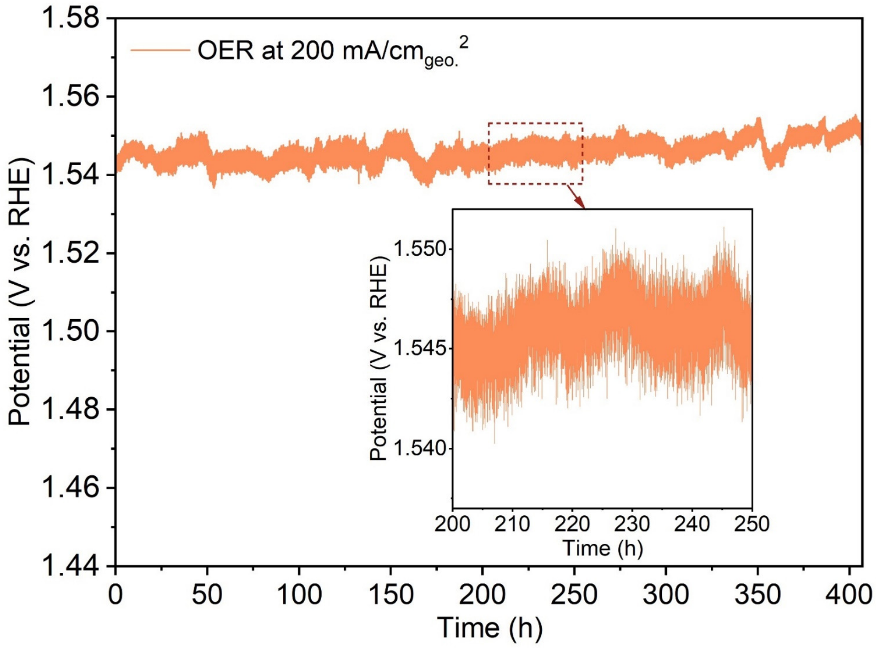

Figure S27. Chronopotentiometry tests of Ni_3Co_1-CPs. (**Note**: catalysts were loaded on carbon paper.)

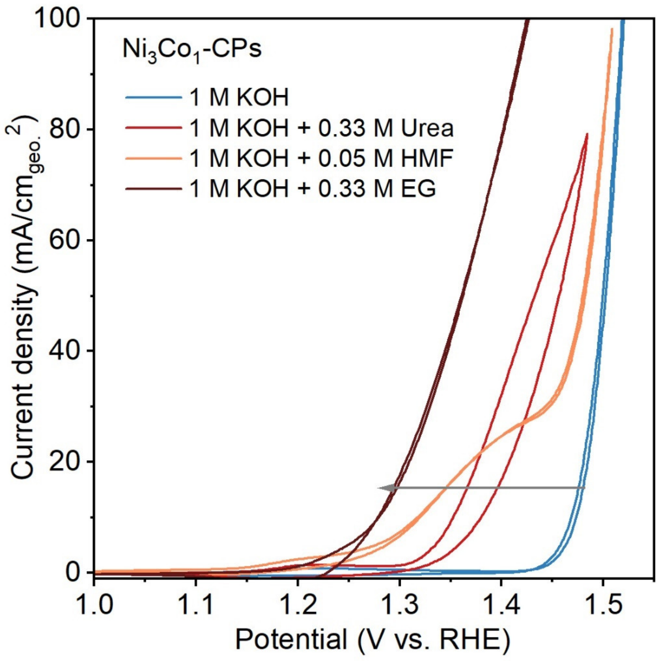

Figure S28. CV curves of Ni_3Co_1-CPs recorded in different reaction conditions.

3. Post-catalytic characterizations

3.1. Structural and morphological characterizations of the as-prepared catalysts after OER stability measurements

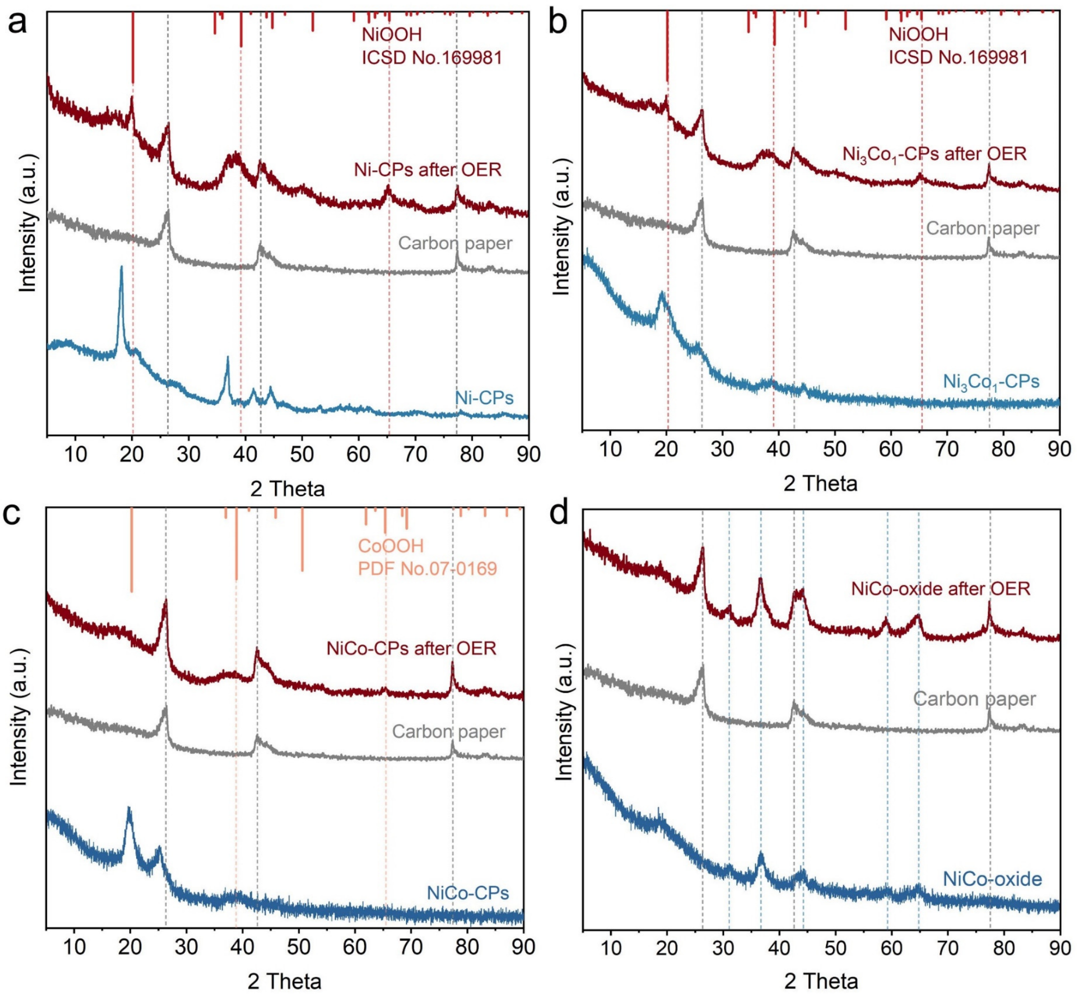

Figure S29. PXRD patterns of as-investigated catalysts before and after OER stability tests: (a) Ni-CPs; (b) Ni_3Co_1-CPs; (c) NiCo-CPs; (d) NiCo-oxide.

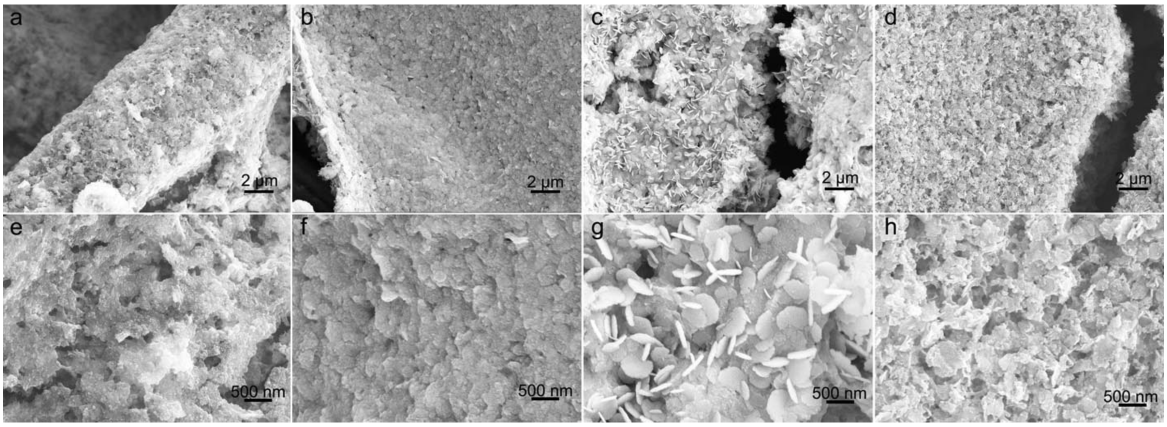

Figure S30. FESEM images of as-investigated catalysts after OER stability tests: (a, e) Ni-CPs; (b, f) Ni_3Co_1-CPs; (c, g) NiCo-CPs; (d, h) NiCo-oxide.

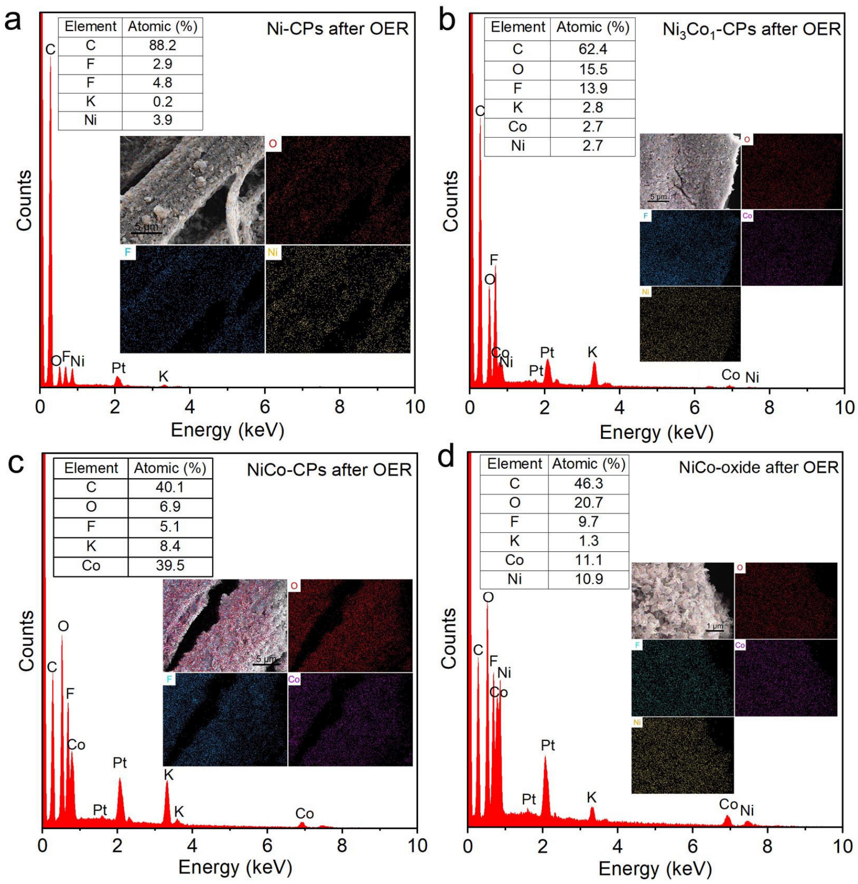

Figure S31. FESEM-EDX spectrum and element mapping images of as-investigated catalysts after OER stability tests: (a) Ni-CPs; (b) Ni_3Co_1-CPs; (c) NiCo-CPs; (d) NiCo-oxide. (**Note**: signals of F and K arise from Nafion and KOH, respectively.)

3.2. XPS characterization of the as-investigated catalysts before and after OER stability measurements

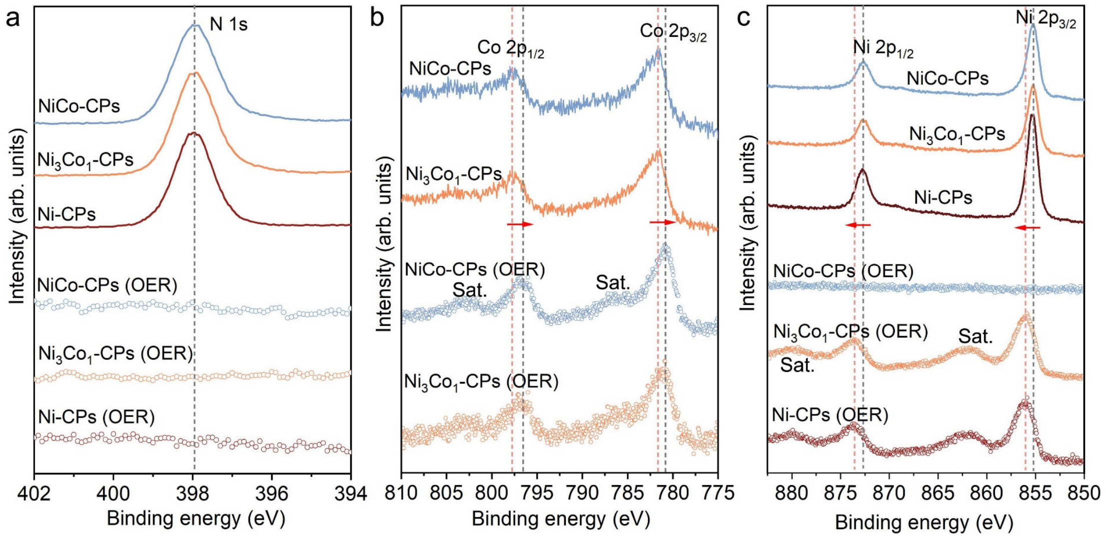

Figure S32. XP spectra of the three catalysts before and after OER stability tests: (a) N 1*s*; (b) Co 2*p*; (c) Ni 2*p*.

3.3. XAS characterization of Ni-CPs after OER stability measurements

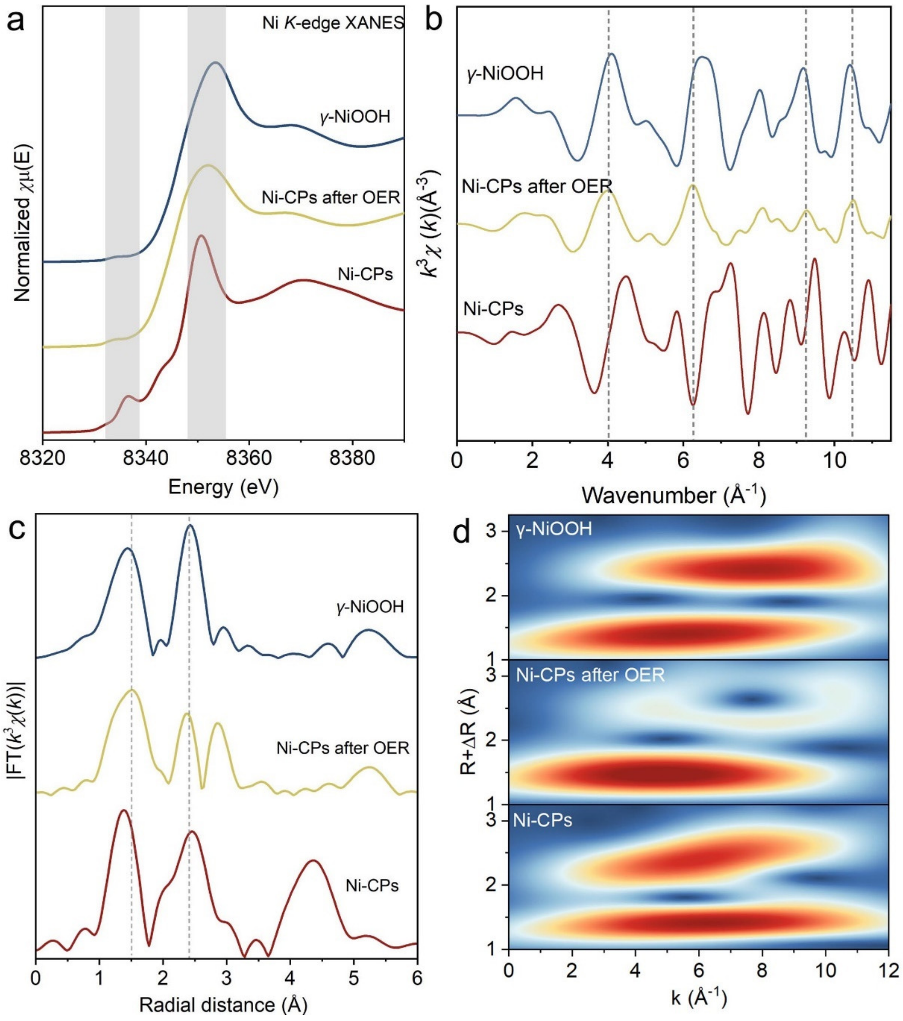

Figure S33. Co *K*-edge XAS characterizations of Ni-CPs before and after OER stability measurements: (a) XANES spectra; (b) EXAFS spectra; (c) FT-EXAFS spectra; (d) WT contour profiles.

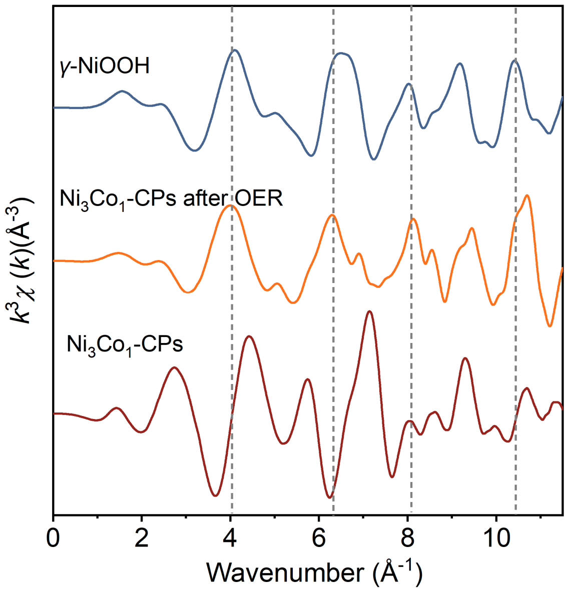

Figure S34. Ni *K*-edge EXAFS spectra of Ni_3Co_1-CPs before and after OER stability measurements.

3.4. XAS characterization of Ni_3Co_1-CPs after OER stability measurements

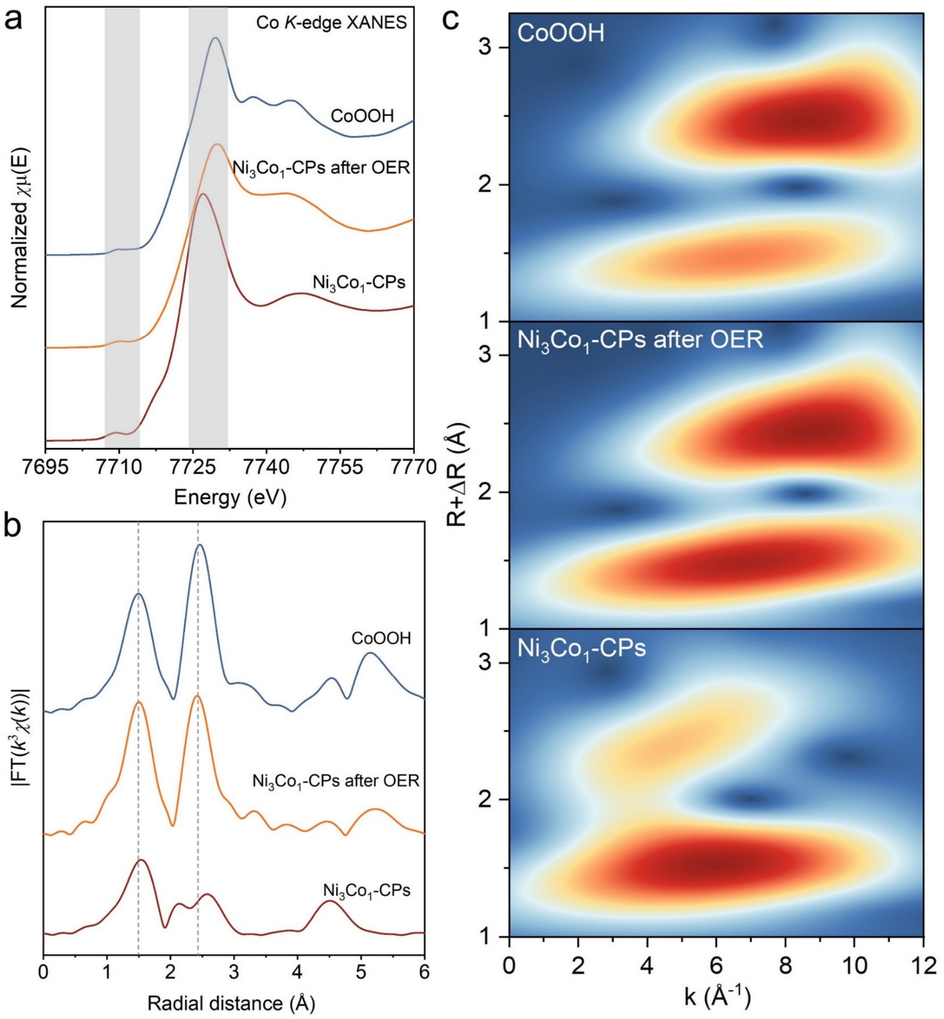

Figure S35. Co *K*-edge XAS characterizations of as-investigated Ni_3Co_1-CP catalysts before and after OER stability tests: (a) XANES spectra; (b) FT-EXAFS spectra; (c) WT contour profiles.

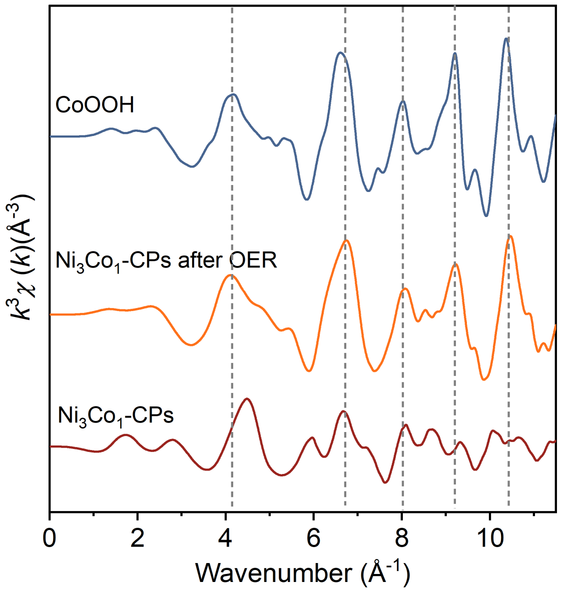

Figure S36. Co *K*-edge EXAFS spectra of Ni_3Co_1-CPs before and after OER stability measurements.

3.5. XAS characterization of NiCo-CPs after OER stability measurements

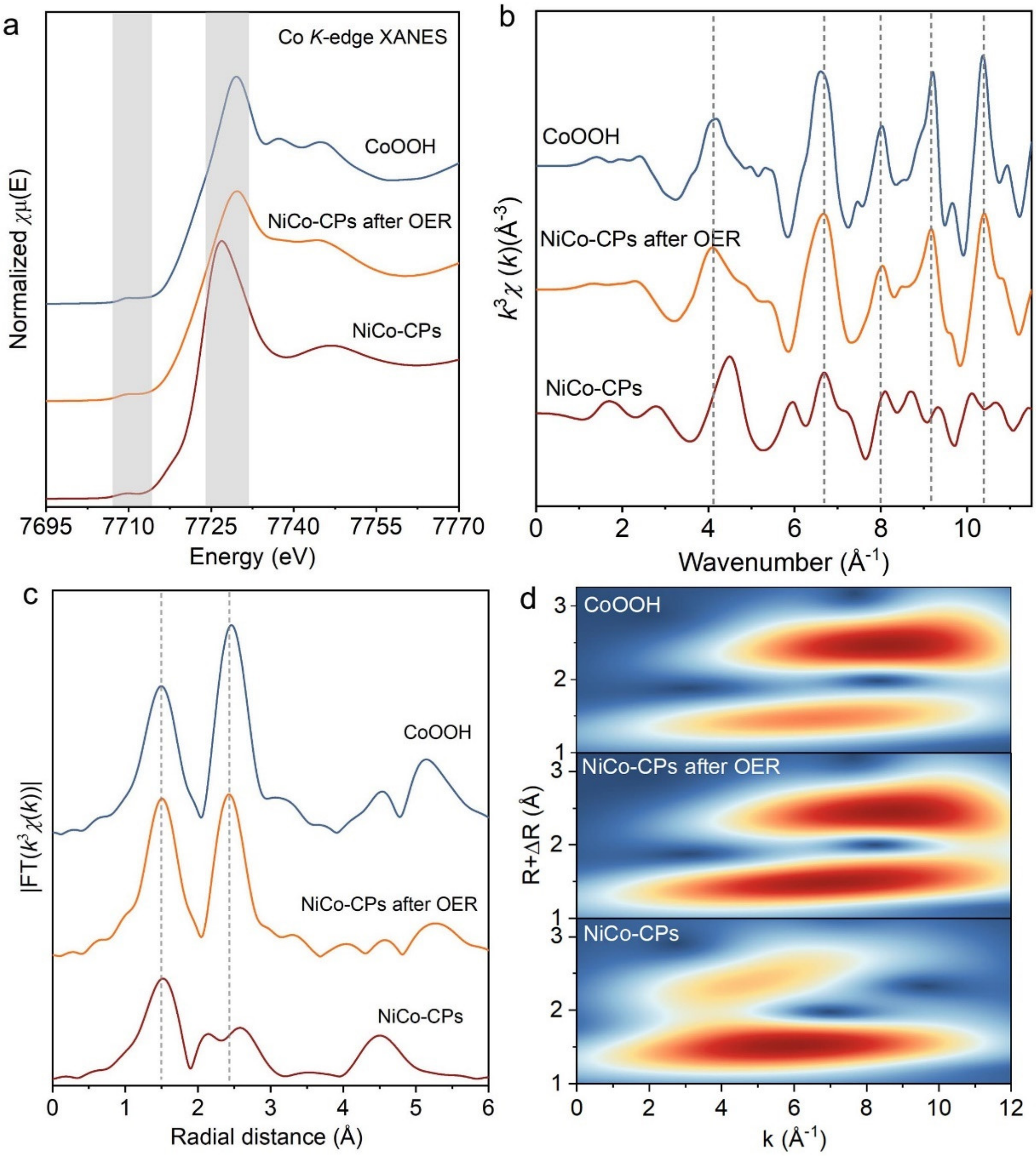

Figure S37. Co *K*-edge XAS characterizations of NiCo-CPs before and after OER stability measurements: (a) XANES spectra; (b) EXAFS spectra; (c) FT-EXAFS spectra; (d) WT contour profiles.

3.6. XAS characterization of NiCo-oxide after OER stability measurements

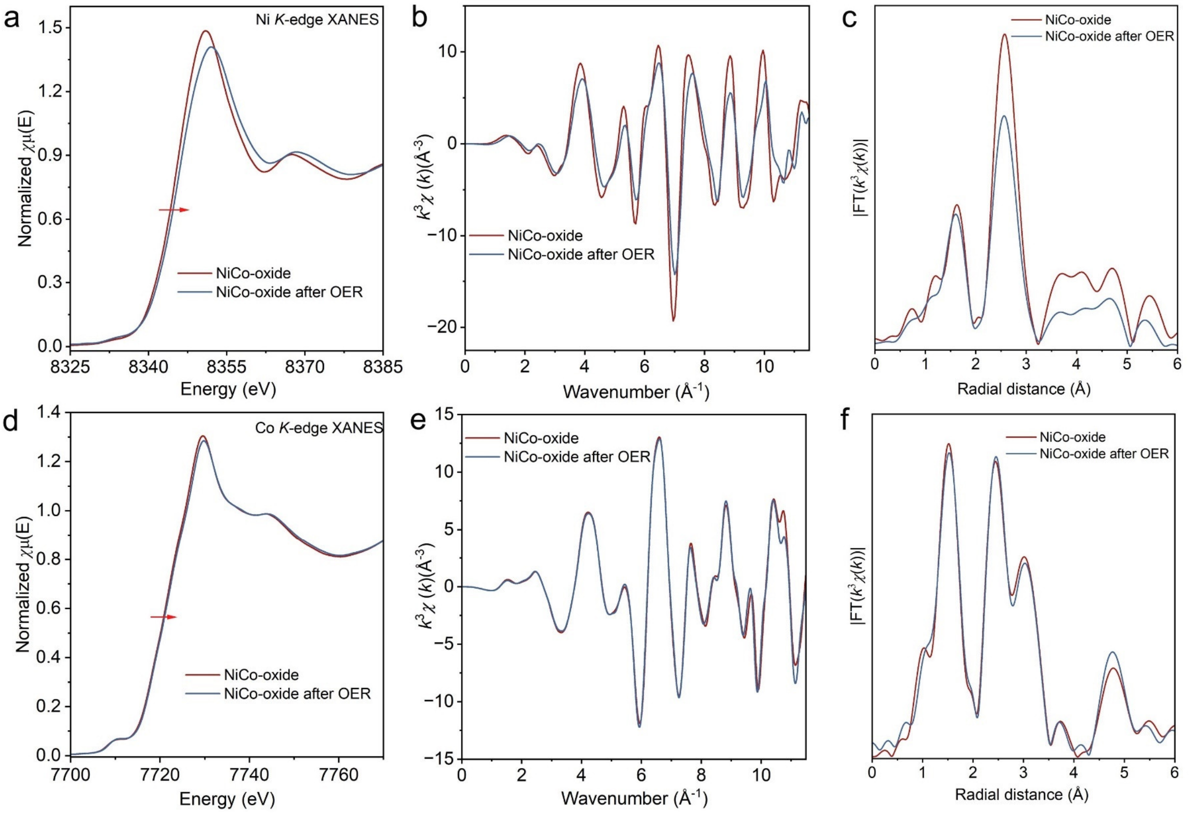

Figure S38. Ni and Co *K*-edge XAS characterizations of NiCo-oxide before and after OER stability measurements: (a, d) XANES spectra; (b, e) EXAFS spectra; (c, f) FT-EXAFS spectra.

4. *Operando* XAS characterizations of the as-investigated catalysts for the OER

4.1. *Operando* XAS characterizations of Ni-CPs for the OER

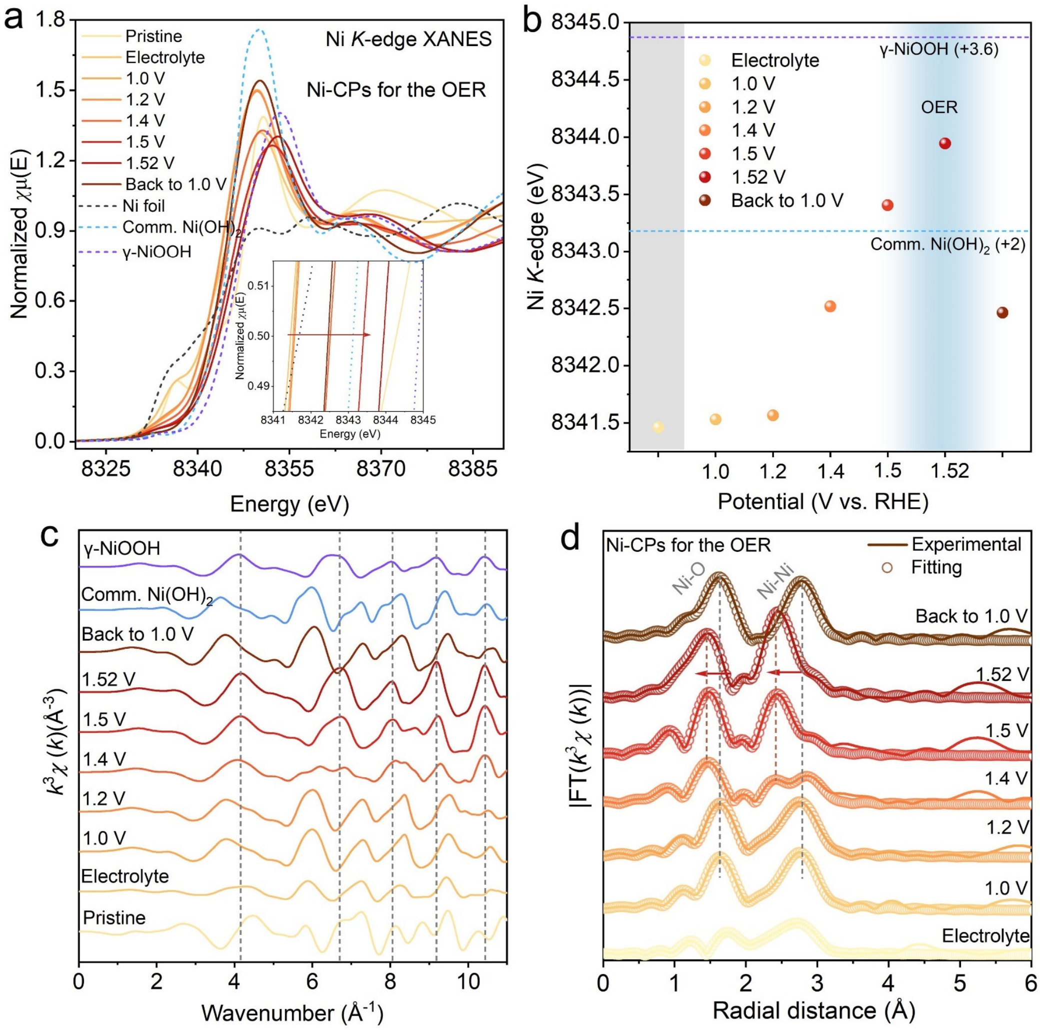

Figure S39. *Operando* Ni *K*-edge XAS characterizations of Ni-CPs for the OER: (a) XANES spectra; (b) Ni *K*-edge positions ($F/I_0 = 0.5$) as a function of the applied potentials; (c) EXAFS spectra; (e) Fitting of FT-EXAFS spectra.

Operando Ni *K*-edge XAS characterizations were conducted to gain mechanistic insights into the dynamic evolution of active species and sites in Ni-CPs during the OER. The *operando* Ni *K*-edge XANES spectra (**Figure S39a**) indicate that the spectral features associated with the Ni(II)X_4 geometry undergo a substantial profile change as a function of the applied potential and completely vanish in the OER region. This points to an underlying local structural optimization occurring at the Ni centers in Ni-CPs during the OER. Analysis of the Ni *K*-edge position reveals a distinct positive energy shift, with a maximum value of ca. 8343.9 eV at 1.52 V vs. RHE (**Figure S39b**), which is higher than that of $Ni(OH)_2$ (+2: 8343.2 eV) yet lower than γ-NiOOH (+3.6: 8344.9 eV). This points out that the Ni(II) centers in Ni-CPs undergo oxidation into the high-valent Ni(IV) sites that act as the true catalytically active sites to trigger the OER. As expected, upon switching the applied potential back to 1.0 vs. RHE, a reversible conversion from the catalytically active Ni (IV) species into the OER resting-state Ni(II/III) species is observed.

The time-dependent changes of local coordination environments of Ni centers in Ni-CPs were further evaluated from *operando* Ni *K*-edge EXAFS spectra analyses. As shown in **Figure S39c**, after immersing into the electrolyte, the characteristic EXAFS oscillations of Ni-CPs display a pronounced profile change; with the anodic polarizations, these oscillation features change their profile shape and resemble γ-NiOOH within the OER potential region; when the applied potential was reversed to 1.0 V vs. RHE, the characteristic oscillation profiles exhibit similar spectral features as to $Ni(OH)_2$, indicating the redox reversibility of Ni centers during the OER. To precisely unveil the structural dynamics at the Ni centers, *operando* Ni *K*-edge FT-EXAFS spectra analyses were further performed. From the fitting results of the *operando* Ni *K*-edge FT-EXAFS spectra (**Figure S39d and Table S6**), we observe that Ni-CPs experience a distinct change in the local coordination environment of Ni centers during the OER, as evident from a decrease in $CN_{Ni\text{-}C}$ and $CN_{Ni\text{-}N/C\text{-}N}$ and an increase in $CN_{Ni\text{-}O}$ and $CN_{Ni\text{-}Ni}$. Moreover, during the OER, the newly appeared Ni-O and Ni-Ni backscattering signatures feature a decreasing tendency toward shorter interatomic distance, reaching a minimum value at 1.52 V vs. RHE. After the applied potential returns to 1.0 V vs. RHE, a noticeable increase in $R_{Ni\text{-}O}$ and $R_{Ni\text{-}Ni}$ is observed, consistent with the regeneration of the OER resting species.

Taken together, we propose that the investigated Ni-CPs undergo a dynamic restructuring into catalytically active high-valent Ni(IV) species, which serve as the true OER active species. A schematic overview of the aforementioned structural evolution is presented in **Figure S40**: (i) after the catalysts are immersed into the electrolyte, an underlying anion exchange reaction occurs between $[Ni(CN)_4]^{2-}$ and OH^-, partially converting the coordination environment to OH^- containing Ni(II) species, as supported by XAS and ICP-MS analyses (**Tables S5 and S6**); (ii) with the anodic polarization from 1.0 to 1.2 V vs. RHE, further anion exchange reaction promotes the formation of $Ni(OH)_2$-like species, in which the Ni centers remain as +2 (**Figure S39b and Table S6**); (iii) a continuous increase in the applied potential up to 1.52 V triggers the deprotonation of Ni(II)-OH species into Ni(IV)-O species with the generation of Ni(III)-OH intermediates; (iv) upon returning to 1.0 V, these *in situ*-formed Ni(IV) species revert to the Ni(II/III) resting states. This observation about the formation of the catalytically active Ni(IV) species in Ni-CPs for the OER is further corroborated by *operando* Raman analyses (cf. following section in **Figure S48**).

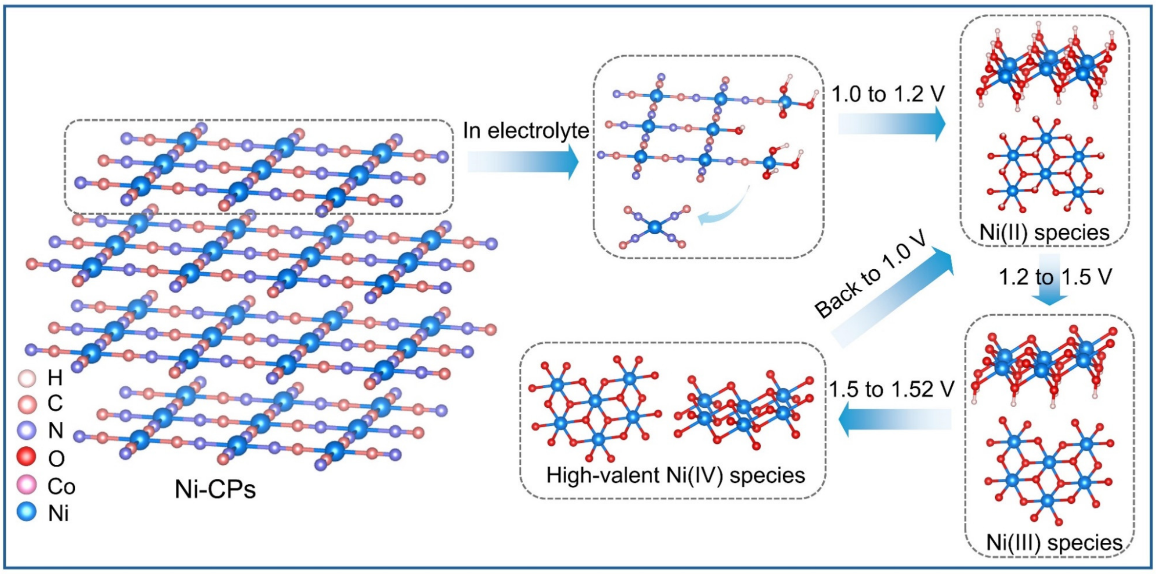

Figure S40. Schematic illustration of the structural evaluation process of Ni-CPs during the OER.

4.2. *Operando* quick-XAS characterizations of Ni_3Co_1-CPs for the OER

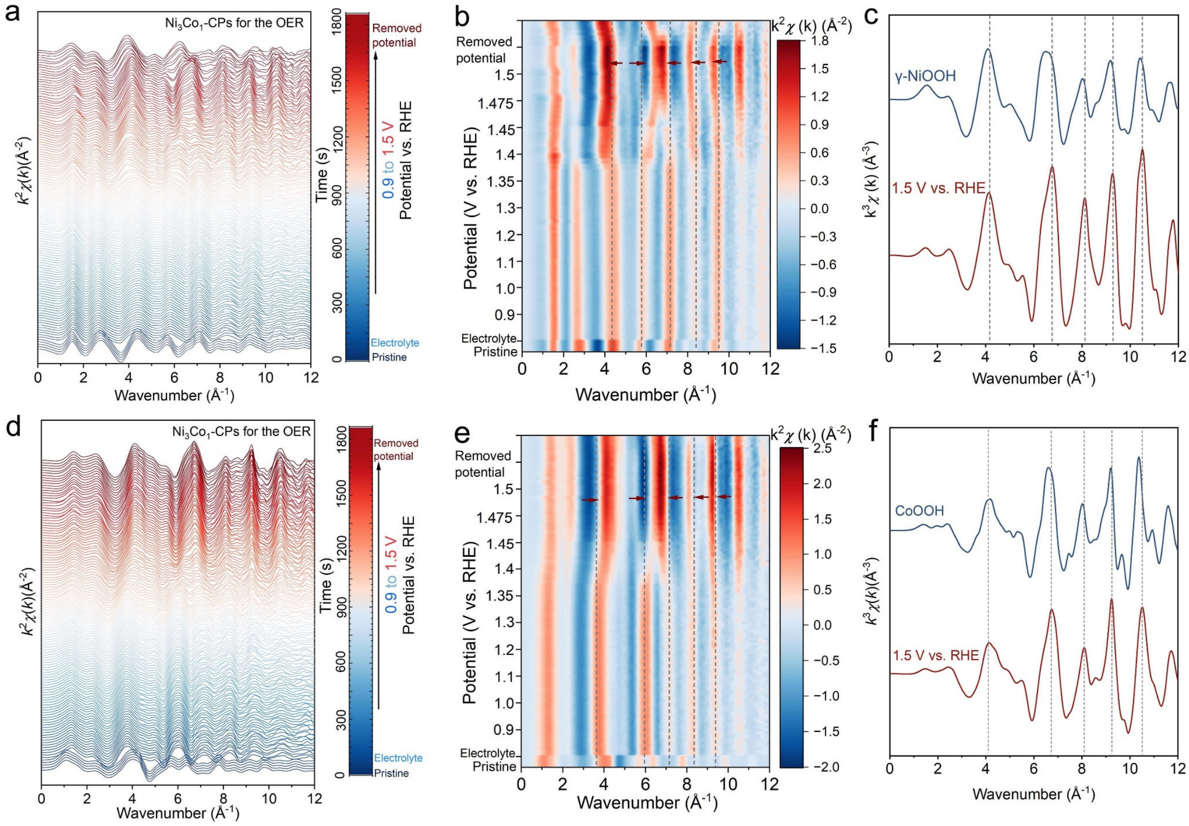

Figure S41. *Operando* time-resolved Ni (a) and Co (d) *K*-edge EXAFS spectra of Ni_3Co_1-CPs for the OER. 2D contour plots of time-resolved Ni(b) and Co(e) *K*-edge EXAFS spectra of Ni_3Co_1-CPs for the OER. Comparison of Ni (c) and Co (f) *K*-edge EXAFS spectra of Ni_3Co_1-CPs recorded at 1.52 V vs. RHE vs. references.

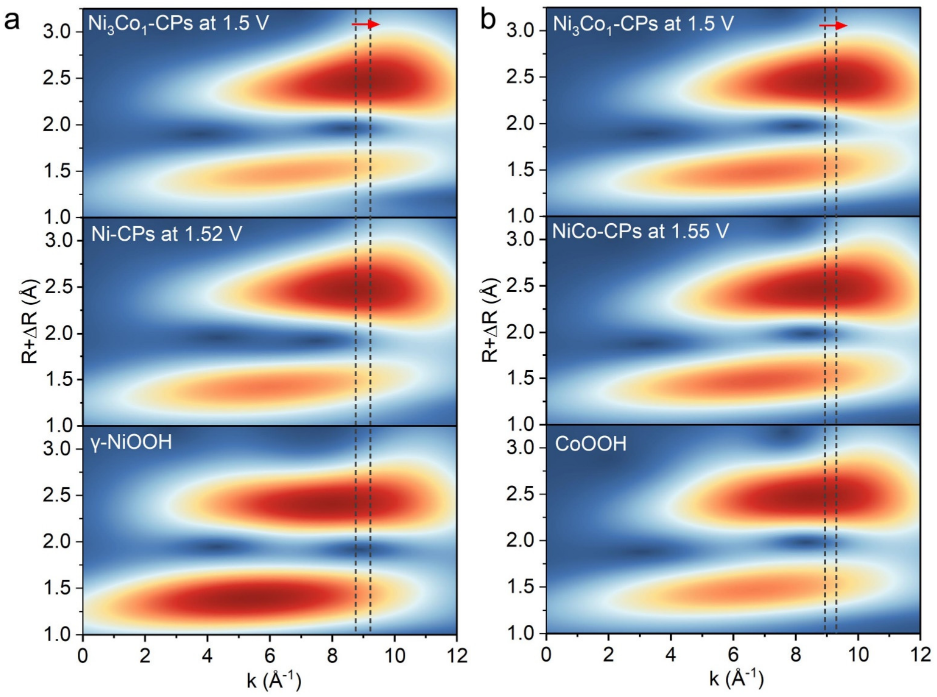

Figure S42. (a, b) Ni and Co *K*-edge WT contour profiles of the investigated catalysts.

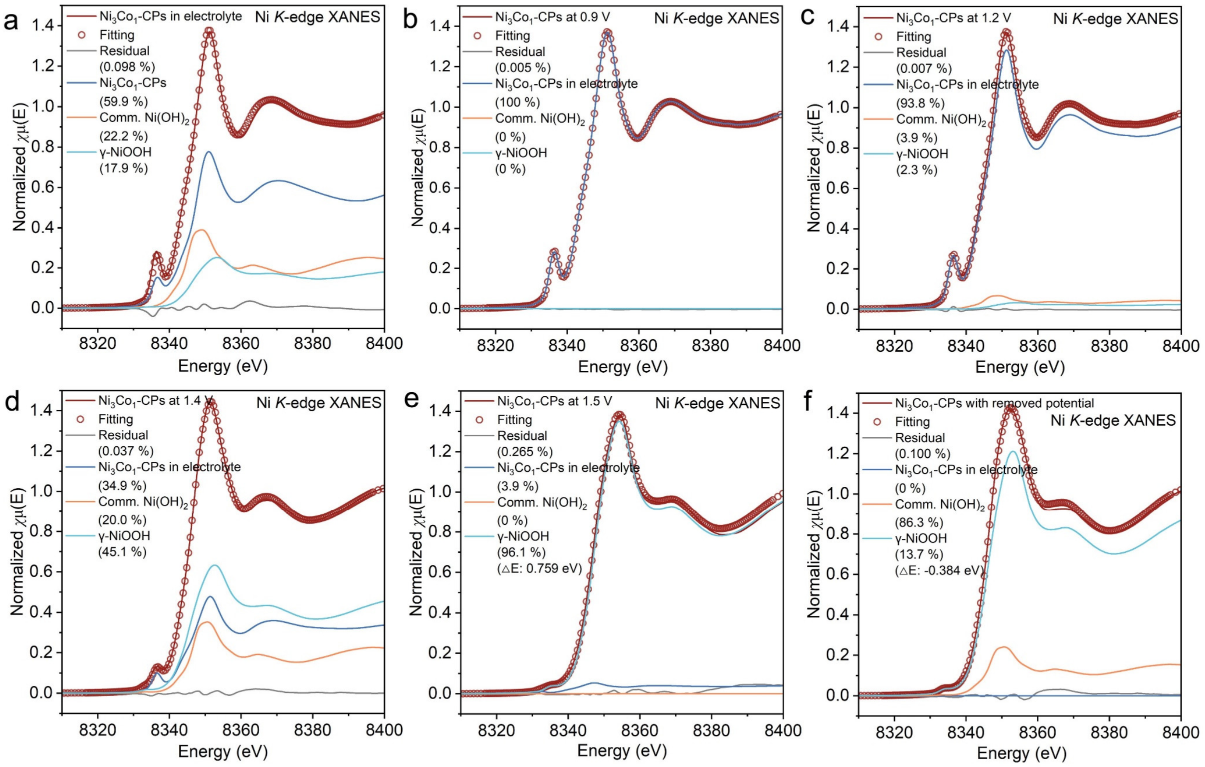

Figure S43. LCF analysis of Ni *K*-edge XANES spectra of Ni_3Co_1-CPs for the OER: (a) in electrolyte; (b) 0.9 V vs. RHE; (c) 1.2 V vs. RHE; (d) 1.4 V vs. RHE; (e) 1.5 V vs. RHE; (f) removed potential.

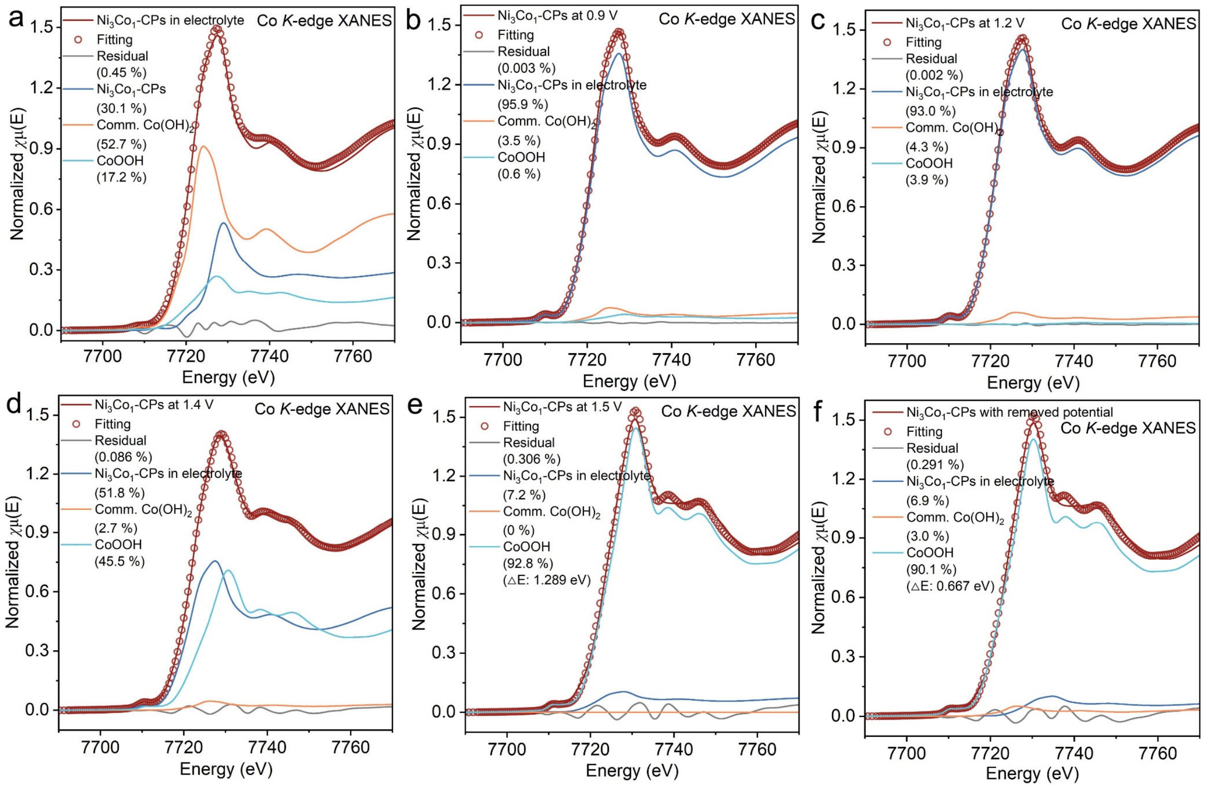

Figure S44. LCF analysis of Co *K*-edge XANES spectra of Ni_3Co_1-CPs for the OER: (a) in electrolyte; (b) 0.9 V vs. RHE; (c) 1.2 V vs. RHE; (d) 1.4 V vs. RHE; (e) 1.5 V vs. RHE; (f) removed potential.

The impact of Co incorporation on the dynamic evolution of local coordination environments of Ni centers was further evaluated using *operando* time-resolved Ni and Co *K*-edge EXAFS spectra analyses. As shown in **Figure S41a,b**, the Ni *K*-edge EXAFS spectra of pristine Ni_3Co_1-CPs feature 6 prominent oscillation signatures at k values of ca. 1.55, 2.75, 4.35, 5.65, 7.15, and 7.35 $Å^{-1}$, of which exhibit only slight intensity variations from immersing into the electrolyte to 1.4 V vs. RHE. Additionally, we also observed one newly emergent oscillation feature at a k value of ca. 8.35 $Å^{-1}$, and it remains almost identical within the applied potential ranges from 0.9 to 1.4 V vs. RHE. In sharp contrast, the Co *K*-edge EXAFS spectra of Ni_3Co_1-CPs (**Figure S41d,e**) show that the initial characteristic oscillation signatures completely vanish upon immersion of the catalysts into the electrolyte. Meanwhile, six newly formed oscillation signatures located at k values of ca. 1.35, 3.75, 6.05, 7.15, 8.35, and 9.35 $Å^{-1}$ are observed, and their spectral profiles remain almost identical under the anodic polarization up to 1.35 V vs. RHE. When the applied potentials are higher than 1.4 V (for Ni *K*-edge) and 1.35 V (for Co *K*-edge), the corresponding *operando* Ni and Co *K*-edge EXAFS spectra (**Figure S41c,f**) feature oscillation features that resemble those of γ-NiOOH and CoOOH references, respectively. These observations suggest that under the OER conditions Ni_3Co_1-CPs undergo drastic local structural geometry evolution to generate the catalytically active high-valent (Ni, Co) (IV) species, which serve as the true OER active species. To further strengthen our key findings on the presence of Ni^{IV}-O-Co^{IV} moieties during the OER, we performed additional WT analysis for Ni-CPs, Ni_3Co_1-CPs, and NiCo-CPs during the OER. As shown in **Figure S42**, Ni-CPs and NiCo-CPs feature their intensity maximum at ca. 8.75 and 8.94 $Å^{-1}$, respectively, attributed to the high-valent $Ni^{III/IV}$-O-Ni^{IV} and $Co^{III/IV}$-O-Co^{IV}. In comparison, the intensity maximum at both Ni and Co *K*-edge WT contour profiles of Ni_3Co_1-CPs undergoes a significant wavenumber shift to higher k values of 9.22 and 9.27 $Å^{-1}$, respectively, inferring the formation of Ni^{IV}-O-Co^{IV} moieties during the OER. Such a construction of key Ni^{IV}-O-Co^{IV} moieties for the OER can also be supported by our *operando* Raman characterizations (**Figure 5a and Figure S48**).

To quantitatively explore the phase evolution of Ni_3Co_1-CPs during the OER, linear combination fitting (LCF) analysis of the *operando* Ni and Co *K*-edge XANES spectra was conducted (**Figures S43 and S44**). Notably, for the samples immersed into the electrolyte, precise determination of their phase composition is challenging due to electrolyte interference with the catalyst's surface. Therefore, pristine Ni_3Co_1-CPs, commercial $Ni(OH)_2$/$Co(OH)_2$, and γ-NiOOH/CoOOH were employed as reference standards for fitting of the Ni/Co *K*-edge XANES spectra. For the samples under applied potentials, the pristine Ni_3Co_1-CPs reference was replaced by the sample immersed into the electrolyte. From the Ni *K*-edge LCF results (**Figure S43a**), after immersion into the electrolyte, the Ni_3Co_1-CPs content decreases to ca. 59.9%, accompanied by the presence of $Ni(OH)_2$ (ca. 22.2 %) and γ-NiOOH (ca. 17.9 %), which arise from the underlying exchange reaction process between $[Ni(CN)_4]^{2-}$ and OH^-. In sharp contrast, analysis of Co *K*-edge XANES spectra (**Figure S44a**) shows that only ca. 30.1% of Ni_3Co_1-CPs remains under the same conditions. This indicates that Ni_3Co_1-CPs undergo a distinct electrolyte-dependent surface restructuring at Ni and Co centers, in which the underlying restructuring preferentially occurs at the Co centers. These results are consistent with our *operando* Ni and Co *K*-edge XANES monitoring for Ni_3Co_1-CPs (**see detailed discussion in Figure 4b,f**). With the anodic polarizations, both Ni and Co centers undergo multi-step phase transformations from pristine CPs into (oxy)hydroxides via the formation of hydroxide intermediates (**Figures S43b-e and S44b-e**). When the applied potential reaches 1.5 V vs. RHE, nearly all Ni_3Co_1-CPs convert into (oxy)hydroxide-related species, yielding ca.

96.9 % of γ-NiOOH at the Ni *K*-edge (**Figure S43e**) and ca. 92.8 % of CoOOH at the Co *K*-edge (**Figure S44e**), respectively. Caution needs to be taken that the LCF results also reveal a significant positive energy shift in the Ni and Co *K*-edge positions at 1.5 V vs. RHE (0.759 and 1.289 eV, respectively) compared with pristine γ-NiOOH and CoOOH. This suggests that these in situ-generated (Ni, Co) (oxy)hydroxides undergo a further deprotonation to generate high-valent active (Ni, Co)(IV) species for triggering the OER. After the removal of the applied potential, both Ni and Co *K*-edge XANES spectra display a decrease in γ-NiOOH (ca. 17.3 %) and CoOOH (90.1 %) content, along with the regeneration of $Ni(OH)_2$ (86.3 %) and $Co(OH)_2$ (3.0 %), while no significant content of pristine Ni_3Co_1-CPs is recovered (**Figures S43f and S44f**). These results are largely consistent with our discussions on operando XAS and Raman characterizations (**Figures 4 and 5a**), strongly supporting that Ni_3Co_1-CPs undergo irreversible restructuring into high-valent (Ni, Co)(IV) species that facilitate the oxygen evolution catalysis.

4.3. *Operando* XAS characterizations of NiCo-CPs for the OER

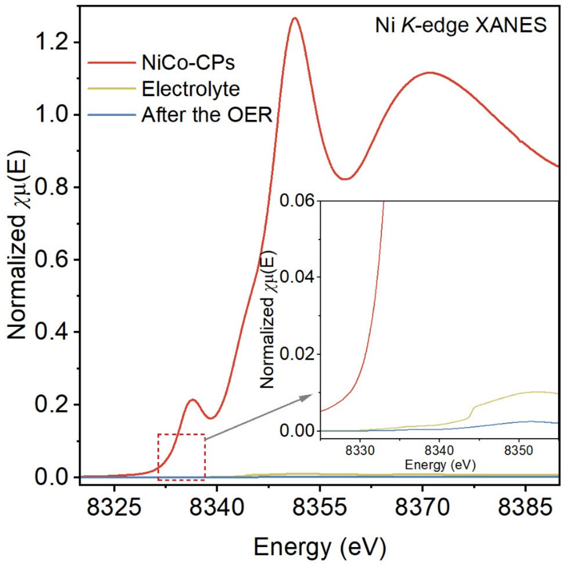

Figure S45. *Operando* Ni *K*-edge XAS characterizations of NiCo-CPs for the OER.

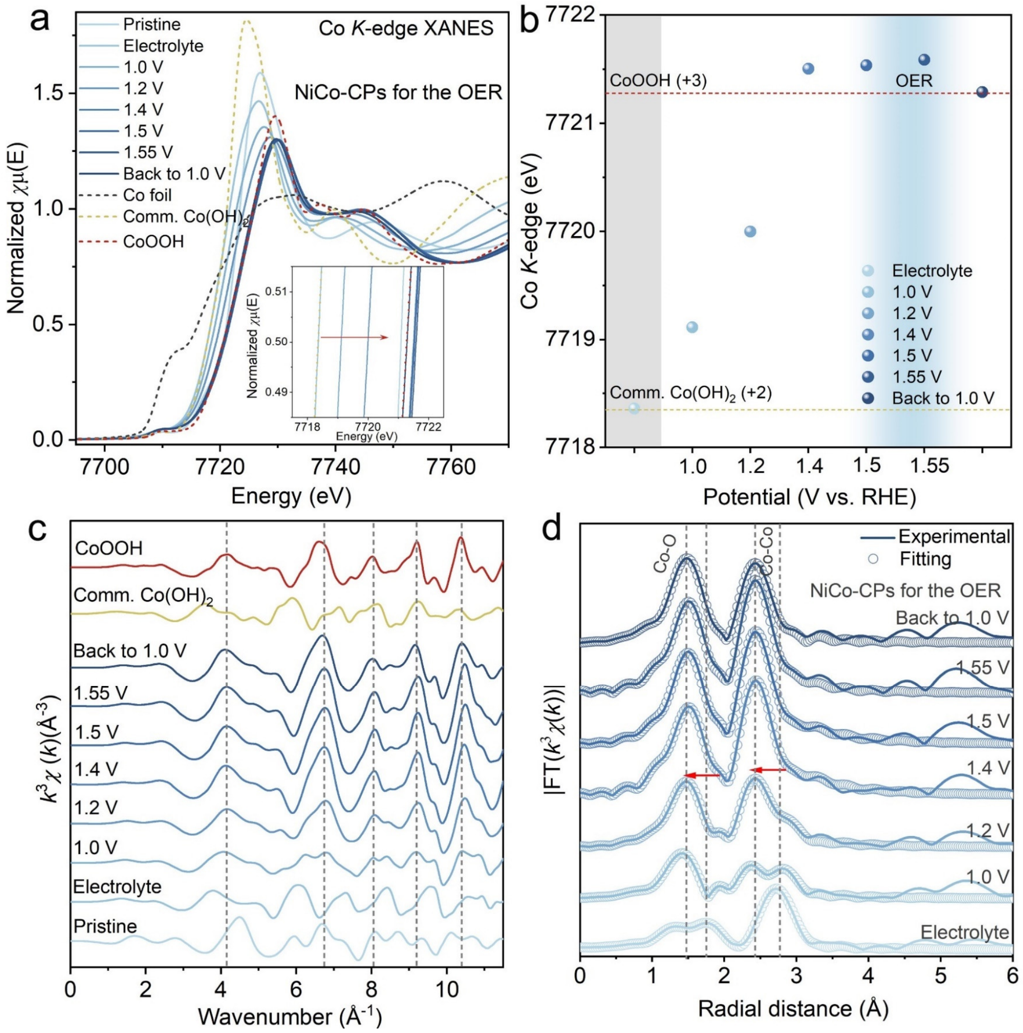

Figure S46. *Operando* Co *K*-edge XAS characterizations of NiCo-CPs for the OER: (a) XANES spectra; (b) Co *K*-edge positions ($F/I_0 = 0.5$) as a function of the applied potentials; (c) EXAFS spectra; (d) Fitting of FT-EXAFS spectra.

4.4. *Operando* XAS characterizations of NiCo-oxide for the OER

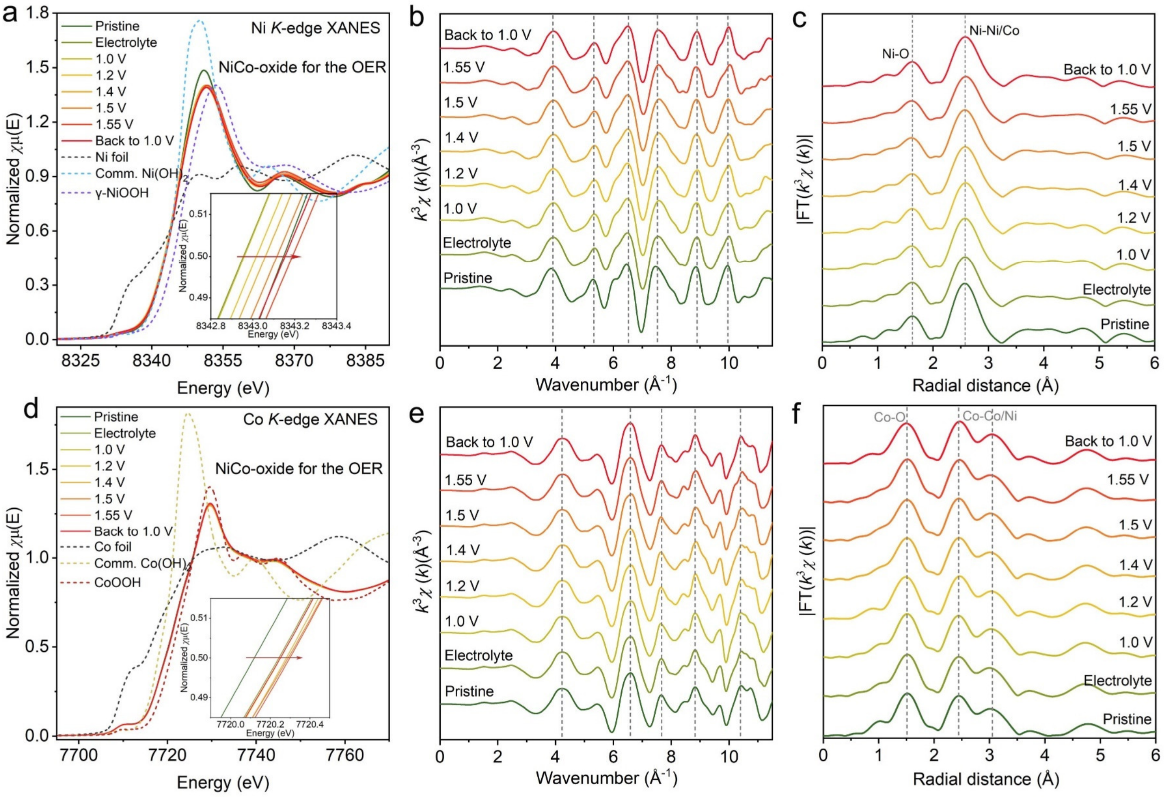

Figure S47. *Operando* Ni and Co *K*-edge XAS characterizations of NiCo-oxide for the OER: (a, d) XANES spectra; (b, e) EXAFS spectra; (c, f) FT-EXAFS spectra.

To further elucidate the origin of the intrinsic OER activity in the as-prepared Co-substituted Ni-CPs, *operando* XAS characterizations were carried out for the spinel-type NiCo-oxide reference. From the results (**Figure S47**), anodic polarization induces only a slight positive energy shift in both the Ni and Co *K*-edge positions, suggesting limited generation of high-valent Ni(IV) and Co(IV) species during the OER. Analysis of the *operando* Ni and Co *K*-edge XANES and FT-EXAFS spectra corroborates that the local coordination environments of Ni and Co centers in NiCo-oxides remain largely unchanged throughout the complete OER monitoring. These results confirm that oxide-type catalysts typically exhibit higher energy barriers for realizing restructuring into the catalytically active high-valent species during the OER.

5. *Operando* Raman characterizations of the as-investigated catalysts for the OER

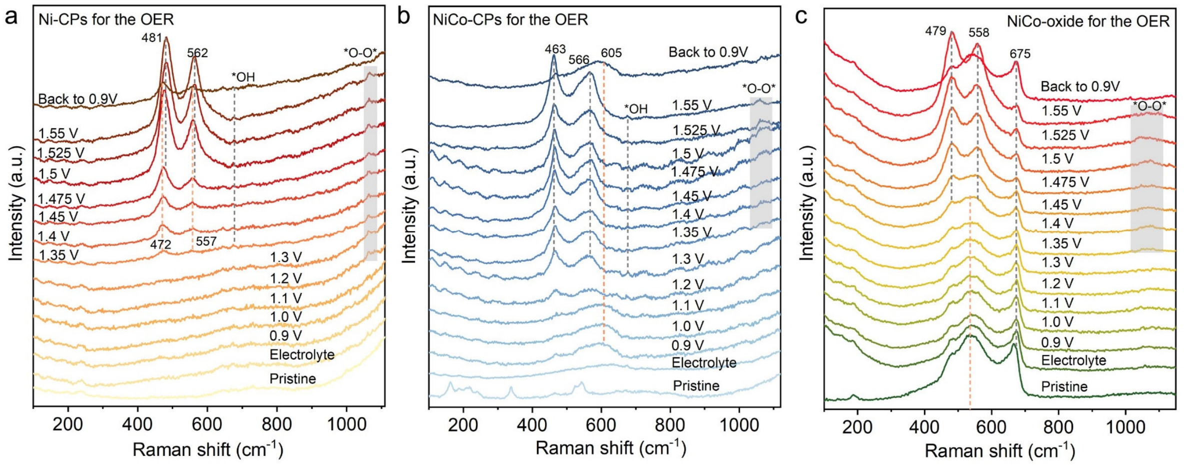

Figure S48. *Operando* Raman spectra as a function of the OER applied potentials: (a) Ni-CPs; (b) NiCo-CPs; (c) NiCo-oxide.

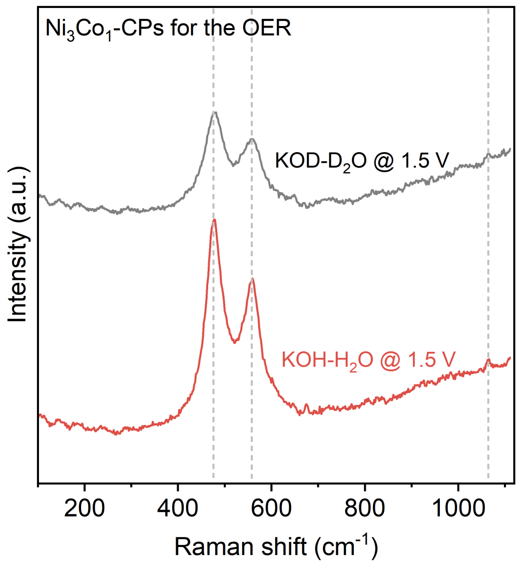

Figure S49. *Operando* Raman spectra of Ni_3Co_1-CPs recorded in different OER conditions.

6. ***Operando*** **EIS characterizations of the as-investigated catalysts for the OER**

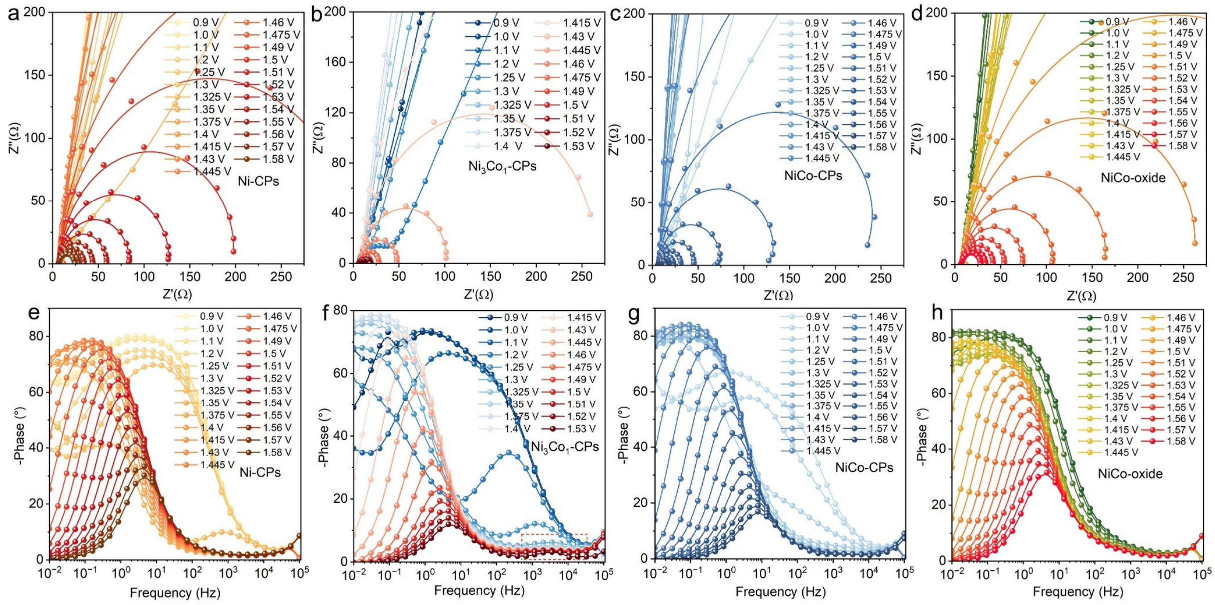

Figure S50. *Operando* EIS characterizations of four catalysts for the OER: (a-d) Nyquist plots; (e-h) Bode plots.

7. Pulse chronoamperometry measurements of the as-investigated catalysts

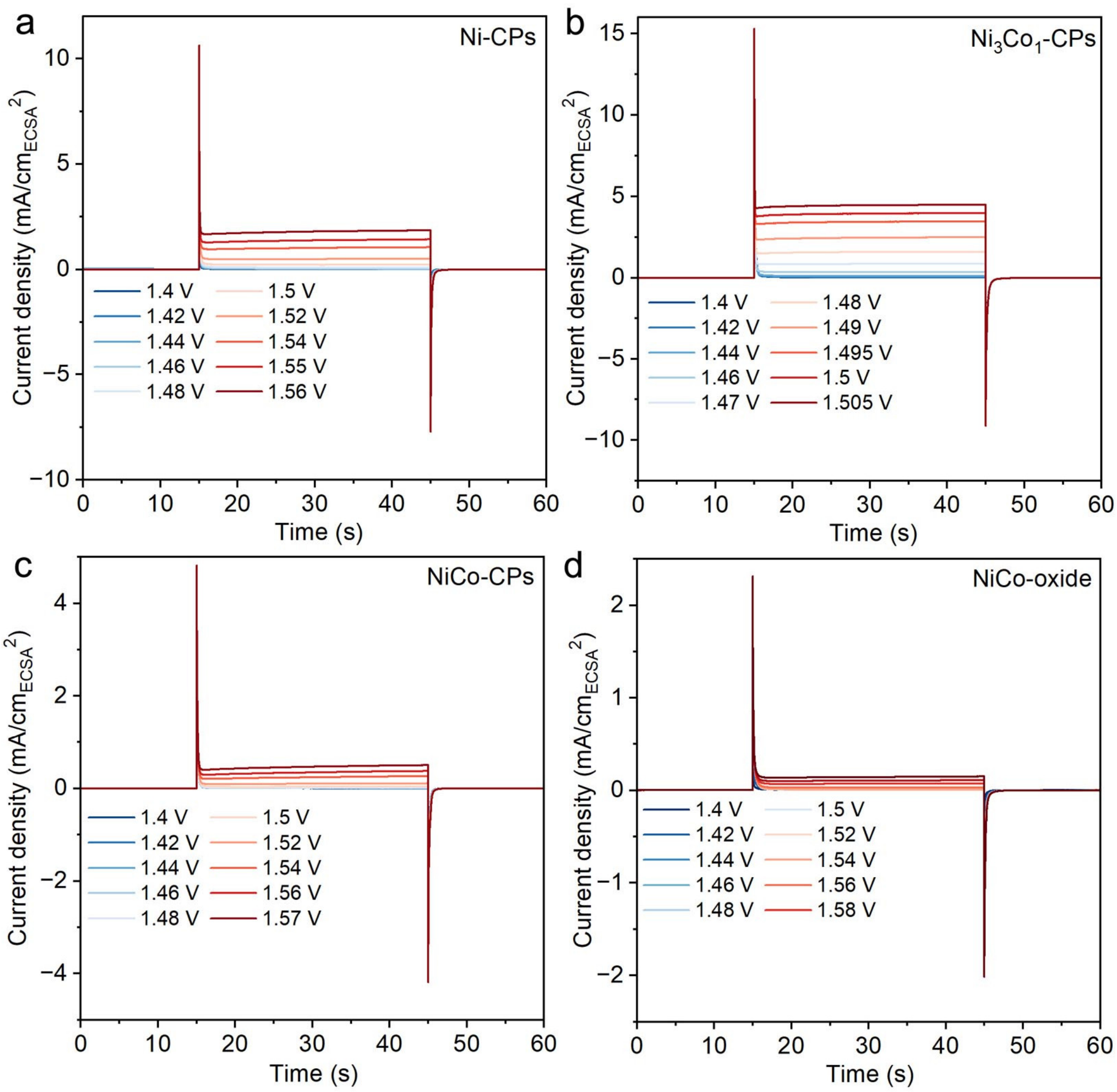

Figure S51. Pulse chronoamperometry characterizations of Ni-CPs (a), Ni_3Co_1-CPs (b), NiCo-CPs (c), and NiCo-oxide (d). (**Note:** During the measurements, the working electrode was held at the individual applied potential for 30 s and subsequently switched to 1.2 V vs. RHE. An interval time of 0.01 s was set to record the current signals. The total charge densities in **Figure 5b** were calculated through integration of the reduction current density after the applied potential was switched to 1.2 V vs. RHE.)

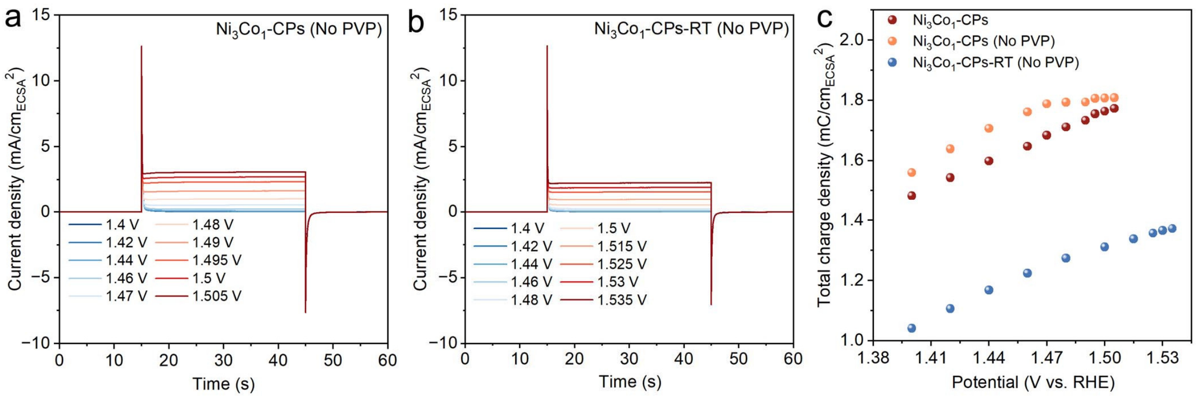

Figure S52. (a, b) Pulse chronoamperometry characterizations of Ni_3Co_1-CPs (No PVP) and Ni_3Co_1-CPs-RT (No PVP). (c) Calculated surface accumulated charge density of the three investigated catalysts.

Figure S52c shows the pulse chronoamperometry results of three investigated catalysts. From the results, as the applied potentials increase, the total charge densities stored in each catalyst increase accordingly. A close inspection reveals that Ni_3Co_1-CPs (No PVP) only exhibit a slightly higher charge density than Ni_3Co_1-CPs, reflecting a similar reaction kinetics involved during the OER, consistent with our above electrochemical characterizations (**detailed discussion in Figure S25**). As discussed in the main text, this minor difference originates from variations in the Ni/Co atomic ratios rather than morphology changes (**Table S1**). Notably, the accumulated total charge density of Ni_3Co_1-CPs (No PVP) is significantly higher than that of Ni_3Co_1-CPs-RT (No PVP), manifesting that the employment of low-crystalline CPs facilitates the formation of a high fraction of high-valent (Ni, Co)(IV) species.

8. pH-dependent OER characterizations

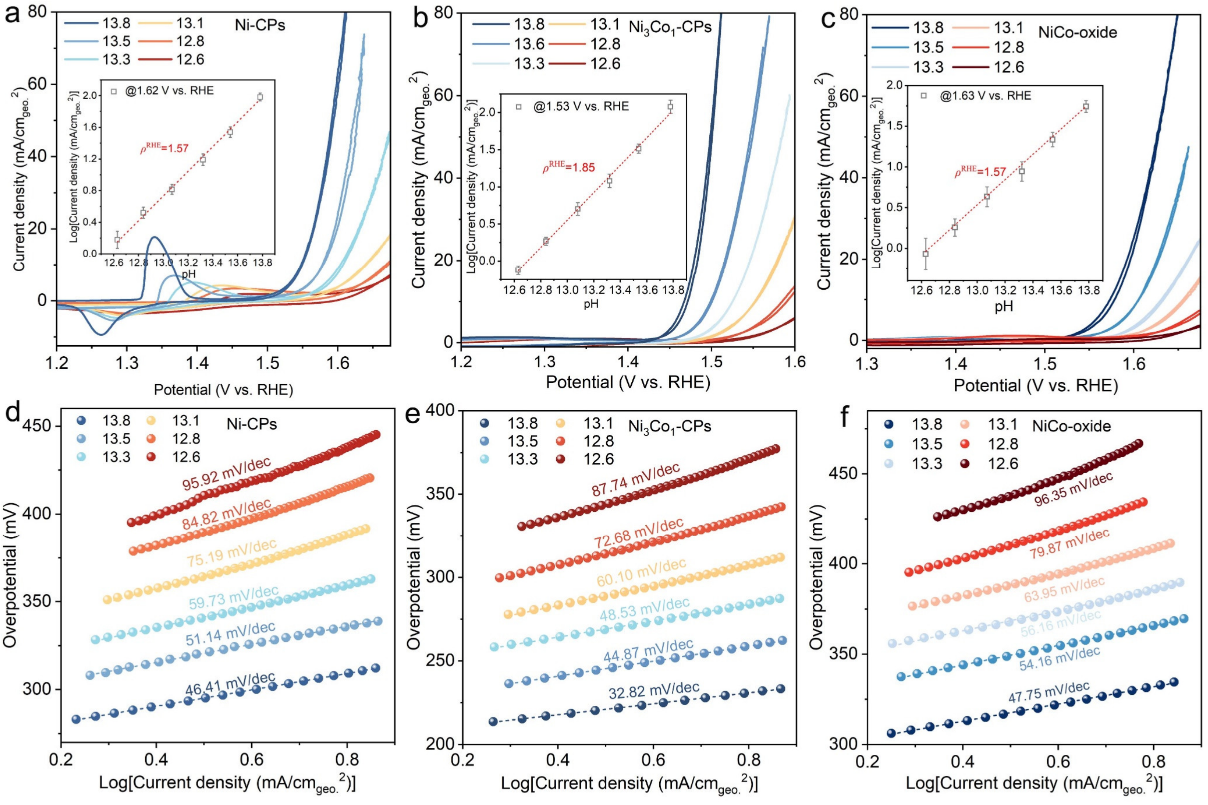

Figure S53. pH dependence of OER activities and Tafel plots of Ni-CPs (a, d), Ni_3Co_1-CPs (b, e), and NiCo-oxide (c, f). The inset plot represents the proton reaction order determined from the equation of ρRHE = (∂log(i)/∂pH).

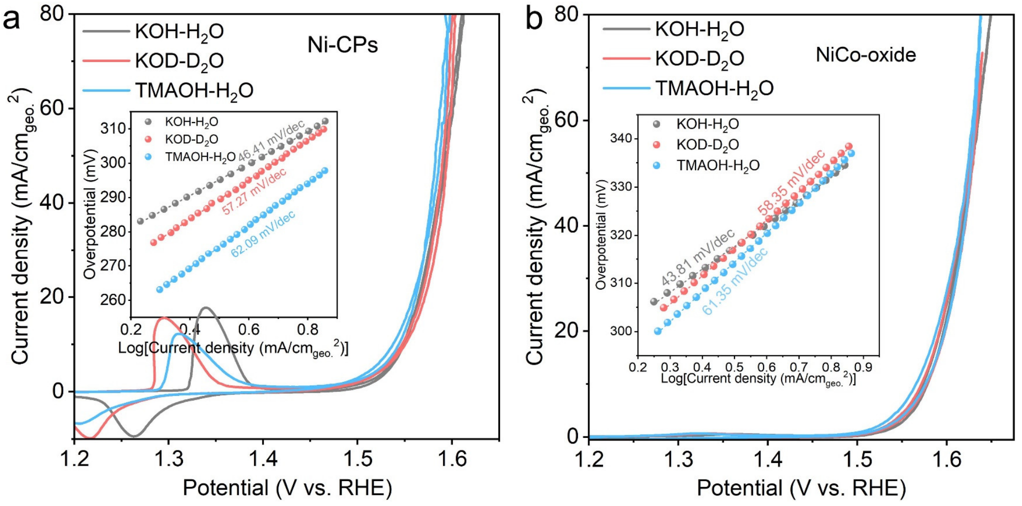

Figure S54. CV curves of Ni-CPs (a) and NiCo-oxide (b) for the OER in 1 M KOH in H_2O, 1 M KOD in D_2O, and 1 M TMAOH in H_2O.

9. Schematic illustration of the OER reaction pathway and free energy calculations

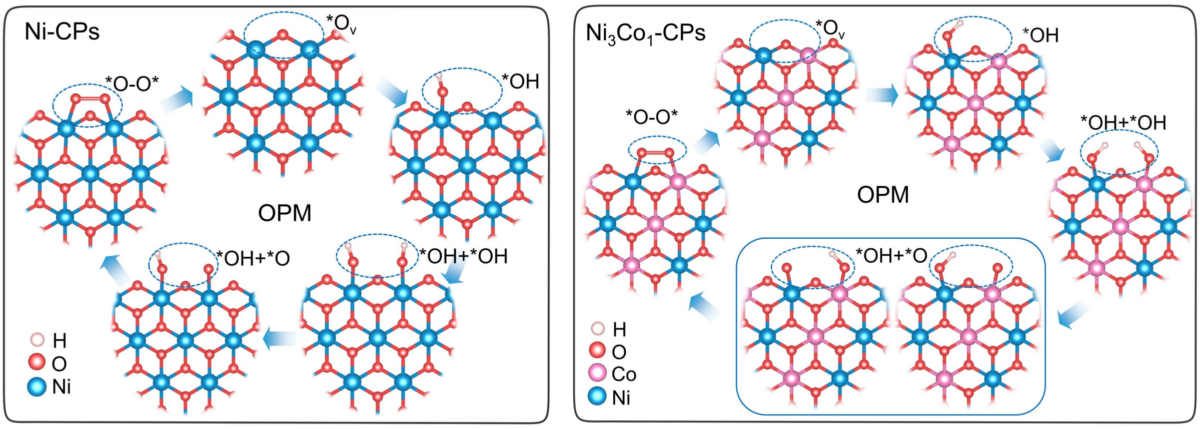

Figure S55. Schematic illustration of the OER cycling processes in Ni-CPs (a) and Ni_3Co_1-CPs (b) with the OPM pathway.

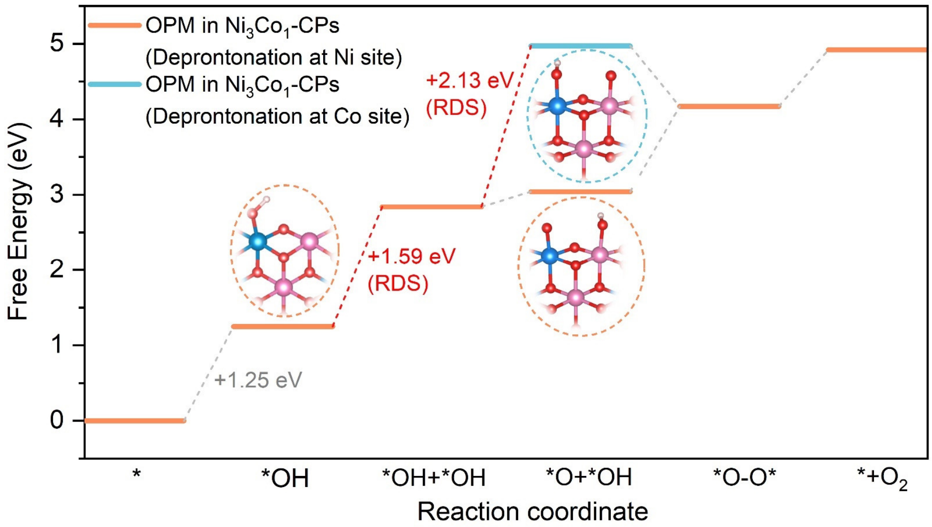

Figure S56. Calculated free energy diagrams of OER intermediates adsorbed on Ni_3Co_1-CPs via the OPM pathway (as shown in **Figure S55b**).

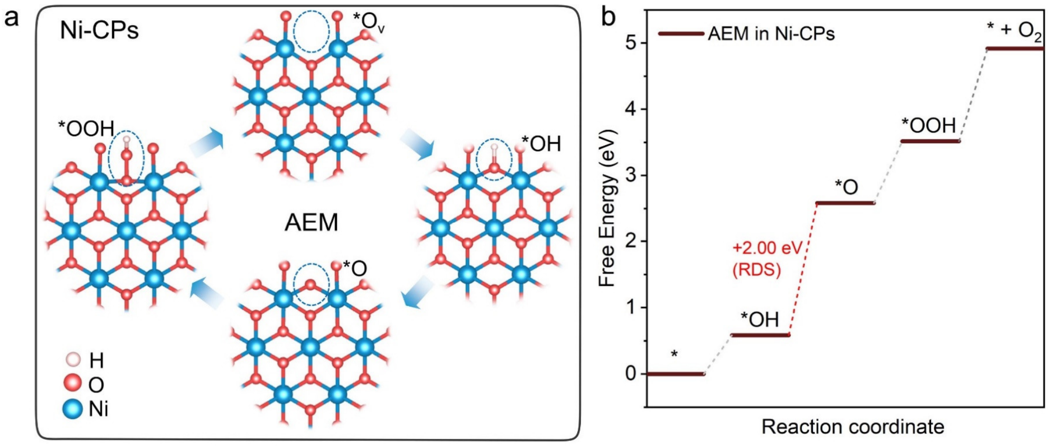

Figure S57. (a) Schematic illustration of the OER cycling processes in Ni-CPs with the AEM pathway. (b) Calculated free energy diagrams of OER intermediates adsorbed on Ni-CPs via the AEM pathway.

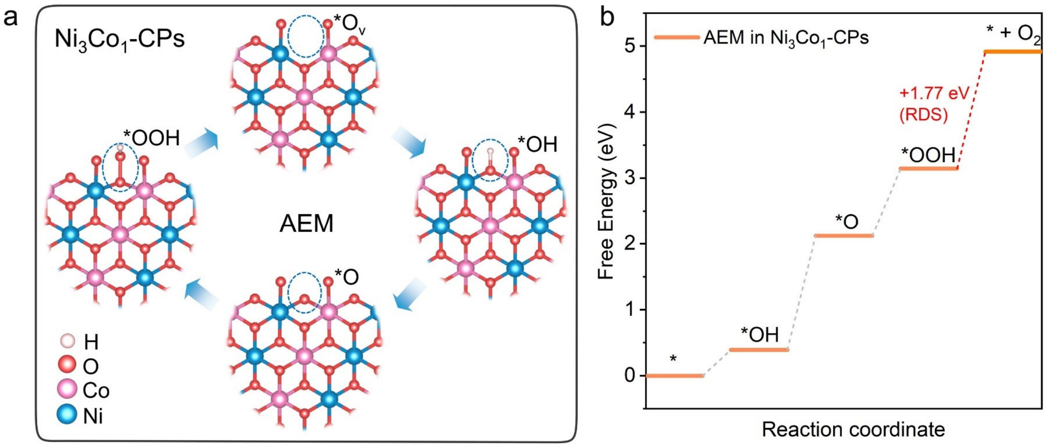

Figure S58. (a) Schematic illustration of the OER cycling processes in Ni_3Co_1-CPs with the AEM pathway. (b) Calculated free energy diagrams of OER intermediates adsorbed on Ni_3Co_1-CPs via the AEM pathway.

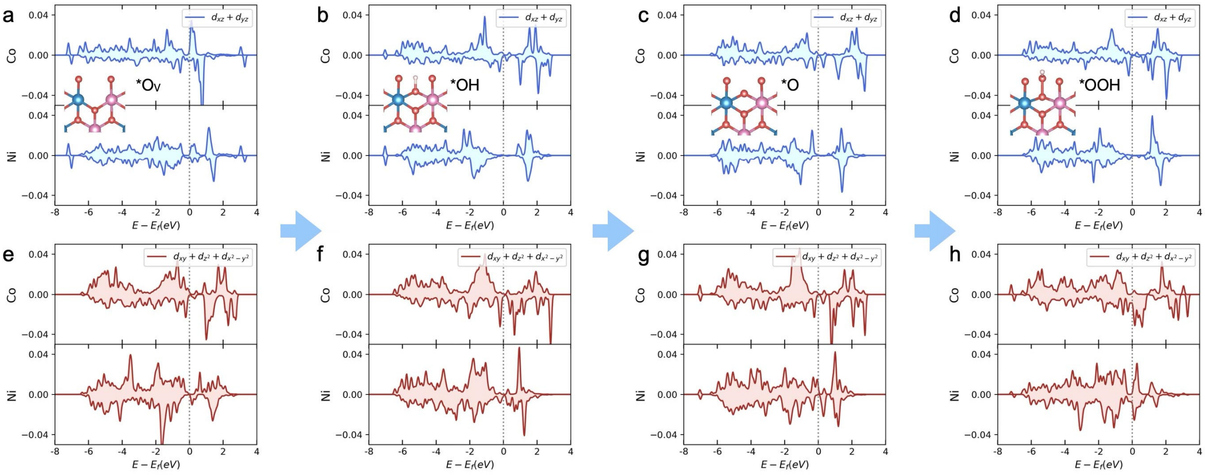

Figure S59. PDOS for Ni and Co centers with the adsorption of key OER intermediates during the AEM mechanism (**Figure S58a**): (a-d) Sum of d_{xz} and d_{yz} orbitals; (e-h) Sum of d_{xy}, d_{z^2}, and $d_{x^2-y^2}$ orbitals.

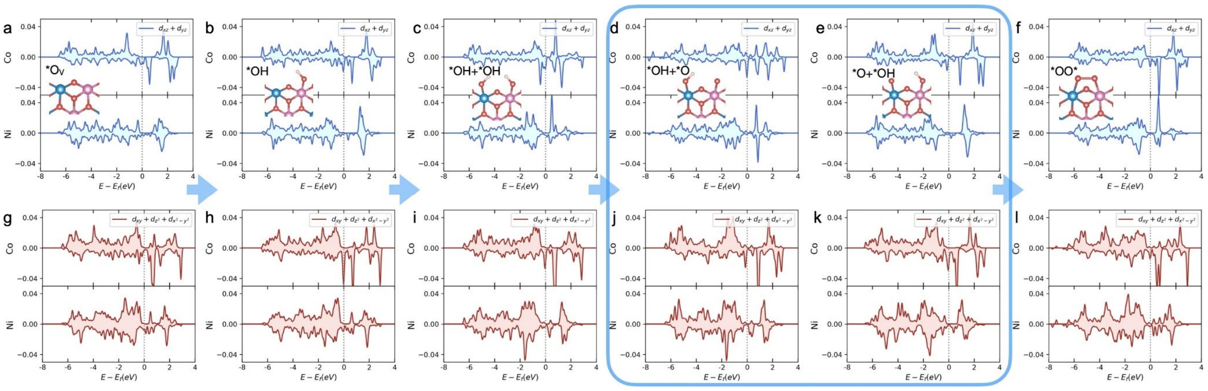

Figure S60. PDOS for Ni and Co centers with the adsorption of key OER intermediates during the OPM mechanism (**Figure 5d**): (a-f) Sum of d_{xz} and d_{yz} orbitals; (g-l) Sum of d_{xy}, d_{z^2}, and $d_{x^2-y^2}$ orbitals.

The calculated projected density of states (PDOS) for key intermediates involved in both AEM and OPM pathways are shown in **Figures S59 and S60**. For both Ni and Co centers, the d_{xz} and d_{yz} orbitals (colored blue), which hybridize with O 2p orbitals, exhibit significant variations, while the d_{z^2}, d_{xy}, and $d_{x^2-y^2}$ orbitals (colored red) remain relatively unchanged across the OER cycling process. In the AEM pathway, the RDS is determined as the deprotonation of the *OOH intermediate to release O_2, accompanied by oxygen vacancy formation.

Correspondingly, the calculated PDOS displays a significant change (**Figure S59a,d**), with both Ni and Co centers exhibiting an increased unoccupied state near the Fermi level. Such a change reflects enhanced electronic instability, making the system more reactive but energetically unfavorable. In contrast, the OPM pathway (**Figure S60a,g**) initiates from a state with the generation of oxygen vacancy, where Co centers exhibit more unoccupied states near the Fermi level than Ni, suggesting that Co centers have a higher tendency to adsorb *OH intermediates to trigger the reaction. Subsequent deprotonation of *OH intermediates can proceed via two routes (**Figure S60d,e,j,k**): (i) deprotonation of the Ni-OH intermediate to form Ni-O + Co-OH; (ii) generation of Ni-OH + Co-O via deprotonation of the Co-OH intermediate. PDOS analysis reveals that both routes lead to reduced spin polarization on the Ni centers; however, the Co center only features a significant decrease in its spin polarization for the generation of Ni-OH + Co-O, which is energetically unfavorable due to the intrinsic tendency of Co to maintain spin polarization. Consequently, this results in a higher energy barrier for the deprotonation of Co-OH intermediate compared to that of Ni-OH. In the final step of the OPM pathway, the desorption of *OO* intermediate leaves oxygen vacancies, and the PDOS of the Co center remains almost identical, indicating minimal charge redistribution. In comparison, the Ni center shows a shift of valence states toward higher energy due to electron loss associated with O_2 release. Compared to the AEM pathway, the aforementioned step in OPM is energetically more favorable, as electron redistribution is localized mainly on the Ni center, while the Co center largely preserves its pristine electronic structure. Overall, combination of PDOS analysis and energy barrier calculations demonstrates that the OPM pathway is energetically more favorable than the AEM pathway, due to the circumvention of the critical high-energy *OOH desorption step. Moreover, the OPM pathway can also minimize unfavorable spin polarization and modulate the local charge rearrangements, particularly at Co sites. These computational insights are consistent with the experimentally observed activity trends in CP catalysts for the OER, supporting OPM as the dominant reaction pathway during the reaction process.

Table S1. At. % ratios of Ni/Co in the as-prepared catalysts based on EDX and ICP-MS analyses.

Samples	at.% ratio of Ni/Co (EDX)	at.% ratio of Ni/Co (ICP-MS)
**Ni_4Co_1-CPs**	3.74:1	3.89:1
**Ni_3Co_1-CPs-RT (No PVP)**	2.50:1	-
**Ni_3Co_1-CPs (No PVP)**	2.48:1	-
**Ni_3Co_1-CPs**	3.05:1	3.07:1
**Ni_2Co_1-CPs**	1.90:1	1.93:1
**NiCo-CPs**	1.10:1	0.95:1
**Ni_3Co_1-oxide**	3.10:1	3.07:1
**NiCo-oxide**	1.03:1	0.95:1
**Ni_3Fe_1-LDH**	2.96:1	-
**Ni_3Co_1-CPs after OER**	1:1	-
**NiCo-CPs after OER**	0:1	-
**NiCo-oxide after OER**	0.98:1	-

Table S2. Fitting parameters of the Ni *K*-edge EXAFS spectra for the as-prepared catalysts and references (CN: coordination numbers; R: interatomic distances; σ^2: Debye-Waller factors; S_0^2: amplitude reduction factor; ΔE: energy changes).

Samples	Path	CN	R	σ^2	S_0^2	ΔE
**Ni foil**	Ni-Ni	12.00	2.48(5)	0.0071(6)	0.90	7.19(2)
**$Ni(OH)_2$**	Ni-O	6.00	2.04(4)	0.0056(4)	0.90	-1.77(2)
	Ni-Ni	6.00	3.12(3)	0.0063(6)		
**γ-NiOOH**	Ni-O	5.0(3)	1.88(7)	0.0084(5)	0.90	2.57(4)
	Ni-Ni	5.2(8)	2.88(0)	0.0079(8)		
**$K_2Ni(CN)_4$**	Ni-C	4.00	1.86(5)	0.0032(3)	0.90	-2.72(5)
	Ni-N	4.00	2.79(4)	0.0063(8)		
	Ni-C-N	8.00	3.06(7)	0.0055(8)		
**Ni-CPs**	Ni-C(N)	4.00	1.87(0)	0.0029(6)	0.90	-0.47(5)
	Ni-N(C)	4.00	3.09(6)	0.0020(5)		
	Ni-C(N)-N(C)	8.00	3.00(6)	0.0079(7)		
**Ni_4Co_1-CPs**	Ni-C(N)	4.00	1.87(0)	0.0031(1)	0.90	-1.61(3)
	Ni-N(C)	4.00	3.13(5)	0.0015(7)		
	Ni-C(N)-N(C)	8.00	3.01(1)	0.0068(6)		
**Ni_3Co_1-CPs**	Ni-C(N)	4.00	1.86(5)	0.0025(6)	0.90	-1.08(6)
	Ni-N(C)	4.00	3.15(6)	0.0019(3)		
	Ni-C(N)-N(C)	8.00	3.00(6)	0.0081(2)		
**Ni_2Co_1-CPs**	Ni-C(N)	4.00	1.86(5)	0.0027(5)	0.90	-0.62(4)
	Ni-N(C)	4.00	3.12(9)	0.0024(9)		
	Ni-C(N)-N(C)	8.00	3.03(2)	0.0075(7)		
**NiCo-CPs**	Ni-C	4.00	1.85(2)	0.0031(4)	0.90	2.38(5)
	Ni-N	4.00	3.13(6)	0.0021(8)		
	Ni-C-N	8.00	2.98(9)	0.0056(6)		
**NiCo-oxide**	Ni-O	5.7(7)	2.04(7)	0.0053(8)	0.90	0.51(6)
	Ni-Ni/Co	11.3(2)	2.95(5)	0.0107(0)		

Table S3. Fitting parameters of the Co *K*-edge EXAFS spectra for the as-prepared catalysts and references (CN: coordination numbers; R: interatomic distances; σ^2: Debye-Waller factors; S_0^2: amplitude reduction factor; ΔE: energy changes).

Samples	Path	CN	R	σ^2	S_0^2	ΔE
**Co foil**	Co-Co	12.00	2.49(3)	0.0066(1)	0.85	7.44(9)
**$Co(OH)_2$**	Co-O	6.00	2.09(6)	0.0067(8)	0.85	-4.08(2)
	Co-Co	6.00	3.17(7)	0.0065(5)		
**Co_3O_4**	Co-O	5.33	1.91(1)	0.0038(8)	0.85	-1.39(5)
	Co_{oct}-Co_{oct}	4.00	2.85(3)	0.0045(3)		
	Co_{oct}-Co_{tet}	8.00	3.35(9)	0.0080(0)		
**CoOOH**	Co-O	6.00	1.89(9)	0.0037(5)	0.85	-1.47(4)
	Co-Co	6.00	2.85(4)	0.0040(8)		
**$K_3Co(CN)_6$**	Co-C	6.00	1.89(1)	0.0020(8)	0.85	-1.67(9)
	Co-N	6.00	3.10(0)	0.0019(4)		
	Co-C-N	12.00	3.10(0)	0.0064(8)		
**Ni_4Co_1-CPs**	Co-N/O	6.00	1.97(1)	0.0064(4)	0.85	3.62(8)
	Co-C	4.00	3.24(9)	0.0021(9)		
	Co-N-C	8.00	3.13(3)	0.0069(0)		
**Ni_3Co_1-CPs**	Co-N/O	6.00	1.97(2)	0.0066(4)	0.85	-1.14(8)
	Co-C	4.00	3.25(9)	0.0021(5)		
	Co-N-C	8.00	3.11(7)	0.0065(6)		
**Ni_2Co_1-CPs**	Co-N/O	6.00	1.96(9)	0.0052(9)	0.85	4.49(9)
	Co-C	4.00	3.24(9)	0.0024(9)		
	Co-N-C	8.00	3.14(3)	0.0040(8)		
**NiCo-CPs**	Co-N/O	6.00	1.96(6)	0.0055(4)	0.85	2.98(2)
	Co-C	4.00	3.24(6)	0.0026(4)		
	Co-N-C	8.00	3.10(6)	0.0050(0)		
**NiCo-oxide**	Co-O	5.1(3)	1.90(9)	0.0042(4)	0.85	-0.38(7)
	Co_{oct}-Co_{oct}	4.00	2.86(7)	0.0050(1)		
	Co_{oct}-Co_{tet}	5.5(1)	3.36(7)	0.0050(1)		

Table S4. Comparison of OER performance of the as-prepared catalysts with recent representative studies.

**Electrode materials**	**Overpotential at 10 m/cm$_{geo.}^2$**	**Tafel slope (mV/dec)**	**Stability**	**Substrate**	**Ref.**
CoNi-PI (3,4-dihydroxyphthalimide)	205 mV	42	50 h	Carbon paper	[13]
Monolayer NiCo hydroxide	208 mV	-	180 h	GC-RDE	[14]
$Fe_{0.5}Co_{0.5}OOH$ nanosheet arrays	227 mV	32.74	24 h	Carbon paper	[15]
$Co_5Fe_3Cr_2$-LDH	232 mV	31	168 h	GC-RDE	[16]
CoFe LDHs-Ar	237 mV	37.85	-	GC-RDE	[17]
CoFe LDH hollow nanocages	238 mV	42	50 h	Ni foam	[18]
$Co_{80}Fe_{20}(OH)(OCH_3)$	240 mV	53.45	10 h	GC-RDE	[19]
Fe-CoMo ultrathin hydroxide	245 mV	37	90 h	Ni foam	[20]
Fe-$_{0.15}Co_9S_8$	255 mV	49	12 h	GC-RDE	[21]
Fe-Co-O nanosheets	260 mV	53	16 h	GC-RDE	[22]
Fe-Co_3O_4 nanoplates	262 mV	43	50 h	GC-RDE	[23]
CoFe/WP	267 mV	35	100 h	GC-RDE	[24]
FeCoNi-LDHs	269 mV	42.34	100 h	GC-RDE	[25]
Fe-Co-P nanoboxes	269 mV	31	100 h	Carbon paper	[26]
$Fe_{0.4}Co_{0.6}Se_2$ nanoframes	270 mV	36	24 h	GC-RDE	[27]
$LiFe_{0.55}Co_{0.45}(H_2O)_2[BP_2O_8]\cdot H_2O$	270 mV	28	120 h	FTO	[28]
$NiCo_{1.88}Fe_{0.12}O_4$ nanoboxes	274 mV	42	30 h	Carbon paper	[29]
Co–Fe–S@PB nanoboxes	286 mV	37.84	33 h	GC-RDE	[30]
F-doped $NiCo_2O_4$	300 mV	96	108 h	GC-RDE	[31]
CoNi-NBs	300 mV	49.43	40 h	GC-RDE	[32]
$ZnCo_{1.2}Ni_{0.8}O_4$	311 mV	61.19	12 h	GC-RDE	[33]
Ni-CoS_2	316 mV	66	-	GC-RDE	[34]
CoTAPP-Ni-MF-2DP	450 mV @ 0.45 mA/cm^2	60.1	20 h	ITO	[35]
**Ni-CPs**	**319 mV**	**46.41**	**200 h**	**GC-RDE**	**This work**
**Ni_3Co_1-CPs**	**239 mV**	**32.82**	**4000 h**	**GC-RDE**	**This work**
**NiCo-CPs**	**336 mV**	**44.62**	**200 h**	**GC-RDE**	**This work**
**NiCo-oxide**	**346 mV**	**47.75**	**200 h**	**GC-RDE**	**This work**
**Ni_3Fe_1-LDH**	**253 mV**	**44.61**	**200 h**	**GC-RDE**	**This work**

Table S5. ICP-MS results of freshly prepared electrolytes before and after the OER measurements. (*Note: For ICP-MS characterizations, the as-prepared catalyst inks were drop-dried on carbon paper (loading mass of ~1 mg/cm^2). Chronopotentiometry tests were then performed in 50 mL 1 M KOH and maintained at a constant potential for at least 1 h. Then, 2 mL of electrolytes were sampled for ICP-MS analysis.*)

**Samples**	**Fe (ppb)**	**Co (ppb)**	**Ni (ppb)**
**Fresh 1 M KOH**	0.32	0.13	0.19
**Ni-CPs (immersion into the electrolyte)**	0.22	0.10	**151.37**
**Ni-CPs at 0.9 V vs. RHE**	0.33	0.11	**154.77**
**Ni-CPs at 1.4 V vs. RHE**	0.23	0.06	**156.83**
**Ni-CPs at 1.5 V vs. RHE**	0.35	0.06	**152.12**
**Ni-CPs at 1.55 V vs. RHE**	0.31	0.04	**152.35**
**Ni_3Co_1-CPs (immersion into the electrolyte)**	0.60	3.24	**456.79**
**Ni_3Co_1-CPs at 0.9 V vs. RHE**	0.51	4.54	**535.08**
**Ni_3Co_1-CPs at 1.4 V vs. RHE**	0.78	4.29	**538.56**
**Ni_3Co_1-CPs at 1.45 V vs. RHE**	0.60	3.71	**551.08**
**Ni_3Co_1-CPs at 1.5 V vs. RHE**	0.65	2.77	**521.57**
**NiCo-CPs (immersion into the electrolyte)**	0.81	23.78	**360.66**
**NiCo-CPs at 0.9 V vs. RHE**	0.69	25.15	**488.40**
**NiCo-CPs at 1.4 V vs. RHE**	0.59	22.09	**423.83**
**NiCo-CPs at 1.5 V vs. RHE**	0.85	21.09	**452.91**
**NiCo-CPs at 1.55 V vs. RHE**	0.73	21.39	**458.97**
**NiCo-oxide (immersion into the electrolyte)**	0.16	0.05	0.18
**NiCo-oxide at 0.9 V vs. RHE**	0.43	0.04	0.52
**NiCo-oxide at 1.4 V vs. RHE**	0.85	0.13	0.98
**NiCo-oxide at 1.5 V vs. RHE**	0.73	0.08	0.55
**NiCo-oxide at 1.55 V vs. RHE**	0.60	0.08	0.90

Table S6. Fitting parameters of *operando* Ni *K*-edge EXAFS spectra of Ni-CPs for the OER (CN: coordination numbers; R: interatomic distances; σ^2: Debye-Waller factors; S_0^2: amplitude reduction factor; ΔE: energy changes).

Samples	Path	CN	R	σ^2	S_0^2	ΔE
**Electrolyte**	Ni-C(N)	3.8(5)	1.89(5)	0.0083(5)	0.90	-8.65(1)
	Ni-O	2.0(2)	2.05(6)	0.0025(6)		
	Ni-Ni	2.5(3)	3.06(3)	0.0070(6)		
	Ni-N(C)/C-N	3.8(0)	3.12(7)	0.0030(4)		
**1.0 V vs. RHE**	Ni-O	4.6(5)	2.06(1)	0.0080(1)	0.90	-2.00(9)
	$Ni-Ni_1$	0.6(0)	2.66(6)	0.0084(9)		
	$Ni-Ni_2$	4.8(2)	3.11(5)	0.0077(1)		
**1.2 V vs. RHE**	Ni-O	4.9(3)	2.06(3)	0.0060(8)	0.90	-1.49(2)
	$Ni-Ni_1$	1.0(0)	2.67(4)	0.0090(8)		
	$Ni-Ni_2$	4.0(1)	3.12(3)	0.0083(7)		
**1.4 V vs. RHE**	Ni-O	2.1(8)	1.94(6)	0.0045(7)	0.90	1.13(7)
	Ni-O	2.6(6)	2.14(9)	0.0080(0)		
	$Ni-Ni_1$	1.8(5)	2.84(9)	0.0083(9)		
	$Ni-Ni_2$	2.0(5)	3.13(4)	0.0069(6)		
**1.5 V vs. RHE**	Ni-O	4.5(2)	1.93(6)	0.0054(2)	0.90	0.14(8)
	Ni-O	1.6(4)	2.18(1)	0.0035(2)		
	$Ni-Ni_1$	4.4(8)	2.86(2)	0.0080(1)		
	$Ni-Ni_2$	1.4(9)	3.12(1)	0.0052(7)		
**1.52 V vs. RHE**	Ni-O	4.8(2)	1.86(8)	0.0063(3)	0.90	-0.86(4)
	Ni-Ni	5.1(3)	2.84(8)	0.0067(1)		
**Back to 1.0 V vs. RHE**	Ni-O	5.5(1)	2.06(4)	0.0071(3)	0.90	-1.96(7)
	Ni-Ni	6.00	3.11(3)	0.0092(8)		

(**Note**: The fitting of second shells of $Ni-Ni_1$ and $Ni-Ni_2$ was calculated based on a typical backscattering path of the second shell Ni-O-Ni with interatomic distances of 2.820 and 3.120 Å, respectively.)

Table S7. Fitting parameters of *operando* Ni *K*-edge EXAFS spectra of Ni_3Co_1-CPs for the OER (CN: coordination numbers; R: interatomic distances; σ^2: Debye-Waller factors; S_0^2: amplitude reduction factor; ΔE: energy changes).

Samples	Path	CN	R	σ^2	S_0^2	ΔE
**Electrolyte**	Ni-C(N)	4.0(3)	1.86(1)	0.0091(3)	0.90	-9.86(9)
	Ni-O	1.1(0)	2.05(9)	0.0027(2)		
	Ni-Ni/Co	2.8(8)	3.01(7)	0.0066(7)		
	Ni-N(C)/C-N	8.5(3)	3.11(1)	0.0014(0)		
**0.9 V vs. RHE**	Ni-C(N)	3.9(5)	1.86(6)	0.0086(2)	0.90	-8.79(6)
	Ni-O	1.1(2)	2.06(5)	0.0018(3)		
	Ni-Ni/Co	2.8(3)	3.03(1)	0.0063(5)		
	Ni-N(C)/C-N	8.4(8)	3.12(3)	0.0021(1)		
**1.0 V vs. RHE**	Ni-C(N)	3.8(8)	1.86(3)	0.0085(8)	0.90	-9.60(9)
	Ni-O	1.2(1)	2.06(1)	0.0029(9)		
	Ni-Ni/Co	3.0(6)	3.01(7)	0.0071(8)		
	Ni-N(C)/C-N	8.6(2)	3.11(3)	0.0014(7)		
**1.1 V vs. RHE**	Ni-C(N)	3.6(9)	1.86(5)	0.0081(9)	0.90	-9.16(3)
	Ni-O	1.2(4)	2.05(9)	0.0026(7)		
	Ni-Ni/Co	3.2(8)	3.02(6)	0.0065(9)		
	Ni-N(C)/C-N	8.5(5)	3.12(4)	0.0016(8)		
**1.2 V vs. RHE**	Ni-C(N)	3.4(2)	1.86(3)	0.0076(2)	0.90	-8.88(6)
	Ni-O	1.3(2)	2.05(4)	0.0032(5)		
	Ni-Ni/Co	3.3(4)	3.03(2)	0.0063(6)		
	Ni-N(C)/C-N	8.4(2)	3.13(1)	0.0018(6)		
**1.3 V vs. RHE**	Ni-C(N)	3.4(6)	1.86(3)	0.0081(7)	0.90	-9.35(0)
	Ni-O	1.3(9)	2.04(9)	0.0035(0)		
	Ni-Ni/Co	3.4(1)	3.02(2)	0.0067(6)		
	Ni-N(C)/C-N	8.5(6)	3.12(2)	0.0013(8)		
**1.35 V vs. RHE**	Ni-C(N)	3.1(8)	1.87(3)	0.0074(0)	0.90	-5.45(2)
	Ni-O	2.2(0)	2.04(2)	0.0060(9)		
	Ni-Ni/Co	3.3(8)	3.07(3)	0.0048(5)		
	Ni-N(C)/C-N	7.7(2)	3.20(3)	0.0042(5)		
**1.4 V vs. RHE**	Ni-C(N)	2.2(1)	1.83(4)	0.0024(4)	0.90	-0.23(9)
	Ni-O	2.3(9)	1.94(7)	0.0017(7)		
	Ni-O	2.4(3)	2.08(7)	0.0057(0)		
	$Ni-Ni_1/Co$	1.9(2)	2.80(4)	0.0082(9)		
	$Ni-Ni_2$	2.5(6)	3.09(7)	0.0053(7)		
**1.45 V vs. RHE**	Ni-O	3.6(3)	1.93(5)	0.0082(9)	0.90	1.85(5)
	Ni-O	1.0(9)	2.09(4)	0.0061(4)		
	$Ni-Ni_1/Co$	2.7(9)	2.83(7)	0.0066(3)		
	$Ni-Ni_2$	1.6(5)	3.09(4)	0.0046(7)		
**1.475 V vs. RHE**	Ni-O	4.8(2)	1.91(3)	0.0058(1)	0.90	-0.55(6)
	Ni-O	0.9(9)	2.12(3)	0.0045(8)		
	$Ni-Ni_1/Co$	4.1(3)	2.82(9)	0.0051(7)		
	$Ni-Ni_2$	1.4(0)	3.08(1)	0.0031(3)		
**1.50 V vs. RHE**	Ni-O	5.3(3)	1.90(9)	0.0053(3)	0.90	-1.53(5)
	Ni-O	0.6(5)	2.17(0)	0.0043(0)		
	$Ni-Ni_1/Co$	5.1(1)	2.83(1)	0.0049(3)		
	$Ni-Ni_2$	1.2(7)	3.08(2)	0.0031(5)		
**Removed potential**	Ni-O	3.8(5)	1.94(0)	0.0073(0)	0.90	1.50(6)
	Ni-O	1.3(3)	2.08(6)	0.0056(8)		
	$Ni-Ni_1/Co$	3.4(2)	2.84(8)	0.0065(5)		
	$Ni-Ni_2$	2.2(0)	3.08(8)	0.0035(6)		

Table S8. Fitting parameters of *operando* Co *K*-edge EXAFS spectra of Ni_3Co_1-CPs for the OER (CN: coordination numbers; R: interatomic distances; σ^2: Debye-Waller factors; S_0^2: amplitude reduction factor; ΔE: energy changes). (**Note:** *Operando* quick-XAS experiments were performed at the SuperXAS-X10DA beamline.)

Samples	Path	CN	R	σ^2	S_0^2	ΔE
**Electrolyte**	Co-O/N	2.4(9)	1.96(2)	0.0043(5)	0.85	-1.94(0)
	Co-O	2.4(7)	2.12(9)	0.0038(7)		
	Co-Co/Ni	4.8(8)	3.13(0)	0.0081(4)		
**0.9 V vs. RHE**	Co-O/N	2.4(8)	1.96(6)	0.0040(4)	0.85	-1.43(0)
	Co-O	2.3(7)	2.13(2)	0.0036(3)		
	Co-Co	4.9(4)	3.13(3)	0.0080(5)		
**1.0 V vs. RHE**	Co-O/N	2.5(6)	1.96(8)	0.0047(5)	0.85	-1.47(4)
	Co-O	2.3(8)	2.13(4)	0.0042(4)		
	Co-Co/Ni	4.9(0)	3.13(5)	0.0084(8)		
**1.1 V vs. RHE**	Co-O/N	2.4(1)	1.96(1)	0.0048(7)	0.85	-1.87(2)
	Co-O	2.5(5)	2.12(8)	0.0038(0)		
	Co-Co/Ni	5.1(4)	3.14(1)	0.0080(8)		
**1.2 V vs. RHE**	Co-O/N	2.0(4)	1.94(1)	0.0036(2)	0.85	-1.49(8)
	Co-O	2.9(1)	2.11(8)	0.0032(3)		
	Co-Co/Ni	5.0(0)	3.14(0)	0.0078(2)		
**1.3 V vs. RHE**	Co-O/N	2.1(5)	1.93(9)	0.0044(9)	0.85	-1.18(5)
	Co-O	2.8(5)	2.12(1)	0.0040(7)		
	Co-Co/Ni	5.0(4)	3.14(3)	0.0079(6)		
**1.35 V vs. RHE**	Co-O	2.8(9)	1.91(3)	0.0045(1)	0.85	-2.07(9)
	Co-O	2.2(5)	2.11(6)	0.0032(4)		
	Co-Co_1/Ni	0.9(3)	2.83(4)	0.0022(4)		
	Co-Co_2	4.9(7)	3.13(0)	0.0087(6)		
**1.4 V vs. RHE**	Co-O	3.7(8)	1.90(2)	0.0032(9)	0.85	-0.80(8)
	Co-O	1.2(7)	2.13(8)	0.0032(1)		
	Co-Co_1/Ni	2.1(7)	2.83(5)	0.0030(0)		
	Co-Co_2	2.7(5)	3.13(3)	0.0060(9)		
**1.45 V vs. RHE**	Co-O	5.4(0)	1.89(4)	0.0031(5)	0.85	-0.01(5)
	Co-O	0.7(4)	2.18(2)	0.0064(4)		
	Co-Co_1/Ni	3.9(3)	2.83(5)	0.0032(9)		
	Co-Co_2	1.5(7)	3.11(4)	0.0055(2)		
**1.475 V vs. RHE**	Co-O	6.0(0)	1.88(9)	0.0027(9)	0.85	-0.56(3)
	Co-Co_1/Ni	5.4(6)	2.83(8)	0.0036(5)		
	Co-Co_2	0.8(2)	3.10(7)	0.0021(2)		
**1.5 V vs. RHE**	Co-O	6.0(0)	1.88(5)	0.0026(7)	0.85	-0.78(9)
	Co-Co_1/Ni	5.4(6)	2.83(3)	0.0031(9)		
	Co-Co_2	0.8(2)	3.10(3)	0.0023(5)		
**Removed potential**	Co-O_1	5.8(7)	1.88(9)	0.0025(8)	0.85	-0.90(8)
	Co-Co_1/Ni	4.9(2)	2.83(9)	0.0034(6)		
	Co-Co_2	0.8(7)	3.09(5)	0.0021(8)		

(**Note**: The fitting of second shells of Co-Co_1 and Co-Co_2 was calculated based on a typical backscattering path of the second shell Co-O-Co with interatomic distances of 2.851 and 3.173 Å, respectively.)

Table S9. Fitting parameters of *operando* Co *K*-edge EXAFS spectra of NiCo-CPs for the OER (CN: coordination numbers; R: interatomic distances; σ^2: Debye-Waller factors; S_0^2: amplitude reduction factor; ΔE: energy changes). (**Note:** *Operando* XAS experiments were performed at the KMC-2 beamline.)

Samples	Path	CN	R	σ^2	S_0^2	ΔE
**Electrolyte**	Co-O/N	2.4(8)	1.91(1)	0.0040(9)	0.85	-2.25(1)
	Co-O	3.3(6)	2.08(3)	0.0025(2)		
	Co-Co	5.1(5)	3.10(2)	0.0062(3)		
**1.0 V vs. RHE**	Co-O/N	4.3(6)	1.90(6)	0.0050(0)	0.85	-2.08(5)
	Co-O	1.1(8)	2.12(5)	0.0020(6)		
	$Co-Co_1$	2.1(3)	2.83(7)	0.0049(4)		
	$Co-Co_2$	2.4(3)	3.10(5)	0.0055(6)		
**1.2 V vs. RHE**	Co-O	4.4(7)	1.89(2)	0.0038(0)	0.85	-1.31(1)
	$Co-Co_1$	3.4(1)	2.82(6)	0.0040(3)		
	$Co-Co_2$	1.1(2)	3.11(5)	0.0036(6)		
**1.4 V vs. RHE**	Co-O	5.3(9)	1.88(6)	0.0026(9)	0.85	-1.83(5)
	Co-Co	4.5(9)	2.83(0)	0.0025(6)		
**1.5 V vs. RHE**	Co-O	5.8(7)	1.88(5)	0.0030(4)	0.85	-1.51(0)
	Co-Co	5.4(9)	2.82(6)	0.0038(7)		
**1.55 V vs. RHE**	Co-O	5.9(0)	1.89(5)	0.0032(5)	0.85	-1.33(1)
	Co-Co	5.6(1)	2.82(9)	0.0041(0)		
**Back to 1.0 V vs. RHE**	Co-O	5.8(1)	1.89(3)	0.0035(6)	0.85	-2.50(0)
	Co-Co	4.5(6)	2.84(8)	0.0049(7)		

Supplementary references

1. L. Trotochaud, S. L. Young, J. K. Ranney, S. W. Boettcher, *J. Am. Chem. Soc.* **2014**, *136*, 6744.
2. Y. Zhao, W. Wan, R. Erni, L. Pan, G. R. Patzke, *Angew. Chem. Int. Ed.* **2024**, *63*, e202400048.
3. Y. Zhao, N. Dongfang, C. A. Triana, C. Huang, R. Erni, W. Wan, J. Li, D. Stoian, L. Pan, P. Zhang, J. Lan, M. Iannuzzi, G. R. Patzke, *Energy Environ. Sci.* **2022**, *15*, 727.
4. T. D. Kühne, M. Iannuzzi, M. Del Ben, V. V. Rybkin, P. Seewald, F. Stein, T. Laino, R. Z. Khaliullin, O. Schütt, F. Schiffmann, et al. *J. Chem. Phys.* **2020**, *152*, 194103.
5. S. Goedecker, M. Teter, J. Hutter, *Phys. Rev. B* **1996**, *54*, 1703-1710.
6. J. Perdew, K. Burke, M. Ernzerhof, *Phys. Rev. Lett.* **1996**, *77*, 3865–3868.
7. J. Moellmann, G. Stefan, *J Phys. Chem. C* **2014**, 118, 7615.
8. J. D. Head, M. C. Zerner, *Chem. Phys. Lett.* **1985**. *122*, 264.
9. S. J. Hibble, A. M. Chippindale, A. H. Pohl, A. C. Hannon, *Angew. Chem. Int. Ed.* **2007**, *46*, 7116–7118.
10. C. Kuai, Z. Xu, C. Xi, A. Hu, Z. Yang, Y. Zhang, C.J. Sun, L. Li, D. Sokaras, C. Dong, et al. *Nat. Catal.* **2020**, *3*, 743.
11. C. Kuai, C. Xi, A. Hu, Y. Zhang, Z. Xu, D. Nordlund, C. J. Sun, C. A. Cadigan, R. M. Richards, L. Li, et al. *J. Am. Chem. Soc.* **2021**, *143*, 18519.
12. F. T. Haase, E. Ortega, S. Saddeler, F. Schmidt, D. Cruz, F. Scholten, M. Rüscher, A. Martini, H. S. Jeon, A. Herzog, et al. *Energy Environ. Sci.* **2024**, *17*, 2046.
13. Y. Weng, L. Sun, H. Jia, W. Shang, J. J. Chen, B. Dhara, Y. Chen, F. Huang, S. Han, H. He, B. Yin, *J. Am. Chem. Soc.* **2025**, *147*, 13928.
14. J. Kang, X. Qiu, Q. Hu, J. Zhong, X. Gao, R. Huang, C. Wan, L. M. Liu, X. Duan, L. Guo, *Nat. Catal.* **2021**, *4*, 1050.
15. S. Ye, Y. Lei, T. Xu, L. Zheng, Z. Chen, X. Yang, X. Ren, Y. Li, Q. Zhang, J. Liu, *Appl. Catal. B-Environ.* **2022**, *304*, 120986.
16. J. Chen, H. Li, S. Chen, J. Fei, C. Liu, Z. Yu, K. Shin, Z. Liu, L. Song, G. Henkelman, L. Wei, Y. Chen, *Adv. Energy Mater.* **2021**, *11*, 2003412.
17. Y. Wang, Y. Zhang, Z. Liu, C. Xie, S. Feng, D. Liu, M. Shao, S. Wang, *Angew. Chem. Int. Ed.* **2017**, *56*, 5867.
18. Y. Ni, D. Shi, B. Mao, S. Wang, Y. Wang, A. Ahmad, J. Sun, F. Song, M. Cao, C. Hu, *Small* **2023**, *19*, 2302556.
19. J. He, Y. Liu, Y. Huang, H. Li, Y. Zou, C. L. Dong, S. Wang, *Adv. Funct. Mater.* **2021**, *31*, 2009245.
20. L. Zeng, B. Cao, X. Wang, H. Liu, J. Shang, J. Lang, X. Cao, H. Gu, *Nanoscale* **2021**, *13*, 3153.
21. Z. Wang, Z. Lin, J. Deng, S. Shen, F. Meng, J. Zhang, Q. Zhang, W. Zhong, L. Gu, *Adv. Energy Mater.* **2021**, *11*, 2003023.
22. Q. Wang, X. Xue, Y. Lei, Y. Wang, Y. Feng, X. Xiong, D. Wang, Y. Li, *Small* **2020**, *16*, 2001571.
23. S. L. Zhang, B. Y. Guan, X. F. Lu, S. Xi, Y. Du, X. W. Lou, *Adv. Mater.* **2020**, *32*, 2002235.
24. Y. Zhu, G. Chen, Y.-C. Chu, C.-S. Hsu, J. Wang, C.-W. Tung, H. M. Chen, *Angew. Chem. Int. Ed.* **2022**, *61*, e202211142.
25. X. Zhang, F. Yan, X. Ma, C. Zhu, Y. Wang, Y. Xie, S.-L. Chou, Y. Huang, Y. Chen, *Adv. Energy Mater.* **2021**, *11*, 2102141.
26. H. Zhang, W. Zhou, J. Dong, X. F. Lu, X. W. Lou, *Energy Environ. Sci.* **2019**, *12*, 3348.
27. J. Zhang, Y. Yan, B. Mei, R. Qi, T. He, Z. Wang, W. Fang, S. Zaman, Y. Su, S. Ding, B. Y. Xia, *Energy Environ. Sci.* **2021**, *14*, 365.
28. L. Reith, J. N. Hausmann, S. Mebs, I. Mondal, H. Dau, M. Driess, P. W. Menezes, *Adv. Energy Mater.* **2023**, *13*, 2203886.

29. Y. Huang, S. L. Zhang, X. F. Lu, Z. P. Wu, D. Luan, X. W. D. Lou, *Angew. Chem. Int. Ed.* **2021**, *60*, 11841.
30. Y. Zhao, C. K. Mavrokefalos, P. Zhang, R. Erni, J. Li, C. A. Triana, G. R. Patzke, *Chem. Mater.* **2020**, *32*, 1371.
31. Y. Yue, X. Zhong, M. Sun, J. Du, W. Gao, W. Hu, C. Zhao, J. Li, B. Huang, Z. Li, C. Li, *Adv. Mater.* **2025**, 2418058.
32. Y. Zhao, N. Dongfang, C. Huang, R. Erni, J. Li, H. Zhao, L. Pan, G. R. Patzke, *Nat. Commun.* **2025**, *16*, 580.
33. Y. Duan, S. Sun, Y. Sun, S. Xi, X. Chi, Q. Zhang, X. Ren, J. Wang, S. J. H. Ong, Y. Du, L. Gu, A. Grimaud, Z. J. Xu, *Adv. Mater.* **2019**, *31*, e1807898.
34. W. Peng, A. Deshmukh, N. Chen, Z. Lv, S. Zhao, J. Li, B. Yan, X. Gao, L. Shang, Y. Gong, L. Wu, *ACS Catal.* **2022**, *12,* 3743.
35. D. Fa, J. Yuan, G. Feng, S. Lei, W. Hu, *Angew. Chem. Int. Ed.* **2023**, *62*, e202300532.
